# Single-cell transcriptomics and chromatin accessibility profiling elucidate the kidney-protective mechanism of mineralocorticoid receptor antagonists

**DOI:** 10.1172/JCI157165

**Published:** 2024-01-02

**Authors:** Amin Abedini, Andrea Sánchez-Navaro, Junnan Wu, Konstantin A. Klötzer, Ziyuan Ma, Bibek Poudel, Tomohito Doke, Michael S. Balzer, Julia Frederick, Hana Cernecka, Hongbo Liu, Xiujie Liang, Steven Vitale, Peter Kolkhof, Katalin Susztak

**Affiliations:** 1 Renal, Electrolyte, and Hypertension Division, Department of Medicine,; 2 Institute for Diabetes, Obesity, and Metabolism, and; 3 Department of Genetics, University of Pennsylvania, Perelman School of Medicine, Philadelphia, Pennsylvania, USA.; 4 Bayer AG, Pharmaceuticals, Research and Development, Cardiovascular Research, Wuppertal, Germany.

**Keywords:** Genetics, Nephrology, Chronic kidney disease, Molecular genetics

## Abstract

Mineralocorticoid excess commonly leads to hypertension (HTN) and kidney disease. In our study, we used single-cell expression and chromatin accessibility tools to characterize the mineralocorticoid target genes and cell types. We demonstrated that mineralocorticoid effects were established through open chromatin and target gene expression, primarily in principal and connecting tubule cells and, to a lesser extent, in segments of the distal convoluted tubule cells. We examined the kidney-protective effects of steroidal and nonsteroidal mineralocorticoid antagonists (MRAs), as well as of amiloride, an epithelial sodium channel inhibitor, in a rat model of deoxycorticosterone acetate, unilateral nephrectomy, and high-salt consumption–induced HTN and cardiorenal damage. All antihypertensive therapies protected against cardiorenal damage. However, finerenone was particularly effective in reducing albuminuria and improving gene expression changes in podocytes and proximal tubule cells, even with an equivalent reduction in blood pressure. We noted a strong correlation between the accumulation of injured/profibrotic tubule cells expressing secreted posphoprotein 1 (*Spp1*), *Il34*, and platelet-derived growth factor subunit b (*Pdgfb*) and the degree of fibrosis in rat kidneys. This gene signature also showed a potential for classifying human kidney samples. Our multiomics approach provides fresh insights into the possible mechanisms underlying HTN-associated kidney disease, the target cell types, the protective effects of steroidal and nonsteroidal MRAs, and amiloride.

## Abstract 2

A multi-omics approach reveals the cell types and gene expression changes that drive hypertension and the development of hypertensive kidney disease.

## Graphical abstract

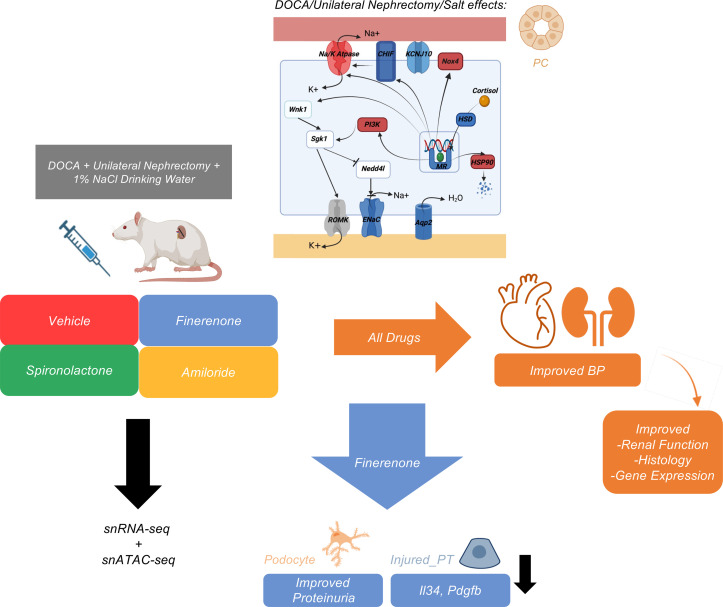

## Introduction

Chronic kidney disease (CKD) is a serious and growing public health issue, affecting approximately 14% of the population in the United States (1). It is the ninth leading cause of death globally and one of the fastest growing causes of mortality (2, 3). While medications including angiotensin-converting enzyme inhibitors (ACEIs), angiotensin receptor blockers (ARBs), and, more recently, sodium-glucose cotransporter 2 inhibitors have been shown to slow the progression of CKD (4–7), there is still a significant need for treatments and interventions to prevent this disease. There are currently half a million people in the United States with end-stage kidney disease (8).

Mineralocorticoids, such as aldosterone, play a key role in maintaining blood volume, sodium, and potassium balance (9). The primary mechanism by which aldosterone exerts its effects is through binding to the mineralocorticoid receptor (MR, *Nr3c2*). When aldosterone binds to MR in the cytosol, the complex travels to the nucleus, binds to DNA, and regulates gene transcription (10). While both aldosterone and cortisol can bind to the MR, in mineralocorticoid-sensitive tissues, an enzyme called 11β-hydroxysteroid dehydrogenase type 2 (11β-HSD2) degrades glucocorticoids, allowing the MR to be exclusively regulated by mineralocorticoids (11). The kidney is the primary target organ of mineralocorticoids (12).

Excess aldosterone can cause a range of health problems including hypertension (HTN), cardiovascular disease, and kidney disease (13, 14). The exact mechanisms by which aldosterone and HTN cause kidney damage are not fully understood (15). At high blood pressure, the afferent glomerular arteries must constrict to protect the glomerulus from high physical pressure, but glomerular vasoconstriction can lead to interstitial and tubular hypoxia, as the efferent artery plays a critical role in supplying blood to the tubules (16). In addition to the hemodynamic effects of mineralocorticoids, aldosterone may also directly regulate gene expression to induce kidney disease. The MR is expressed by various cell types in the body, including brain, heart, blood vessel, and immune cells (17). Some studies propose a direct role of MRs in regulating collagen accumulation and inflammation through the action of TGF-β and NF-κB (18, 19). There is also evidence that MR may directly affect immune cells, inducing the production of inflammatory cytokines such as IL-1 in macrophages (20).

Mineralocorticoid receptor antagonists (MRAs) are a group of medications that inhibit the effect of aldosterone (21). MRAs can be classified into 2 groups: steroidal MRAs, such as spironolactone, and nonsteroidal MRAs, such as finerenone (22). Spironolactone use is often associated with hyperkalemia and gynecomastia, which are probably in part caused by the nonspecific binding of spironolactone to the sex hormone receptors (23). In several small trials, spironolactone treatment on top of standard of care has resulted in blood pressure and proteinuria reduction in patients with CKD (21, 22). Unfortunately, in these studies the risk of potential life-threatening hyperkalemia was approximately 3-fold higher when compared with the use of ACEIs or ARBs alone.

Finerenone is a novel, highly specific nonsteroidal MRA (24). Finerenone demonstrated kidney and heart protection in animal models, but compared with steroidal MRAs, it had fewer effects on potassium homeostasis (24). Finerenone significantly reduced renal composite outcomes (kidney failure, a sustained decrease of the estimated glomerular filtration rate [eGFR] >40% from baseline or renal death) and cardiovascular outcomes (time to cardiovascular death, nonfatal MI, or stroke, or hospitalization for heart failure) on top of maximized ACEI or ARB treatment compared with placebo in at-risk patients (5). The incidence of hyperkalemia-related discontinuation of the drug was lower than that seen with spironolactone when used on top of ACEIs and ARBs (5). However, the exact molecular mechanism of finerenone action is not fully understood.

The kidney contains more than 30 different cell types, and bulk gene expression analysis can be strongly confounded by changes in cell fractions (25). Single-cell and single-nucleus RNA-Seq (scRNA-Seq and snRNA-Seq) enables the unbiased transcriptomic characterization of kidney cells (26–28) including cell-type–specific changes in disease state (29, 30). Single-nucleus profiling of chromatin accessibility, which is called the single-nucleus assay for transposase-accessible chromatin using sequencing (snATAC-Seq) has recently been developed to identify open chromatin regions in the genome where transcription factors can bind (26, 30). This is particularly important for transcription factors such as the MR, which can directly bind to DNA to regulate gene transcription.

In this study, we performed snRNA-Seq and snATAC-Seq to elucidate mineralocorticoid-induced HTN and the development of hypertensive kidney disease. We define deoxycorticosterone acetate–sensitive (DOCA-sensitive) cells and genes and the mechanism of finerenone-conferred kidney protection and made a direct comparison with changes induced by spironolactone and amiloride.

## Results

### Finerenone ameliorates cardiac and renal injury in the DOCA-salt rat injury model.

**Figure 1:**
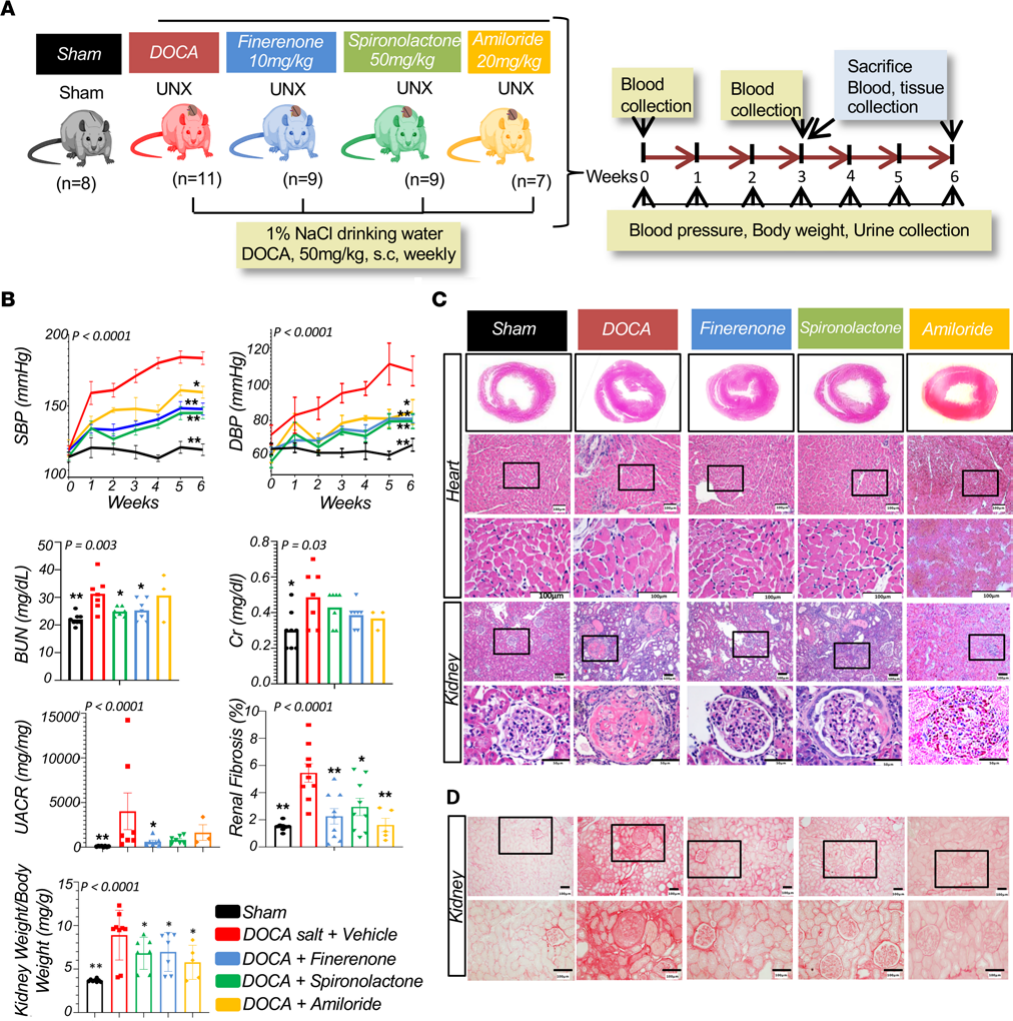
MRAs protect against DOCA-salt–induced cardiorenal damage. (**A**) Study overview. Rats were divided into 5 treatment groups: sham; DOCA plus vehicle; DOCA plus finerenone; DOCA plus spironolactone; and DOCA plus amiloride. UNX, uninephrectomized. (**B**) Clinical and biochemical parameters in the experimental rat groups, including SBP and DBP, BUN, creatinine (Cr), urinary albumin creatinine ratio (UACR), kidney-to-heart weight ratio, and renal fibrosis. The *x* axis shows the weeks following the surgery, and the *y* axis shows the measurement values. One-way ANOVA was used to compare all groups, and the 2-tailed Student’s *t* test was used to compare each group with the DOCA-salt group. For statistical comparison, log-transformed data were used to determine the UACR. *P < 0.05 and **P < 0.01, for differences in the parameters measured between sham-, finerenone-, spironolactone-, and amiloride-treated rats compared with DOCA-treated rats as follows: Error bars indicate the SEM. (**C**) Representative H&E-stained kidney and heart sections from animals in the experimental groups. Scale bars: 100 μm (heart); 100 μm (kidney) and 50 μm (kidney, enlarged insets). Original magnification, ×20. (**D**) Representative Picrosirius red staining of kidney sections from animals in the experimental groups. Scale bars: 100 μm. Original magnification, ×20.

We modeled mineralocorticoid-induced HTN and cardio-renal syndrome by performing uninephrectomy followed by DOCA injection and high-salt intake in rats. Groups of rats were treated with 10 mg/kg finerenone, 50 mg/kg spironolactone, or 20 mg/kg amiloride (24, 31–33). Two rats from each group were sacrificed after 3 weeks to evaluate short-term changes (HTN), whereas the majority were sacrificed at 6 weeks to assess hypertensive organ damage (Figure 1A). Rats treated with DOCA and given high amounts of salt in the drinking water (DOCA-salt) developed severe HTN, with 181 mmHg SBP compared with 115 mmHg SBP in sham control rats by the end of the study (Figure 1B). Finerenone, spironolactone, and amiloride resulted in a similar reduction in systolic blood pressure (SBP) and diastolic blood pressure (DPB) (Figure 1B). Serum blood urea nitrogen (BUN) levels were higher in the DOCA-salt group compared with controls, and these levels was lowered by finerenone and spironolactone treatment. Anemia, a common complication of CKD, was ameliorated by treatment with finerenone, spironolactone, and amiloride (Supplemental Figure 1A; supplemental material available online with this article; https://doi.org/10.1172/JCI157165DS1). DOCA-treated rats developed severe proteinuria, which was reduced by all treatments, but the reduction only reached statistical significance in the finerenone-treated group (*P = 0.04*) (Figure 1B). As the urinary albumin creatinine ratio (UACR) variance was high, the study was not powered to detect differences in other groups. Plasma renin levels were lower in the DOCA-treated rats when compared with controls but varied markedly (Supplemental Figure 1A).

We observed cardiac hypertrophy in DOCA-treated rats, which was probably secondary to the HTN. The heart-to-BW ratio was lower in the finerenone-, spironolactone-, and amiloride-treated rats, consistent with the drugs’ effect on blood pressure (Supplemental Figure 1A). The kidney-to-BW ratio was markedly increased in DOCA-treated rats but was reduced by treatment with finerenone, spironolactone, or amiloride (Figure 1B and Supplemental Figure 1A).

Histological analysis revealed severe global and segmental glomerulosclerosis, proteinaceous casts, tubulointerstitial fibrosis, and extensive cardiac fibrosis (Figure 1, C and D, and Supplemental Figure 1A). We performed Picrosirius red staining to measure the extent of fibrosis in kidneys and hearts (Supplemental Figure 1B). Rats treated with MRAs or amiloride showed less fibrosis and damage.

We analyzed several phenotypic outcomes, including blood pressure, biochemical parameters (such as proteinuria, BUN, blood electrolytes), and structural damage (such as glomerulosclerosis and fibrosis) in rats. To evaluate overall phenotypic similarities among the samples, we used unbiased principal component analysis (PCA) and hierarchical clustering of the outcomes (Supplemental Figure 2). The results showed that the DOCA-treated rats were distinct from the control rats, as well as those treated with finerenone, spironolactone, or amiloride. Control rats were distinct from rats in the finerenone-, spironolactone-, and amiloride-treated groups. Outcomes of the finerenone-, spironolactone-, and amiloride-treated groups were similar, indicating that HTN played a key role in the development of the phenotype.

In summary, we observed severe HTN and renal failure in the DOCA-salt rat model. MRA and epithelial sodium channel (ENaC) inhibition lowered blood pressure and similarly improved phenotypes, indicating a key role of HTN in driving cardiorenal damage. Within the same blood pressure range, finerenone was more successful in reducing proteinuria.

### Integrated single-cell multiomics atlas of the of healthy and diseased rat kidneys.

We sought to identify genes, cell types, pathways, and transcription factors affected by DOCA, HTN, hypertensive kidney disease, and the response to MRAs and amiloride. Therefore, we conducted single-cell multiomics analysis, which included snRNA-Seq, snATAC-Seq, and bulk RNA-Seq of kidneys from control and experimental groups at both time points (Supplemental Figure 3).

**Figure 2:**
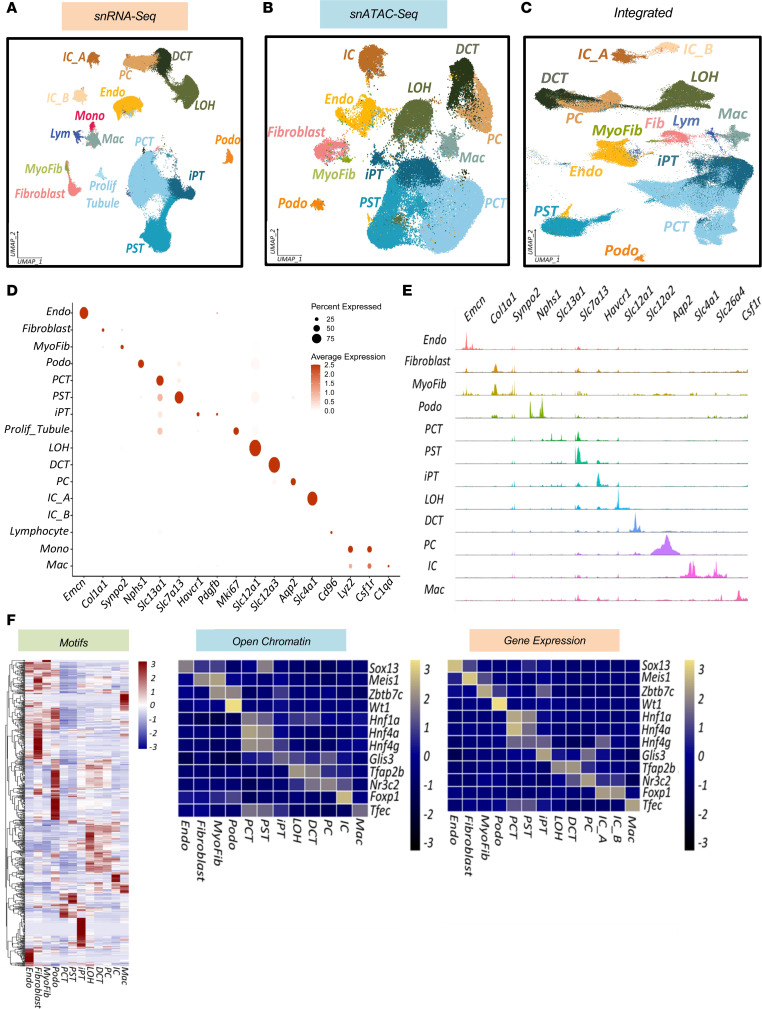
The single-cell multiomics landscape of healthy and diseased rat kidneys. (**A**) UMAP of 310,218 rat kidney snRNA-Seq data. (**B**) UMAP of 53,298 rat kidney snATAC-Seq data. (**C**) UMAP of integrated snRNA-Seq and snATAC-Seq of rat kidneys. (**D**) Bubble dot plots of marker genes used for cell-type annotation in the snRNA-Seq. The size of the dot indicates the percentage of positive cells, and the darkness of the color indicates the average expression. (**E**) Fragment coverage (frequency of Tn5 insertion) in each snATAC-Seq cluster at the cell-type marker gene promoter site. (**F**) Heatmap of average chromVAR motif activity for each cell type (far left panel). The color scale shows the *z* score scaled by row. Chromatin accessibility and gene expression of representative motifs of each cluster are shown in the middle and right panels, respectively. The color scheme of the heatmap is based on *z* score distribution. Each row represents a gene, and each column represents a cell type. Endo, endothelial cells; MyoFib, myofibroblasts; Podo, podocytes; Prolif_Tubule, proliferative tubule cells; Mono, monocytes; Mac, macrophages.

We conducted snRNA-Seq on 22 whole rat kidney samples from the 5 different groups. Quality control (QC) metrics, including gene counts, reads, and mitochondrial gene percentages, are shown in Supplemental Figure 4. DOCA administration, in combination with high-salt intake, has been reported to increase proximal tubular cell size (34). Therefore, we compared the mean RNA counts per cell and samples between the groups. We observed a trend toward higher count numbers in the DOCA-treated animals than in the controls, but this did not reach statistical significance (Supplemental Figure 4D). After filtering low-quality cells, a total of 310,218 cells were used in the final analysis. We identified 41 clusters after batch effect correction using Harmony (35) (Supplemental Figure 5). We next performed cell-type–specific differential expression analysis and identified all previously described kidney cell types (28, 36, 37) (Figure 2A and Supplemental Table 1). The key marker genes used to identify different cell clusters in the snRNA-Seq are shown in Figure 2D. As this is one of the first rat kidney snRNA-Seq data sets (38), we analyzed cell-type consistency (39) and specific gene markers in rat, mouse (28), and human (36, 37) kidneys. We observed that most cell-type–specific markers were conserved between the different species (Supplemental Figures 6 and 7)

To understand gene regulatory changes induced by DOCA and those influenced by MRAs, we performed snATAC-Seq on 9 samples. Supplemental Figures 8 and 9 show the QC parameters of the snATAC-Seq libraries. After filtering low-quality cells, clustering was performed on 53,298 nuclei. After batch-effect correction by Harmony (35), we identified 20 clusters (Supplemental Figure 10). We next examined chromatin accessibility around the transcription start site and gene body regions of the known cell-type–specific marker genes using Signac (40) (Figure 2B). Key marker genes showed cell-type–specific accessibility, and we were able to identify all major kidney cell types (Figure 2E). Chromatin accessibility–based marker gene activity is shown in Supplemental Figure 10E. Supplemental Tables 2 and 3 provide the full list of cluster-specific differentially accessible peaks and a list of gene activity calculated on the basis of differentially expressed genes (DEGs), respectively.

We performed label transfer to understand the relationship between cell-type–specific gene expression and open chromatin in the snRNA-Seq and snATAC-Seq data sets (41). We found strong consistency (0.78 mean of maximum prediction score) between these 2 data sets (Supplemental Figure 11, A–C, and Figure 2B). To further quantify the consistency between cluster assignment in the ATAC-Seq and RNA-Seq data, we extracted gene activities of the top 3,000 highly variable genes from snATAC-Seq and snRNA-Seq and ran a Pearson’s correlation test between gene expression (snRNA-Seq) and gene activity (snATAC-Seq) (Supplemental Figure 11D). Integration of the snRNA-Seq and snATAC-Seq data also yielded highly consistent results (Figure 2C and Supplemental Figure 12). The list of marker genes for the integrated data set can be found in Supplemental Figure 12B and Supplemental Table 4.

Overall, our snRNA-Seq, snATAC-Seq, and integrated data sets captured all kidney cell types, including endothelial cells, fibroblasts, myofibroblasts, podocytes, different types of proximal tubule (PT) cells, loop of Henle (LOH) cells, distal convoluted tubule (DCT) cells, principal cells of collecting ducts (PCs), intercalated cells (ICs), and diverse immune cells. Cell-type–specific markers were consistent with findings in prior publications for mouse (28) and human (27, 36, 37) kidneys such as *Emcn* for endothelial cells, *Col1a1* for fibroblasts, *Synpo2* for myofibroblasts, *Nphs1* for podocytes, *Mki67* for proliferating tubules, *Slc12a1* for LOH cells, *Slc12a3* for DCT cells, *Aqp2* for PCs, *Slc4a1* for type A ICs (IC_A), *Slc26a4* for type B ICs (IC_B), *Cd96* for lymphocytes, *Lyz2* for monocytes, and *Csf1r* and *C1qa* for macrophages. We captured different types of PT cells including proximal convoluted tubules (PCTs) and proximal straight tubules (PSTs) expressing *Cubn* and *Slc7a13*, respectively (Supplemental Figure 13). We also captured PT cells positive for *Havcr1, Vcam1*, and platelet-derived growth factor subunit b (*Pdgfb*), which are well-known markers of tubular injury. We labeled this cluster as an injured or profibrotic PT (iPT) cell cluster (Supplemental Figures 12 and 13).

To further understand key cell-type transcription factors in determining cellular gene expression, we used chromVAR to analyze motif activity and predicted key gene expression–driving transcription factors (42) in individual cell clusters (Figure 2F). This was based on the presence of binding motifs in the open chromatin area of each cell type. The complete list of differentially activated chromVAR motifs is shown in Supplemental Table 5. Our predicted gene expression–driving transcription factors in each cluster were consistent with prior reports, including *Hnf1a* and *Hnf4g* (26, 27) in PT cells and *Wt1* (26, 43) in podocytes, among others.

In summary, to our knowledge, we have generated one of the first comprehensive single-cell expression and gene-regulatory atlases for healthy and diseased rat kidneys, which is now available to the public on the Susztak laboratory website at: www.susztaklab.com (https://susztaklab.com/genemap_rat).

### MR target cell types and gene-regulatory network in the rat kidney.

**Figure 3:**
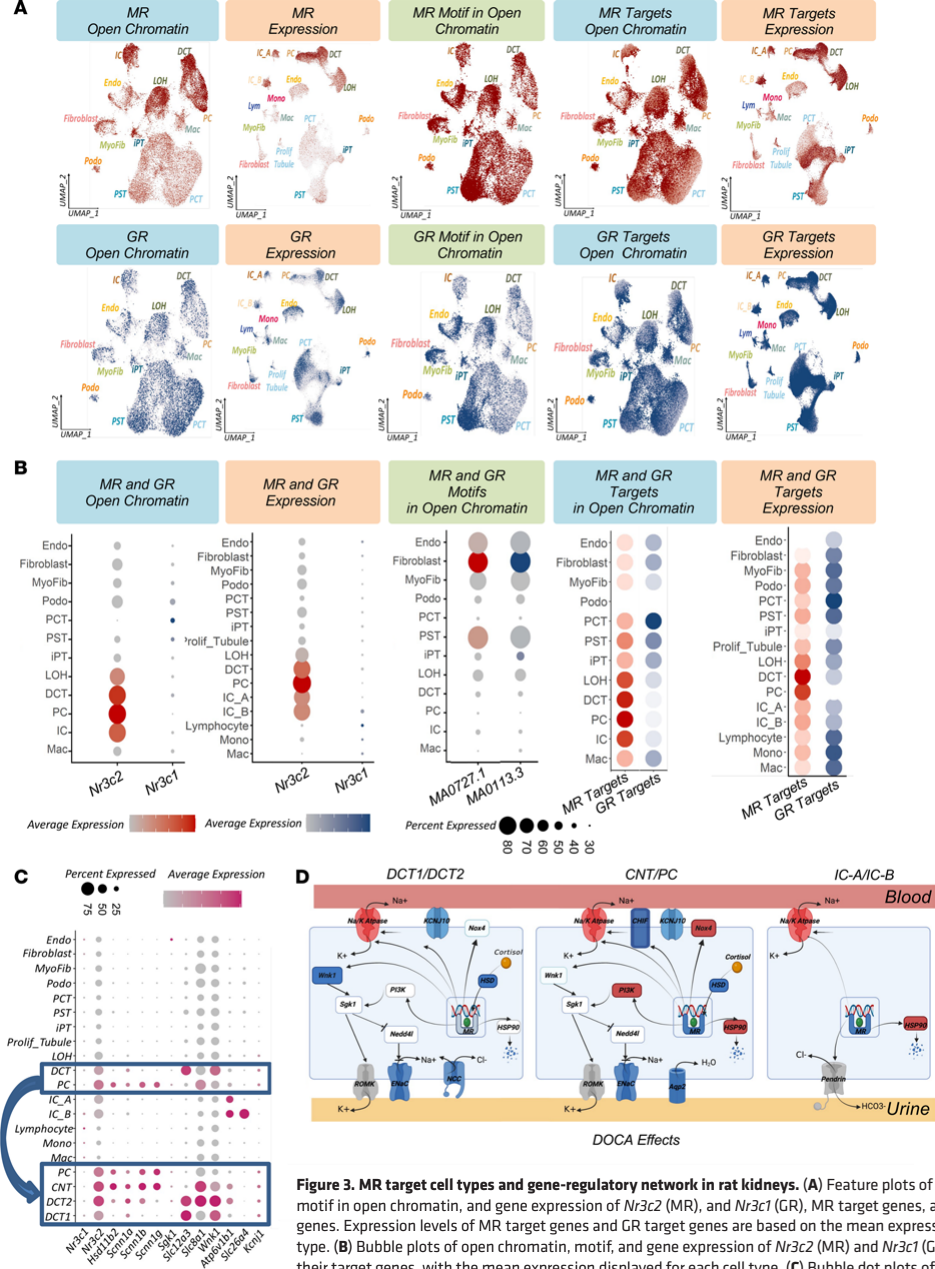
MR target cell types and gene-regulatory network in rat kidneys. (**A**) Feature plots of open chromatin, motif in open chromatin, and gene expression of *Nr3c2* (MR), and *Nr3c1* (GR), MR target genes, and GR target genes. Expression levels of MR target genes and GR target genes are based on the mean expression in each cell type. (**B**) Bubble plots of open chromatin, motif, and gene expression of *Nr3c2* (MR) and *Nr3c1* (GR), including their target genes, with the mean expression displayed for each cell type. (**C**) Bubble dot plots of mineralocorticoid target genes and the GR (Nr3c1) in the snRNA-Seq data set before and after subclustering of DCT and PC cells. The size of the dots indicates the percentage of positive cells, and the darkness of the color indicates average expression. (**D**) Schematic of MR target genes affected by DOCA in the DOCA-salt rat nephropathy model. Genes are colored blue (lower expression), red (higher expression), or white (unchanged expression). Notably, *Atp1a1* and *Atp1b1* showed increased expression, whereas *Hsd11b2* expression was lower in all cells. *Pik3r3* expression was higher in PC cells. ENaC genes (*Scnn1a*, *Scnn1b*, *Scnn1g*), *Wnk1*, and *Aqp2* showed decreased expression. ROMK, renal outer medullary potassium channel; Nox4, NADPH oxidase 4.

Our primary objective was to understand mineralocorticoid and glucocorticoid action; target genes and cell types. This has traditionally been a challenging task, as cells cultured in vitro demonstrate marked differences in their gene expression. First, we examined open chromatin regions at the MR (*Nr3c2*) and glucocorticoid receptor (GR, *Nr3c1*) loci in the entire data set. We observed accessible chromatin for the MR in several cell types, the highest being in PC cells, but also in DCT and IC cells. In contrast, GR open chromatin exhibited almost an inverse pattern, with a lack of open regions in the distal nephron, but accessibility in most other cell types (Figure 3A). These results suggest that the cell-type–specific expression of the GR and MR plays a key role in their target actions (44). MR expression, as measured in the snRNA-Seq data, was the highest in PC cells (Figure 3B) (45). GR expression was much lower than MR expression in the kidney and was expressed by multiple cell types with the exception of PC cells.

Next, we examined the activity of the MR (*Nr3c2*) motif in open chromatin areas within our rat kidney atlas. We found open chromatin for MR motifs in almost all cell types (Figure 3A). As the binding motif for *Nr3c2* and *Nr3c1* is the same, we observed no difference in open motif activity between the MR and the GR. We then investigated the open chromatin and gene expression of previously published MR and GR target genes (46–54) (Supplemental Table 6). The results showed a consistent pattern with MR and GR expression, respectively (Figure 3, B and C, and Supplemental Figure 14).

Further subclustering of DCT and PC cells, led us to identify the connecting tubule (CNT) and 2 subtypes of DCT cells (DCT1 and DCT2). DCT2 cells expressed *Slc12a3* (classic DCT marker) as well as the classic PC markers (ENaC) (55) (Figure 3C and Supplemental Figure 15). MR sensitivity was established by MR expression and by the expression of 11β-hydroxysteroid dehydrogenase type 2 (*Hsd11b2*), an enzyme that degrades glucocorticoids. In cells that do not express 11-β-HSD2, MR is effectively a glucocorticoid receptor. As expected, *Hsd11b2* expression was the highest in PC cells, however subclustering indicated expression in DCT2 and CNT cells. Expression of the classic MR target genes ENaC (*Scnn1a*, *Scnn1b*, *Scnn1g*) and *Sgk1* was the highest in PC cells, but CNT cells also expressed ENaC, while its expression in DCT2 cells was lower. In summary, the effect of the MR appeared to be controlled by multiple sequential mechanisms, including MR chromatin accessibility and cell-type expression of the MR, *HSD11B2* and MR target genes.

Next, we focused on genes upregulated by DOCA but downregulated by MRAs. We identified a number of MRA-sensitive genes in snRNA-Seq and bulk RNA-Seq (Supplemental Figures 16 and 17) (6-week data). In bulk RNA-Seq data, the expression of key MR target genes, including Na/K ATPase (*Atp1a1*) and PI3K (*Pik3r3*), was higher in DOCA-treated rats and returned to baseline levels after MRA treatment (Figure 3D and Supplemental Figure 17B). MRA-sensitive expression changes in PC cells were consistent with the bulk data and showed that, among other genes, the expression of *Atp1a1* was lower in finerenone-treated animals. A full list of MRA-sensitive genes in PC, CNT, and DCT2 cells is provided in Supplemental Table 7. Genes encoding the corticosteroid hormone–induced factor (CHIF), a negative regulator of Na/K ATPase (*Fxyd2* and *Fxyd4*), were expressed at lower levels in DOCA-treated rats. *Hsd11b2* expression was also lower in DOCA-treated rats and returned to baseline after treatment with finerenone (Supplemental Figures 16 and 17). *Aqp2* expression was also lower in DOCA-treated rats but was normalized by MRA treatment (Figure 3D and Supplemental Figures 16 and 17). The snATAC-Seq results were consistent with the snRNA-Seq results (Supplemental Figure 15D), and changes in Na/K/ATPase, ENaC, and PI3K levels were observable in the bulk RNA-Seq data as well.

In summary, unbiased snRNA-and ATAC-Seq analysis highlighted the fact that PC cells were the primary mineralocorticoid-sensitive cell type, with a minor role of DCT2 and CNT cells. Mineralocorticoid sensitivity was achieved by cell-type–selective gene expression and open chromatin.

### MRA and amiloride target genes, cell types and pathways.

**Figure 4:**
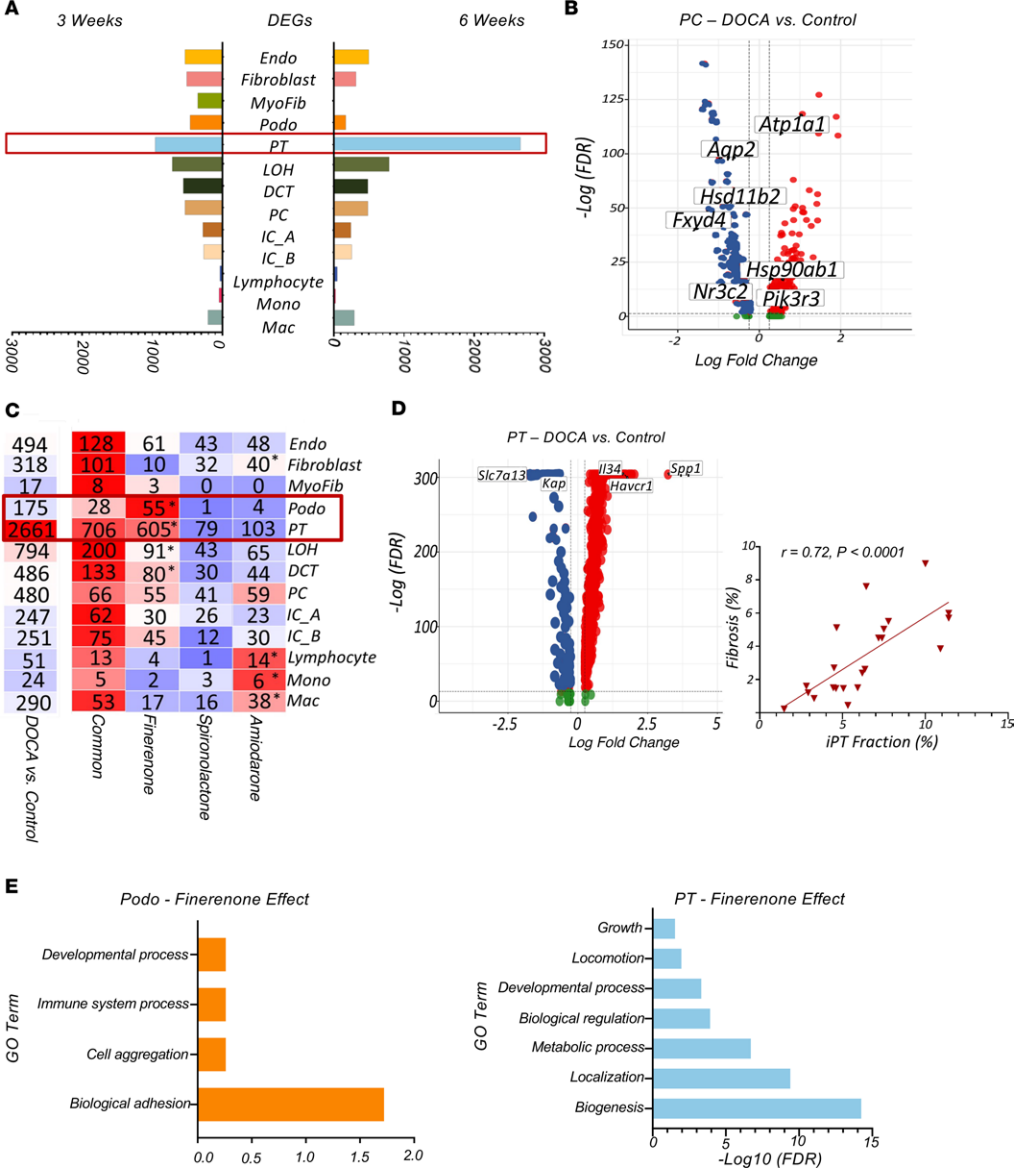
MRA and amiloride target genes, cell types, and pathways. (**A**) Number of DEGs between DOCA-treated and control groups in all kidney cell types 6 weeks and 3 weeks after DOCA administration. (**B**) Volcano plot of DEGs between the DOCA and control groups in PC cells at 6 weeks on DOCA. (**C**) Number of DEGs between control and DOCA groups in each cell type. The number of genes was normalized by all drugs or by the specific drugs finerenone, spironolactone, or amiloride. The color indicates a heatmap, more DEGs are in red, fewer in blue. Asterisks indicate significant DEG differences (normalized genes), calculated using the χ^2^ test (*P* < 0.05). (**D**) Upper panel shows a volcano plot of DEGs between the DOCA and control groups in PT cells at 6 weeks on DOCA. Lower panel shows the correlation between iPT fractions and renal fibrosis in all samples using Pearson’s correlation. (**E**) GO analysis of the genes affected by finerenone in podocytes and PT cells using DAVID. The enriched pathway is shown by the –log (FDR) of each pathway.

To investigate the specific cell types affected by MRAs and ENaC inhibitors and to differentiate between the effects of HTN and mineralocorticoids, we conducted cell-type–specific gene expression analysis of samples from control, DOCA-, and drug-treated groups, both at the time of HTN (3 weeks) and at the time of hypertensive kidney disease (6 weeks). Cells obtained from kidneys at the onset of HTN (3 weeks) showed comparable changes in gene expression levels across various cell types, suggesting that HTN led to significant functional changes in these cells. By contrast, PT cells from kidneys collected at the time of hypertensive kidney damage (6 weeks) showed much greater changes in gene expression (approximately 2,700 DEGs compared with ~200 DEGs in the other cell types) (Figure 4, A and B, and Supplemental Tables 7 and 9). This is congruent with previous research emphasizing the critical role of PT cell plasticity in the development of fibrosis (56, 57). These differences in gene expression were likely due to the presence in the kidneys of iPT cells that had hypertensive nephrosclerosis. These tubules formed a distinct cluster that expressed higher levels of *Havcr1*, secreted phosphoprotein 1 (*Spp1*), and *Il34* and lower levels of classic PT genes *Slc7a13* and *Kap* (Figure 4D). The proportion of iPT cells in the kidney correlated with the degree of fibrosis across samples, suggesting a robust link between iPT cells and fibrosis (Figure 4D). The same pattern was observed in the snATAC-Seq data (Supplemental Figure 18 and Supplemental Table 8).

To understand the specific changes in kidney cells that were brought about by different medications, we conducted gene expression analysis on each treatment group and cell type (at 6 weeks of treatment) (Figure 4, B–D). As shown in Figure 4C, the expression levels of most genes that were altered in the DOCA-treated rats reverted to baseline following treatment with MRAs or amiloride. This was likely due to the blood pressure–lowering effects of these medications (Figure 4C and Supplemental Figures 19 and 20). Amiloride exerted a marginally greater effect on immune cells and fibroblasts, although the absolute number of DEGs affected in these groups was minimal. Finerenone was found to be more effective in podocytes and PT cells, as more genes in these cell types returned to healthy expression levels following treatment. This was consistent with the observed clinical change in proteinuria (Figure 4C). In PT cells, the genes normalized by finerenone, including *Wfdc2*, *Ass1*, and *Assc3*, were mainly involved in metabolic processes. In podocytes, finerenone reduced the expression levels of *Tgfb2*, *Nfkbiz*, and *Fn1* that had been induced by DOCA, as well as the expression levels of genes associated with cell adhesion (Figure 4E). The complete list of genes affected by each drug is provided in Supplemental Table 10.

To summarize, we observed a robust correlation between PT and iPT genes and the development of HTN-induced fibrosis. All of the drugs tested were able to normalize the gene expression changes induced by DOCA, with finerenone being particularly effective in podocytes and PT cells.

### Unbiased tensor decomposition of a key phenotype driving cell types and pathways.

**Figure 5:**
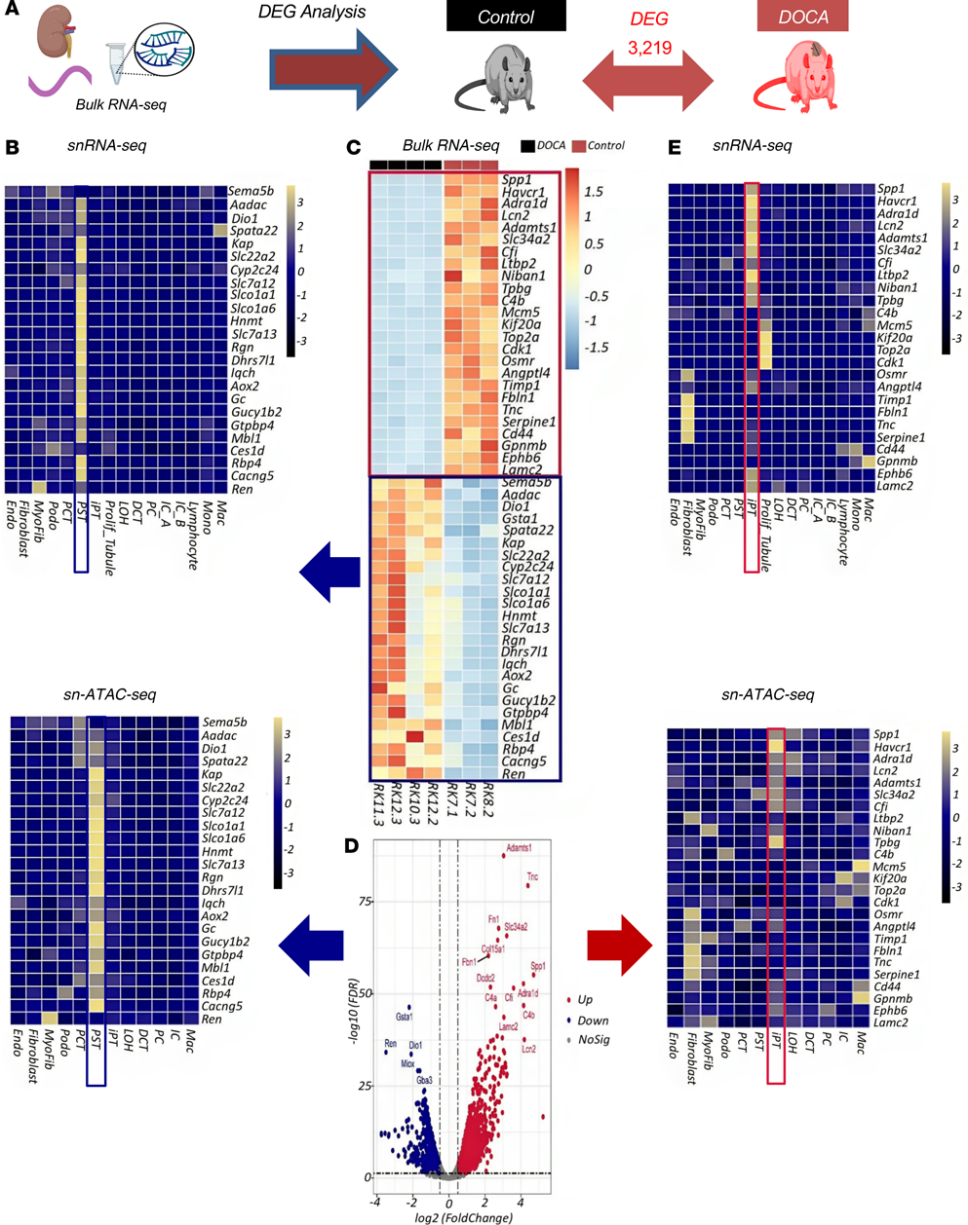
Genome-wide gene expression changes in whole-kidney samples from DOCA-treated rats given MRAs. (**A**) Number of DEGs between DOCA-treated and control groups by bulk RNA-Seq analysis. (**B**) Enrichment of genes showing lower expression with DOCA in PST cells in the snRNA-Seq and snATAC-Seq data sets. The color scheme of the heatmap is based on *z* score distribution. Each row represents a gene, and each column represents a cell type. Yellow indicates cell-type–enriched genes. (**C**) Expression of 25 genes showing higher or lower expression levels in DOCA versus control groups in the bulk RNA-Seq data set. The color scheme of the heatmap is based on *z* score distribution. Each row represents a gene, and each column represents a rat sample. Black and red colors indicate control and DOCA-treated rats, respectively. (**D**) Volcano plot of DEGs between DOCA and control groups in the bulk RNA-Seq data. (**E**) Cell-type expression (snRNA-Seq and snATAC-Seq) of the top upregulated DEGs in DOCA versus control groups identified in the bulk analysis. The color scheme of the heatmap is based on *z* score distribution; yellow indicates higher expression, while blue indicates lower expression. Each row represents a gene, and each column represents a cell type.

To identify cell types that drive phenotypic changes, we first generated bulk RNA-Seq data from whole-kidney tissue samples (Supplemental Figure 21) (6-week treatment data). DEG analysis revealed changes in the expression levels of 3,219 genes in kidneys between control and DOCA-treated rats (Figure 5A and Supplemental Table 11). Gene ontology (GO) pathway analysis using the Database for Annotation, Visualization, and Integrated Discovery (DAVID) (58) indicated enrichment for inflammation and metabolic processes in the DOCA-treated group (Supplemental Figure 21, B and C). Clustering analysis of the gene expression matrix of the bulk RNA-Seq indicated coclustering in the control and finerenone treatment groups (Supplemental Figure 21D). We subsequently examined expression levels of the DEGs identified by bulk RNA-Seq analysis in different cell types in the entire snRNA-Seq and snATAC-Seq data sets. We found that genes expressed at lower levels in DOCA samples were enriched in the proximal straight tubule (PST) segment (Figure 5, B–D), whereas genes with higher expression in DOCA samples were enriched in the iPT cluster (Figure 5, C–E). Overall, DEGs from bulk RNA-Seq were enriched in PT cells, which was consistent in the results from the snRNA-Seq and snATAC-Seq (Supplemental Figure 22). Weighted coexpression network analysis (WGCNA) (59) of the bulk RNA-Seq data identified various modules that correlated with different phenotypic outcomes, such as SBP, DBP, and fibrosis (Supplemental Figure 22). The primary module driving the phenotype in the DOCA models was enriched in PST cells. Genes whose expression returned to baseline in the finerenone-treated group were also enriched in PST and iPT cells, potentially indicating the key role of these cell types (Supplemental Figure 23).

**Figure 6:**
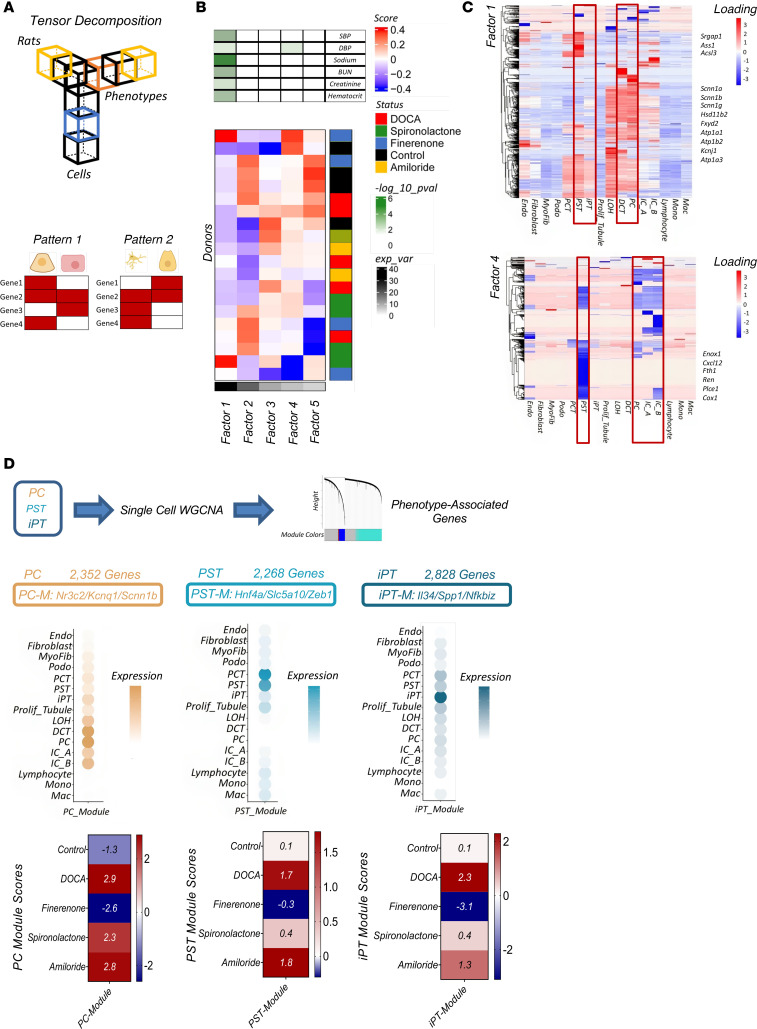
Principal cells and PT cells are the main target of mineralocorticoids and MRAs. (**A**) Overview of the tensor decomposition analysis. All rat samples were included in the analysis. (**B**) Sample score heatmap for decomposition of the snRNA-Seq data showing each sample and its loading score for each factor (lower panel). Colors on the right indicate sample groups. Colors in the bottom of the heatmap indicate each identified factor and its association with phenotypes. Each row represents a phenotype, and the color indicates the *P* value for the factor and phenotype association using univariate linear model *F* tests (upper panel). Two samples were filtered by the analysis. (**C**) Heatmaps showing the cell type gene loading scores of genes in factors 1 and 4. Some of the genes are highlighted. (**D**) Single-nucleus WGCNA of PC, PST, and iPT cells identified gene expression modules in cell types (top panel). The top representative genes in each cell-type–specific module are highlighted. Bubble plots indicate gene expression levels of the genes in each module in each cell type (middle panel) calculated by the average expression of all genes in a module in a specific cell type. The lower panel shows the module scores per condition for each cell type.

Given the complex experimental design, with multiple treatment groups, cell types, and phenotypic outcomes, we used tensor decomposition to identify the key cell types, genes, and pathways that were driving these phenotypes (on the entire snRNA-Seq data set) (60) (Figure 6A). In this analysis, we identified 5 factors (Figure 6B). The first factor was found to be associated with multiple important phenotypic outcomes, including blood pressure, serum sodium, BUN, and creatinine. Changes in expression of MR target genes, including *Scnn1a*, *Scnn1b*, *Scnn1g*, and *Atp1a1* and DCT and PC, as well as genes expressed in DCT and PC cells, were found to be driving this factor (Figure 6C). Interestingly, PST and iPT cells also exhibited enrichment in this factor (Figure 6C). Factor 4 was correlated with DBP and included the genes such as *Ren*, *Cox1*, *Plce1*, and *Cxcl12*. Most variations in this factor were observed in the DOCA treatment group, and it was enriched in PC cells, although we again noted an effect in PST cells (Figure 6C). The genes identified in factors 1 and 4 by this analysis are presented in Supplemental Table 12. In summary, tensor decomposition indicated PST, iPT, and PC cells as key phenotype-driving cell types.

After identifying key phenotype-associated cell types, we performed unbiased machine-learning gene expression analysis using WGCNA on PC, PST, and iPT cells (in the entire data set) (59, 61, 62) (Figure 6D). We found a specific gene module in PC cells containing the MR and its target genes. This module had the highest score in the DOCA group and amiloride group, and the lowest in the finerenone group. We identified a specific gene module in PST cells. This module expressed typical PST cell markers such as *Hnf4a* and *Slc5a12* as well as *Zeb1*, a marker for epithelial-mesenchymal transition. This module had the highest score in DOCA and was normalized best by finerenone. Furthermore, we identified an iPT-specific module, which showed enrichment for inflammatory markers such as *Il34*, *Spp1*, and *Nfkbiz*. This module had the highest score in DOCA and was normalized by finerenone. The complete list of the genes in these 3 identified modules is presented in Supplemental Table 13.

Overall, the bulk RNA-Seq, snRNA-Seq, and snATAC-Seq data suggested a critical association between PC and PT cells as the primary cell types driving phenotypes. WGCNA identified a core MR target network in PC cells and a proinflammatory profibrotic gene module in iPT cells.

### Finerenone protects against maladaptive differentiation of iPT cells.

**Figure 7:**
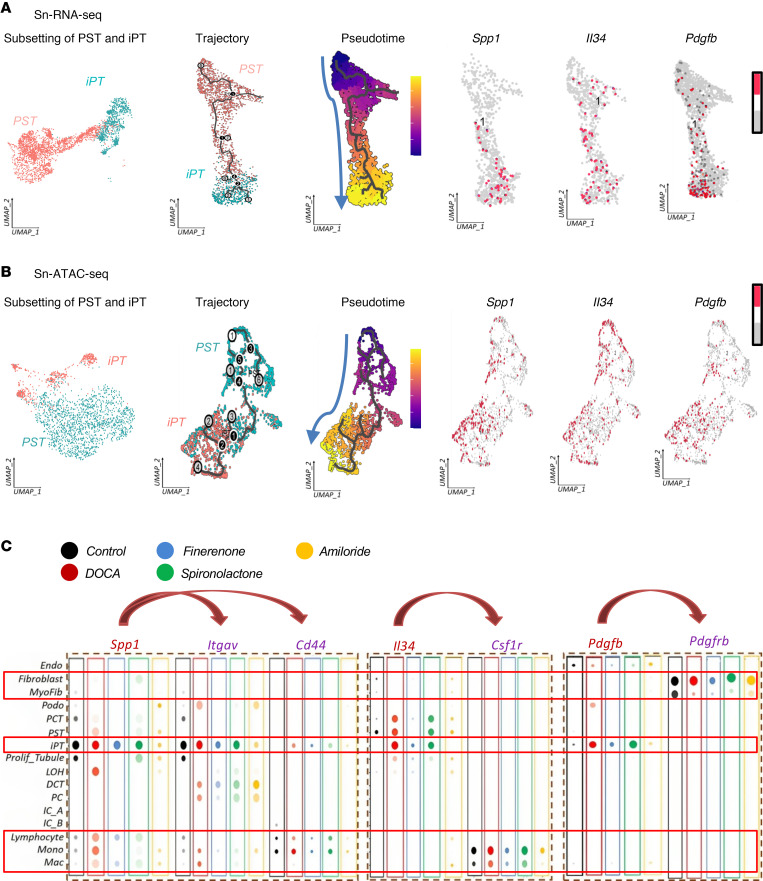
Cellular trajectory of PST and iPT cells highlights *Spp1,* *Il34*, and *Pdgfb*. (**A**) (Left panel) UMAP representations of PT cell subclustering and iPT cell differentiation trajectory from PST cells in snRNA-Seq. Cells are colored according to pseudotime, and the arrow indicates the direction of the pseudotime. (Right panel) UMAP representations of gene expression of *Spp1*, *Il34*, and *Pdgfb* during trajectory (red dots indicate the expression of each gene in the cells). (**B**) (Left panel) UMAP representations of PT cell subclustering and iPT cell differentiation trajectory from PST cells in snATAC-Seq. Cells are colored according to pseudotime, and the arrow indicates the direction of the pseudotime. (Right panel) UMAP representations of gene activity of *Spp1*, *Il34*, and *Pdgfb* during trajectory (red dots indicate the gene activity calculated on the basis of chromatin accessibility). (**C**) Bubble dot plot of the expression of *Spp1*, *Il34*, and *Pdgfb* genes and their receptors *Itgav*, *Cd44*, *Csf1*, and *Pdgfrb* in different cell types and groups. The size of the dot indicates the percentage of positive cells, and the darkness of the color indicates average expression.

Our bulk RNA-Seq and scRNA-Seq analyses revealed a key association between PT cells and hypertensive organ damage. To identify factors that might be responsible for the maldifferentiation of iPT cells, we used Monocle3 to conduct a cell trajectory analysis (63, 64) (Figure 7). This analysis identified a path of differentiation of iPT cells from PST cells (Figure 7A). iPT cells were enriched in the diseased state (27) (Supplemental Figure 24). GO analysis revealed an enrichment of genes associated with metabolism and development along the trajectory (Supplemental Figure 24 and Supplemental Table 14). We identified *Spp1*, *Il34*, and *Pdgfb* as top DEGs, showing higher levels in the iPT pseudotime trajectory (Figure 7A and Supplemental Figure 24B) and *Hnf4a*, which showed higher expression at the start of the trajectory (PST cells) and lower expression in iPT cells (at the end) (Supplemental Figure 24). Our snATAC-Seq data recapitulated changes observed with snRNA-Seq (Figure 7B) including changes in metabolic and developmental genes along the trajectory (Supplemental Figure 24 and Supplemental Table 15). Similar to the snRNA-Seq, chromatin accessibility at *Spp1* and *Il34* increased at the end of the trajectory (iPT cells), indicating that chromatin remodeling might be responsible for the gene expression changes (Supplemental Figure 24D). To identify transcription factors that drive expression of *Spp1*, *Il34*, and *Pdgfb*, we performed single-cell regulatory network inference and clustering (SCENIC) (65) analysis and identified *Bcl3*, *Runx1*, *Fosl2*, and *Cebpz* as likely driver transcription factors (Supplemental Figure 25).

Bulk gene expression data showed consistent changes in *Spp1* and *Il34* (Supplemental Figure 24E). ISH further confirmed *Spp1* and *Il34* expression in PT cells in the DOCA group and lower expression levels in the control group (Supplemental Figure 26). Additionally, in spatial transcriptomics data obtained from patients with CKD, we were able to validate the coexpression of *HAVCR1*, *VCAM1*, *SPP1*, and *IL34* as the markers for iPT cells in regions with a high extracellular matrix (ECM) production score (fibrosis) (37) (Supplemental Figure 27).

Both *Spp1* and *Il34* are secreted molecules. To understand their effects on other cell types, we used CellChat to conduct ligand-receptor interaction analysis (66, 67) (Figure 7C and Supplemental Figure 28). The receptors for *Spp1,* *Itgav*, and *Cd44* were expressed by immune cells (Figure 7C). The predicted interaction between *Spp1* and its receptors was weak in the control and finerenone-treated groups but much stronger in the DOCA- and spironolactone-treated groups (Figure 7C and Supplemental Figure 28). We observed a similar pattern for *Il34* and its receptor *Csf1r* (Figure 7C and Supplemental Figure 28). This cell-cell communication analysis revealed intense interactions emanating from iPT cells, which were markedly lower in the finerenone group (Supplemental Figure 29). *Pdgfb*, a profibrotic factor, was highly expressed by iPT cells in DOCA samples and was found to interact with its receptor in fibroblasts (Figure 7C). Genes involved in ECM production and fibrosis, such as *Col1a1*, *Col3a1*, and *Acta2*, were expressed at the highest level in the DOCA-treated group and at lower levels in the finerenone-treated group (Supplemental Figure 30).

In summary we identified maladaptive differentiation of PT cells in a rat model of hypertensive kidney disease as well as changes in the expression of proinflammatory molecules that occurred during maldifferentiation.

### Conserved changes in human kidney disease samples.

Finally, we sought to determine the relevance of gene expression changes observed in iPT cells in the DOCA rat model to human kidney disease. To do this, we analyzed 991 microdissected human kidney tubule samples from healthy individuals and patients with varying degrees of diabetic and hypertensive kidney disease. The demographics and clinical and histological characteristics of the human kidney samples are shown in Supplemental Table 16.

**Figure 8:**
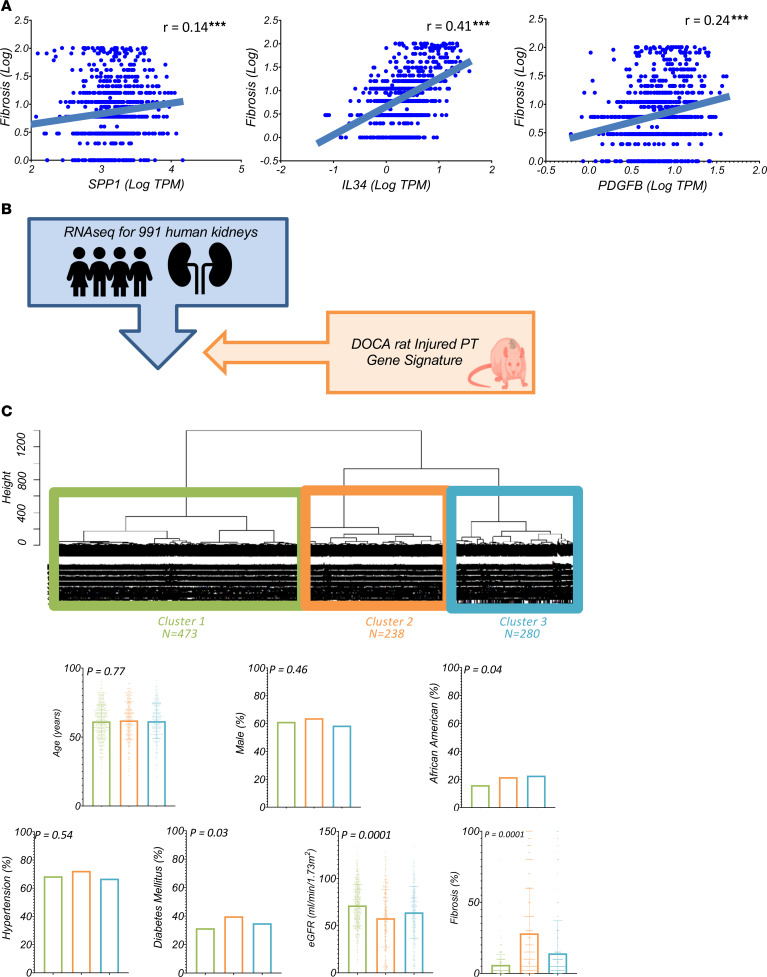
iPT cell signature can classify disease severity in human diabetic and hypertensive kidney tissue samples. (**A**) Correlations with fibrosis between *SPP1*, *IL34*, and *PDGFB* in microdissected human kidney tubule samples. The *x* axis represents normalized (log transcripts per million [TPM]) gene expression, and the *y* axis represents the fibrosis score (log-transformed). Each dot indicates 1 sample. Spearman’s test and correlation coefficient (*r*) as well as the regression line are shown in each plot. *P < 0.05, **P < 0.01, and ***P < 0.0001 (**B**) Schematic overview of the experiments. The homologous genes for the iPT gene signature in rats were used to cluster 991 human kidney microdissected tubules. (**C**) The 3 distinct human kidney clusters were identified on the basis of the iPT signature using hierarchical clustering. The 3 main clusters in the dendrogram are shown in different colors. Graphs represent the clinical information on samples from the 3 clusters. The χ^2^ test for nonparametric and 1-way ANOVA for parametric data were used for statistical comparisons. Error bars indicate the SD.

The expressions levels of *SPP1*, *IL34*, and *PDGFB* were found to positively correlate with fibrosis (Figure 8A). We then used the iPT gene signature to perform hierarchical clustering of the human kidney samples (Figure 8, B and C). This signature clustered the 991 human kidney samples into 3 subgroups. Although the clinical information was not used for the clustering, the samples in these groups showed differences in their clinical characteristics. For instance, samples in cluster 2 had the lowest eGFR and the highest degree of interstitial fibrosis. In this cluster, the expression levels of *SPP1*, *IL34*, and *PDGFB* were the highest compared with levels in the 2 other clusters, which exhibited better kidney function (Supplemental Figure 31).

Overall, the large human kidney data set from patients with hypertensive and diabetic kidney disease demonstrated changes consistent with those observed in the rat kidney data set. Additionally, the iPT signature, which includes *SPP1*, *IL34*, and *PDGFB*, was capable of identifying diseased human kidney samples.

## Discussion

Mineralocorticoids are hormones that play important roles in the conservation of sodium in land animals, which is a vital process for maintaining blood volume in environments where salt is scarce (68). Mineralocorticoid excess can cause HTN as well as kidney and heart disease (69). MRAs have been shown to protect against kidney and heart disease, however, the exact mechanism of action is poorly understood. In this study we used single-nucleus transcriptomics and open chromatin atlasing to examine the effects of mineralocorticoids in the kidneys of rats and analyzed the effects of 2 different MRAs and a direct blocker of ENaC side by side. Our omics data, alongside various bioinformatics analyses, enabled us to identify the primary cell types targeted by mineralocorticoids, as well as the cell types indirectly affected by these hormones and the target genes and networks involved. Our results demonstrate the central role of changes in PC cells in HTN and mineralocorticoid action and the importance of PST and iPT cells in driving hypertensive kidney fibrosis in the DOCA/uninephrectomy/high-salt intake rat model. Moreover, the iPT gene signature allowed us to classify kidneys from patients with hypertensive and diabetic kidney disease.

HTN is the most common chronic disease (70), affecting nearly half of the population in the United States (70). Hypertensive kidney disease is responsible for close to 25% of all cases of CKD and end-stage renal disease (ESRD) (8), but the underlying disease mechanisms are not fully understood. Recent clinical reports indicate the important role of mineralocorticoid excess in the development of HTN and CKD (12, 71). In this study, we performed detailed phenotypic and gene expression and gene regulation analyses at the bulk and single-cell levels in a rat model of mineralocorticoid-induced HTN and hypertensive end-organ damage (72). We also compared our results with those obtained from patients’ kidney samples.

We present what we believe to be one of the first kidney single-nucleus transcriptomics and chromatin accessibility data sets for rat kidneys. Our results demonstrate that cell types and cell-type–specific gene expression changes were mostly conserved between mouse (28), human (37), and rat kidneys, although further studies are needed to explore species-specific differences. Using gene expression and chromatin accessibility information, we confirmed the mineralocorticoid-sensitive segment of the distal nephron, which includes PC cells as well as CNT and DCT2 cells (73). We also identified the MR target gene network, which will be important for understanding the actions of mineralocorticoid in the future (74, 75). Our findings show that mineralocorticoid sensitivity in the kidney was established through multiple sequential mechanisms, including changes in chromatin openness and the expression of the MR, *Hsd11b2*, and MR target genes. We also observed that expression of the GR in the kidney was lower and followed an almost inverse pattern compared with that of MR expression.

Our work demonstrates the utility of single-cell expression analysis for the understanding of disease-driving pathways, the identification of drug targets, and the assessment of molecular outcomes for drug effectiveness. As clinical interventional studies are often costly, these intermediate molecular analyses could pave the way for novel clinical trial designs (76). We compared the effectiveness of spironolactone, finerenone, and a direct ENaC inhibitor in this study. All of these drugs effectively lowered blood pressure and protected against kidney damage. When we analyzed gene expression changes at the single-cell level, we found that these drugs effectively normalized DOCA-induced gene expression changes. Our data highlight the importance of blood pressure–driven changes in kidney-specific genes and the usefulness of single-cell gene expression in detecting such molecular outcomes. We also observed that finerenone was more effective in reducing proteinuria for the same blood pressure change and had consistent effectiveness on podocytes and PT cells.

In this study, we used various orthogonal bioinformatics approaches to identify the cell types and gene expression changes driving disease development. Our results showed that HTN was associated with changes in gene expression in multiple cell types. At the time of hypertensive nephrosclerosis, we observed that changes in PT cells were particularly important in driving the development of the disease phenotype. This was evident at both the bulk gene expression and single-cell levels. Unbiased tensor decomposition analysis revealed the crucial role of PC cells, as well as PT cells, especially iPT cells, in driving the development of the disease phenotype. The presence of iPT cells strongly correlated with fibrosis in all analyzed samples. Our analysis also identified *Spp1*, *Il34*, and *Pdgfb* as some of the top genes that showed differences in both the bulk and single-cell data sets. Their expression levels correlated with disease severity and improved following treatment with finerenone.

SPP1 is a phosphoprotein produced by the kidney that plays a role in cell adhesion and migration (77). Prior studies have shown the causal role of SPP1 in kidney disease in the unilateral ureteral obstruction (UUO), diabetic, and LPS-induced kidney disease models (77, 78). Recently, *SPP1* was proposed to be a hub gene for diabetic kidney disease progression by microarray analysis (79). Here, we show that *Spp1* was one of the key genes along the PST-to-iPT differentiation trajectory. *Spp1* is influenced by finerenone. The receptors of *Spp1*, such as *Cd44* and *Itgav*, were expressed by stromal and immune cells, and finerenone altered this interaction.

*IL34* is another gene we found to be correlated with PST-to-iPT differentiation and influenced by finerenone. IL-34 is a key cytokine that stimulates the influx of macrophages and monocytes via binding to its receptor colony-stimulating factor receptor 1 (CSF1R) (80). Prior studies have established the key role of PT-derived IL-34 and macrophage influx in the acute kidney injury and UUO models of kidney disease (80, 81). We demonstrated that *Il34* was highly expressed in DOCA-treated rats, especially in iPT cells, and that its expression was lower in finerenone-treated animals. PDGFB is a well-known secreted molecule involved in the activation of fibroblasts and has been shown to play a role in kidney fibrosis in mice (57, 82). We observed high expression of *PDGFB* in our iPT and profibrotic PT cells as well as high expression of its receptor *PDGFRB* in stromal cells, which could contribute to kidney fibrosis in hypertensive kidney disease. We also observed high production of ECM in the DOCA-treated group.

In this study, we found that changes in a rat model of hypertensive kidney disease, including the expression of iPT cells, *SPP1*, *IL34*, and *PDGFB*, strongly correlated with renal fibrosis in human kidney samples obtained from patients with hypertensive and diabetic kidney disease.

There are several limitations to our study. Although we incorporated earlier DOCA time points in our analysis to investigate a stage less affected by long-term HTN effects, it can be challenging to distinguish between gene expression changes related to direct DOCA-MR effects and secondary effects due to HTN. Additionally, our rat model incorporated 3 interventions: DOCA, unilateral nephrectomy, and salt intake, which collectively induce HTN. Therefore, the data not only reflect DOCA-induced changes but also changes in the response to uninephrectomy and high-salt intake. Most important, the observed changes were more consistent with the long-term HTN and DOCA effect rather than the acute and short-term activation of the MR in the kidney. Although we used a multiomics approach to evaluate the mechanisms of action of MRAs, computational analysis can be challenging, and some findings necessitate further experimental validation. Some cell types are difficult to capture in snRNA-Seq or snATAC-Seq, which limited our ability to identify MR targets. We used a rat model and supraphysiological doses of DOCA (an aldosterone precursor), which were likely higher than aldosterone concentrations in patients with CKD. Additional validation using samples from patients will be an important next step.

In summary, we have provided expression and chromatin accessibility maps for the rat kidney, defined MR target genes, cell networks, and the effects of steroidal and nonsteroidal MRAs as well as ENaC inhibition, and validated these findings in patient samples. Our study has delineated the mechanisms of MRA-mediated kidney protection at a cellular and molecular level.

## Methods

### Animals.

Male Sprague-Dawley rats weighing 250–300 g were obtained from Charles River Laboratories. Animals were housed in an environment with controlled temperature and humidity, a light/dark cycle at 22°C–24°C, and access to water and food ad libitum.

### DOCA-salt HTN model.

Rats were randomly divided into 5 groups. Group 1 underwent sham operation, whereas rats in groups 2–4 were uninephrectomized. Rats were anesthetized under isoflurane and buprenorphine (0.03 mg/kg) and subjected to left nephrectomy. One week after the operation, the rats in groups 2–5 received weekly s.c. injections of DOCA (50 mg/kg; MilliporeSigma) suspended in sesame oil and were supplied 1% NaCl in the drinking water. Daily gavage was performed, with group 2 receiving vehicle (10% ethanol, 40% solutol, 50% water); group 3 receiving finerenone (10 mg/kg/d); group 4 receiving spironolactone (50 mg/kg/d, MilliporeSigma); and group 5 receiving amiloride (20 mg/kg/d). To evaluate the early effects of DOCA and HTN, 2 rats from each group were sacrificed 3 weeks after the start of the intervention, with the remaining rats sacrificed at the conclusion of the 6-week period following the initiation of DOCA injections. Sham-treated animals were subjected to the same surgical procedure, in which the kidney was exposed but not removed, and received sesame oil injections and tap water.

### Blood pressure measurement.

SBP and DBP were measured in conscious animals using the standard tail-cuff method (Blood Pressure Analysis System, BP2000 Series, Visitech). All measurements were performed between 9:00 am and 1:00 pm, after 3–5 days of training.

### Plasma and urine sample analyses.

Blood samples were analyzed using the Abbott i-STAT portable clinical analyzer (Abbott Point-of-Care) and iSTAT 8+ cartridges (Abbott Laboratories) to determine sodium (Na), potassium (K), chloride (Cl), TCO_2_, anion gap, ionized calcium (iCa), BUN, creatinine, glucose (Glu), hematocrit (Hct), and hemoglobin (Hb).

Urine albumin was determined using a rat albumin–specific ELISA (Bethyl Laboratories), and creatinine was determined by reagent set (Diazyme, DZ072b-KY1), per the manufacturers’ protocols. The plasma renin sample was measured using ELISA (MilliporeSigma, RAB1162-1KT) according to the manufacturer’s protocol.

### Histological analysis.

For histological analysis, kidneys and hearts were fixed in 4% paraformaldehyde overnight, and then dehydrated, embedded into paraffin blocks, and sectioned onto glass slides. Sections were stained with H&E or Picrosirius red.

### ISH.

ISH was performed using formalin-fixed, paraffin-embedded kidney tissue samples and the RNAscope 2.5 HD Duplex Detection Kit (Bio-Techne, 322430) following the manufacturer’s original protocol. The following probes were used for the RNAscope assay: Rn-Il34-C2 (catalog 1056011-C2), Rn-Spp1-C2 (catalog 405441-C2), and Rn-Havcr1 (catalog 519081).

### snRNA-Seq and ATAC-Seq.

A detailed description of the snRNA and snATAC protocols can be found in the Supplemental Methods (83).

### Rat kidney bulk RNA-Seq.

Total RNA from 10 mg of each frozen rat kidney tissue sample was isolated using the Qiagen RNeasy kit (catalog 74106) according to the manufacturer’s instructions. To check RNA quality, the Agilent Bioanalyzer RNA 6000 Pico kit (Agilent Technologies, 5067-1513) was used. All samples with an RNA integrity number (RIN) of at least 6 were used for cDNA preparation. Strand-specific RNA-Seq libraries were generated using the NEBNext Ultra RNA Library Prep Kit for Illumina (catalog E7530L) following the manufacturer’s protocol, and the RNA-Seq libraries were sequenced to a depth of 20 million 2 × 150 paired-end reads.

### Human sample procurement.

Part of the collected tissues was formalin fixed and paraffin embedded and then sectioned and stained with periodic acid–Schiff, followed by scoring by a local renal pathologist. Human kidney tissues were microdissected under a dissecting microscope. RNA isolation, quality control, and the cDNA library for human microdissected tubules were performed as described (Supplemental Methods) for rat bulk RNA-Seq.

### Bioinformatics analysis.

A detailed summary of the bioinformatics analysis and applied computational tools can be found in the Supplemental Methods (83–89).

### Statistics.

Data are expressed as the mean ± SEM. One-way ANOVA was used to compare the continuous parameters between more than 2 groups. The 2-tailed Student’s *t* test was used to compare each group with the DOCA treatment group. The DOCA treatment group was the reference in by-comparison analyses. UACR values were log transformed before comparisons between groups. The χ^2^ test was used to compare the fractions and frequencies between groups. The correlation analysis between gene expressions and clinical characteristics in human samples was done using Spearman’s test. A *P* value of less than 0.05 was considered significant. Data are presented as the mean ± SEM.

### Study approval.

For the rat study, the protocol was approved by the IACUC of the University of Pennsylvania. The collection of human kidney tissue was approved by the IRB of the University of Pennsylvania. The study was IRB exempt because the samples were considered discarded medical materials, and all samples were permanently deidentified, thus no personal identifiers were collected.

### Data availability.

Raw data, processed data, and metadata from the snRNA-Seq, snATAC-Seq, and bulk RNA-Seq of different experimental groups of rats have been deposited in the NCBI’s Gene Expression Omnibus (GEO) database (GEO GSE183842). The human kidney RNA-Seq data are available in the GEO database (GEO GSE115098 and GSE173343). All the data supporting the graphs and tables are provided in the Supplemental Supporting Data Values file. The snRNA-Seq and snATAC-Seq data are available in the Susztaklab Kidney Biobank at susztaklab.com (https://susztaklab.com/genemap_rat) (see above GSE).

### Code availability.

All the codes used for the analysis were deposited inn GitHub (https://github.com/amin1990ab/DOCA_Rat_Kidney.git).

## Author contributions

ASN, JW, ZM, AA, and JF performed experiments and analyzed data. BP, TD, and XL performed experiments. AA, ZM, MSB, HL, SV, HC, KAK, and PK performed computational analysis. HC and PK offered suggestions for experiments. KS was responsible for the overall design and supervision of the experiments. AA and KS wrote the original draft of the manuscript. All authors contributed to and approved the final version of the manuscript.

## Supplementary Material

Supplemental data

Supplemental table 1

Supplemental table 2

Supplemental table 3

Supplemental table 4

Supplemental table 5

Supplemental table 6

Supplemental table 7

Supplemental table 8

Supplemental table 9

Supplemental table 10

Supplemental table 11

Supplemental table 12

Supplemental table 13

Supplemental table 14

Supplemental table 15

Supplemental table 16

Supporting data values

## Version 1 10/31/2023

In-Press Preview

## Version 2 01/02/2024

Electronic publication
