# Supplementary material for: Single-cell transcriptomics and chromatin accessibility profiling elucidate the kidney-protective mechanism of mineralocorticoid receptor antagonists
Source: J Clin Invest. 2024 Jan 2;134(1):e157165. doi: 10.1172/JCI157165 (PMC10760974; doi:10.1172/JCI157165)
Supplement: Supplemental data [file jci-134-157165-s009.pdf]

Supplementary Information

Title: Single-cell transcriptomics and chromatin accessibility profiling elucidate the kidney protective mechanism of mineralocorticoid receptor antagonists

Name of Authors:

Amin Abedini[1,2,3], Andrea Sanchez Navaro[1,2,3], Junnan Wu[1,2,3], Konstantin A. Klötzer[1,2,3], Ziyuan Ma[1,2,3], Bibek Poudel[1,2,3], Tomohito Doke[1,2,3], Michael S. Balzer[1,2,3], Julia Frederick[1,2,3], Hana Cernecka[4], Hongbo Liu[1,2,3], Xiujie Liang[1,2,3], Steven Vitale[1,2,3], Peter Kolkhof[4], Katalin Susztak[1,2,3]

[1]Renal, Electrolyte, and Hypertension Division, Department of Medicine, University of Pennsylvania, Perelman School of Medicine, Philadelphia, PA 19104, USA

[2]Institute for Diabetes, Obesity, and Metabolism, University of Pennsylvania, Perelman School of Medicine, Philadelphia, PA 19104, USA

[3]Department of Genetics, University of Pennsylvania, Perelman School of Medicine, Philadelphia, PA 19104, USA

[4]BAYER AG, Pharmaceuticals, Research & Development, Cardiovascular Research, Wuppertal, Germany

Correspondence:

Katalin Susztak, MD, PhD

Professor of Medicine

University of Pennsylvania, Perelman School of Medicine

3400 Civic Center Blvd,

Smilow Translational building 12-123,

Philadelphia, PA 19104

Phone: (215) 898-2009

ksusztak@pennmedicine.upenn.edu

Key words: Finerenone, Rat, Single nuclei RNA-seq, Single nuclei ATAC-seq

Supplementary Tables:

Supplementary Table 1. Cell type marker genes in snRNA-seq dataset.

Supplementary Table 2. Cell type specific open chromatin regions in snATAC-seq dataset.

Supplementary Table 3. Cell type specific genes calculated by gene activity snATAC-seq dataset.

Supplementary Table 4. Cell type specific genes in integrated snRNA-seq and snATAC-seq datasets.

Supplementary Table 5. Differentially activated ChromVAR motif.

Supplementary Table 6. List of MR and GR target genes.

Supplementary Table 7. List of differentially expressed genes between DOCA, control and MRAs including subclustered cell types and MRA-sensitive genes.

Supplementary Table 8. List of differentially accessible regions between DOCA, control and MRA-treated groups.

Supplementary Table 9. Mean expression values of DEGs in different cell types between DOCA, control, and MRAs groups.

Supplementary Table 10. Common and specific genes affected by different medications in the DOCA treated rats.

Supplementary Table 11. TPM of differentially expressed genes in bulk RNA-seq between DOCA and control groups.

Supplementary Table 12. The gene lists of factors 1 and 4 correlated with main phenotypes using tensor decomposition analysis.

Supplementary Table 13. The gene lists of specific modules of principal cells, proximal straight tubule cells, and injured PT cells correlated with main phenotypes using tensor WGCNA analysis.

Supplementary Table 14. Differentially expressed genes along the pseudotime trajectory in snRNA-seq.

Supplementary Table 15. Differentially accessible regions along the pseudotime trajectory in snATAC-seq.

Supplementary Table 16. Clinical characteristics of 991 human kidney tubule samples.

Supplemental Methods:

Single nuclei RNA sequencing

Nuclei were isolated using lysis buffer (83). In 1 ml of lysis buffer, 10-30 mg of frozen kidney tissues were minced with a razor blade into 1-2 mm pieces**.** Tissue was transferred into a dounce homogenizer and homogenized in 1 ml of lysis buffer using pestle A and B. The homogenized tissue was filtered through a 40 µm strainer (08-771-1, Fisher Scientific) and the strainer was washed with 2 ml of wash buffer. Nuclei were centrifuged at 500xg for 5 minutes at 4°C. The pellet was resuspended in wash buffer, filtered through a 40 µm Flowmi cell strainer (BAH136800040-50EA, Sigma Aldrich). Nuclei were reviewed and counted under a microscope. Next, 30,000 cells were loaded into the Chromium Controller (10X Genomics, PN-120223) on a Chromium Next GEM chip G Single Cell Kit (10X Genomics, PN-1000120) to generate single cell gel beads in the emulsion (GEM) according to the manufacturer's protocol (10X Genomics, PN-1000121). The cDNA and library were made using the Chromium Next GEM Single Cell 3′ GEM Kit v3.1 (10X Genomics, PN-1000121) and Single Index Kit T Set A (10X Genomics, PN-120262) according to the manufacturer's protocol. In addition, five samples were processed using Chromium Next GEM Single Cell Multiome ATAC Library + Gene Expression Kit (10X Genomics, PN-1000283) and loading the nuclei on Chip J and making the cDNA and libraries by using the library construction kit (10X Genomics, PN-1000282). Quality control for the libraries were performed using Agilent Bioanalyzer High Sensitivity DNA kit (Agilent Technologies, 5067-4626). Libraries were sequenced on Illumina Novaseq 6000 system with 2 × 150 paired-end kits using the following demultiplexing: 28 bp Read1 for cell barcode and UMI, 8 bp I7 index for sample index and 91 bp Read2 for transcript. For five samples which were made using Chromium Next GEM Single Cell Multiome ATAC Library + Gene Expression Kit, the following demultiplexing protocol was used: 28 bp Read1 for cell barcode and UMI, 10 bp I7 and 10 bp I5 index for sample index and 90 bp Read2 for transcript.

Single nuclei ATAC sequencing

Kidneys were minced and lysed in 5 mL lysis buffer for 15 minutes. The lysis reaction was blocked by adding 10 mL 1× PBS into each sample, and solution was passed through a 40 µm cell strainer. Next, the solution was centrifuged at 30,000 × *g* for 45 min in 4°C. To remove the cell debris and cytoplasmic materials, Nuclei PURE Prep Nuclei Isolation Kit (Sigma, NUC-201) was used. Nuclei quality and concentration were measured with Countess AutoCounter (Invitrogen, C10227). The diluted nuclei were loaded and incubated in transposition mix from Chromium Single Cell ATAC Library & Gel Bead Kit (10X Genomics, PN-1000110). For capturing the GEMs, Chromium Chip E (10X Genomics, PN-1000082) in the Chromium Controller was used. Next, snATAC libraries were generated using the Chromium Single Cell ATAC Library & Gel Bead Kit and Chromium i7 Multiplex Kit N Set A (10X Genomics, PN-1000084) according to the manufacturer's instruction. In addition, five samples were made using Chromium Next GEM Single Cell Multiome ATAC Library + Gene Expression Kit (10X Genomics, PN-1000283) and loaded on Chip J and for snATAC-seq libraries using Chromium Single Cell ATAC Library & Gel Bead Kit and Chromium i7 Multiplex Kit N Set A (10X Genomics, PN-1000212). Agilent Bioanalyzer High Sensitivity DNA kit was used to check the quality of the libraries. Libraries were sequenced on Illumina Novaseq 6000 system with 2 × 50 paired-end kits followed by demultiplexing as follows: 50 bp Read1 for DNA fragments, 8 bp i7 index for sample index, 16 bp i5 index for cell barcodes and 50 bp Read2 for DNA fragments. For five samples which were made using Chromium Next GEM Single Cell Multiome ATAC Library + Gene Expression Kit, the following demultiplexing protocol was used: : 50 bp Read1 for DNA fragments, 8 bp i7 index for sample index, 24 bp i5 index for cell barcodes and 49 bp Read2 for DNA fragments.

Bioinformatic analysis

Primary single nuclei RNA-seq data processing

FASTQ files from each 10X single nuclei run were processed by Cell Ranger v6.0.1 (10X Genomics). A Rattus norvegicus genome reference (rn6) was used to generate gene expression matrix for each cell. For alignment the "--include-introns" option was used. For the libraries were made using Chromium Next GEM Single Cell Multiome ATAC Library + Gene Expression Kit, "--chemistry=ARC-v1" function was used.

Data Processing and Computational Analyses

Seurat objects from the aligned outputs (from multiple samples) were created after ambient RNA removal using SoupX (89) where genes expressed in more than 3 cells and cells with at least 300 genes were retained. Then, doublets of each sample were removed using "DoubletFinder" (88). Then, a merged Seurat object was obtained using "merge" function of Seurat v (4.0.3)(84). The following QC filtering were used on the merged object: (a) cells having n_feature counts of more than 3000 and less than 200 as well as (b) more than 15% mitochondrial counts were filtered.

Data Normalization and Cell Population Identification

First, using the "vst" method, highly variable genes were identified. The data was scaled, and natural log transformed. Principal component analysis (PCA) was used to reduce the linear dimension of the scaled values. We used the "harmony"(35) package by "RunHarmony" function for batch effect correction. Based on Euclidean distances between cells in a multidimensional PC space (the first 30 PC were used) and a fixed number of neighbors per cell, a shared nearest

neighbor network was created, which was then used to generate a 2-dimensional Uniform Manifold Approximation and Projection (UMAP) for visualization.

We used Seurat's "FindAllMarkers" function of "Seurat" to find cell-type markers. This method computes log fold changes, percentages of expression within and outside a group, and Wilcoxon-Rank Sum test p-values comparing a group to all cells outside that specific group, including multiple testing adjustment. FDR < 0.05 and a log-fold-change threshold of 0.25 were considered significant.

DEGs between groups

We used the "FindMarkers" function for each cell type, for each condition and a log-fold-change threshold of 0.25, pct_1 of 0.1, and FDR<0.05 to identify the DEGs between experimental groups were used.

Tensor Decomposition

We used scITD 1.0.2 (60) to perform tensor decomposition analysis. All samples, DOCA and treated groups were included in the analysis. We used the pseudobulk tensor using the function form tensor with the default parameters. The number of extracted factors was determined by using the function determine ranks tucker with the parameters num iter=10 and var scale power=2. We validated the factor number further by evaluating the stability of the factors using the function run stability analysis with parameters sub prop=0.95 and n iterations=50, which revealed that all three factors had mean donor scores correlations close to one. The Tucker tensor decomposition was then performed using the function run tucker ica. Finally, we used the function get lm pvals to identify genes that were significantly associated with each factor. Significant genes in each factor per cell types are shown on the heatmap.

Single nuclei WGCNA

We used the R packages hdWGCNA (61, 62) and WGCNA (59) to perform WGCNA on PC, PST, and iPT cells. We used the hdWGCNA function to construct metacells to aggregate transcriptionally neighboring single cells from the rats into pseudo-bulk metacells. Then, the metacells were processed by regular Seurat function including "NormalizeMetacells ", "ScaleMetacells", "RunPCAMetacells", "RunHarmonyMetacells", and "RunUMAPMetacells" were performed. Then, the expression matrix was set for PC, PST, and iPT, separately. Next, after selecting the appropriate softpower by running "TestSoftPowers", co-expression network was created. In the next step harmonized module eigengenes was calculated to summarize the gene expression profile for each gene and in each sample, module connectivity was calculated, and genes assigned to each module was extracted and the results were shown as the average gene expression in all clusters. Also, the module eigengenes for each group was calculated and was visualized on a heatmap.

Single nuclei RNA-seq trajectory analysis

For the trajectory analysis, PST and iPT cells were subclustered. Following sub-clustering, equal numbers (n=200) of PST and iPT sub-clusters were randomly subsampled, and a cell dataset object (CDS) was created using Monocle3 (63, 64). The dataset was embedded for dimension reduction and pseudotemporal ordering after preprocessing and batch effects correction. We used the "order cell" function with the PST as the starting point for the "pseudotime" analysis. The "track genes" function was used to determine the DEGs along the trajectory of injured PT, and genes with q values 0.05 were considered significant.

Ligand–receptor interactions

From snRNA-seq data, the CellChat (67) repository was used to assess cellular interactions between different cell types and infer cell-cell communication networks. The CellChat v1.4.0 package was used to forecast cell type-specific ligand-receptor interactions (mouse dataset). Only receptors and ligands found in more than ten cells per cluster were considered. The probability and P value for each interaction were computed. Each experimental group was analyzed separately and then combined.

Single nuclei ATAC-seq analysis

FASTQ raw files were aligned to the rn6 reference genome and quantified with Cell Ranger ATAC (v. 1.1.0). For the libraries were made using Chromium Next GEM Single Cell Multiome ATAC Library + Gene Expression Kit, "--chemistry=ARC-v1" function was used. Signac was used to embed the cell ranger outputs of eleven snATAC-seq datasets (v.1.3.0) (40) to generate Signac object. Then, the Signac objects of eleven samples were merged together using "merge" function. The following criteria were used to remove low-quality cells from each snATAC object: peak region fragments < 3000 & peak region fragments > 20000 & pct reads in peaks < 15 & nucleosome signal > 4 & TSS.enrichment < 2). The filtered cells in eleven objects were used for the downstream analysis. To reduce dimensions, the TFIDF matrix and UMAP were decomposed using singular value decomposition (SVD). The Louvain algorithm was used to generate a KNN graph. Harmony was used to correct the batch effect (35) via the "RunHarmony" function in Seurat.

Cluster annotation

Differential accessible chromatin regions (DARs) between cell types were assessed by the Signac "FindMarkers" function for peaks detected in at least 25% of cells using a likelihood ratio test and a log-fold-change threshold of 0.25 and FDR<0.05. ChIPSeeker (v1.24.0)(85) was used to annotate the Genomic regions containing snATAC-seq peaks.

Annotation based on snRNA-seq and Integration snATAC-seq and snRNA-seq

After clustering of the four integrated sn_ATAC-seq datasets, a gene activity matrix was generated using "GeneActivity" function in Signac. This analysis counts the ATAC peaks within the gene body and 2 kb upstream of the transcriptional start site for the Ensembl annotated protein-coding genes. The gene activity matrix was then log normalized. Next, the snRNA-seq dataset was used as a reference and by using "FindTransferAnchors" function, shared correlation patterns in the gene activity matrix and snRNA-seq dataset were generated. Using the "TransferData" function, the predicted labels within two datasets were determined.

For integration the snRNA-seq and snATAC-seq, after normalization, the variable genes in each dataset were calculated. Then, by using the "FindIntegrationAnchors" function, the anchors between two datasets were identified. After finding anchors, the two datasets were integrated using "IntegrateData" function. After scaling and dimensionality reduction, integrated datasets were projected to the UMAP and clustering were performed.

Motif Enrichment Analysis and Motif Activities

Motif enrichment analysis was performed using "AddMotifs" function of Signac after generating a matrix of positional weight matrices of motif candidates from JASPAR2020. To calculate the

transcription factor activity, chromVAR (v.1.6.0)(42) and the related function of "RunChromVAR" were used. Differential motif activities between clusters were computed using the "FindMarkers" function and FDR<0.05 was considered as significant.

DARs between groups

We used the "FindMarkers" function after selecting "DefaultAssay" as "peaks" to identify DARs in each cell type, with a log-fold-change threshold of 0.25 and FDR<0.05. Peaks translated to related genes using ChIPSeeker (v1.21.1)(85). The up-regulated DARs between DOCA and control groups were considered as MR targets found in the present study and the list was provided.

Trajectory analysis on snATAC-seq and integrated datasets

To run trajectory analysis on PT cells; the PST and iPT cell types on sATAC-seq dataset were sub-clustered. Equal number of PST and injured PT sub-clusters (n=200) were randomly subsampled and a CDS object was generated. The CDS was preprocessed and batch effect was removed using monocle3(64). The results were reduced into UMAP and cells were clustered. Next, cell ordering was performed using "order_cell" function and pseudotime trajectory analysis was performed by selecting PST as a "start point" and "learn_graph" function. To determine the DARs along the trajectory of injured PT cells "track_genes" function was used and peaks with q_values<0.05 were considered significant. Peaks were translated to related genes using ChIPSeeker (v1.21.1). The data were visualized with "plot_accessibility_in_pseudotime" or "plot_cells" functions.

Gene regulatory network inference

To identify TFs and their target genes in PST and iPT cell types, the cis-regulatory analysis using the R package SCENIC (65) v1.1.2.2 was performed. In this analysis the gene regulatory network based on co-expression and DNA motif analysis were used to identify the TFs and the

target genes. In brief, the "GENIE3" is used to identify TFs, modules created and then by using "RcisTarget" subjected to *cis*-regulatory motif analysis with gene-motif rankings of 10 kb around the TSS or 500 bp upstream.

Rat and Human Bulk RNA-seq Analysis

FASTQC was used to validate the sequencing results. TrimGalore was then used to trim the adapters and low-quality bases (v0.4.5). STAR was used to align the trimmed fastq files to the rat genome (rn6/Rnor 6.0) (v2.7.3a) (86, 87) or to the human genome (hg19/GRCh37) applying STAR (v2.7.3a) based on GENCODE v19 annotations(86, 87). RSEM was used to quantify gene expression by calculating uniquely mapped reads as transcripts per million (TPM).

Principal component analysis (PCA) plot was performed to identify outliers. To determine DEGs between experimental rat groups, edgeR package was used. First, cpm matrix was generated, then genes with raw count greater than 4 (number of groups) were kept. The "DEGList" function was used to determine the DEG between different groups. Log2 fold change of 0.5 and FDR<0.05 were considered as significant.

The WGCNA (59) on rat bulk RNA-seq was performed using "WGCNA" package. First, after filtering the genes with zero expression in all samples, the appropriate "soft_power" by running "pickSoftThreshold" function was determined. After setting the power, network construction and module detection were done using "blockwiseModules" function. After determining the modules, colors, and eigengenes of each module, correlation with each phenotype was calculated.

Clustering analysis

PCA analysis and Clustering based on rat's lab and histological data

To identify clusters based on clinical, laboratory, and histological findings in rats, PCA analysis and hierarchical clustering were performed. For PCA analysis all the blood pressure, lab and histological data were included in the analysis and after scaling, the results were shown by PCA plot. Hierarchical clustering was performed on the scaled data using the Ward's method with Euclidean distances. The optimal number of clusters was determined by average silhouette method. After clustering, data was displayed as a cluster dendrogram.

Clustering of microdissected human kidney tubule samples based on injured PT gene signature

Based on the rat injured PT gene signature using the top genes along the trajectory, hierarchical clustering was performed on the scaled TPM matrix of microdissected human tubules dataset. The datasets were clustered using Ward's method with Euclidean distances. The average silhouette method was used to determine the optimal number of clusters. Data after clustering was displayed as a cluster dendrogram.

Supplementary Figures

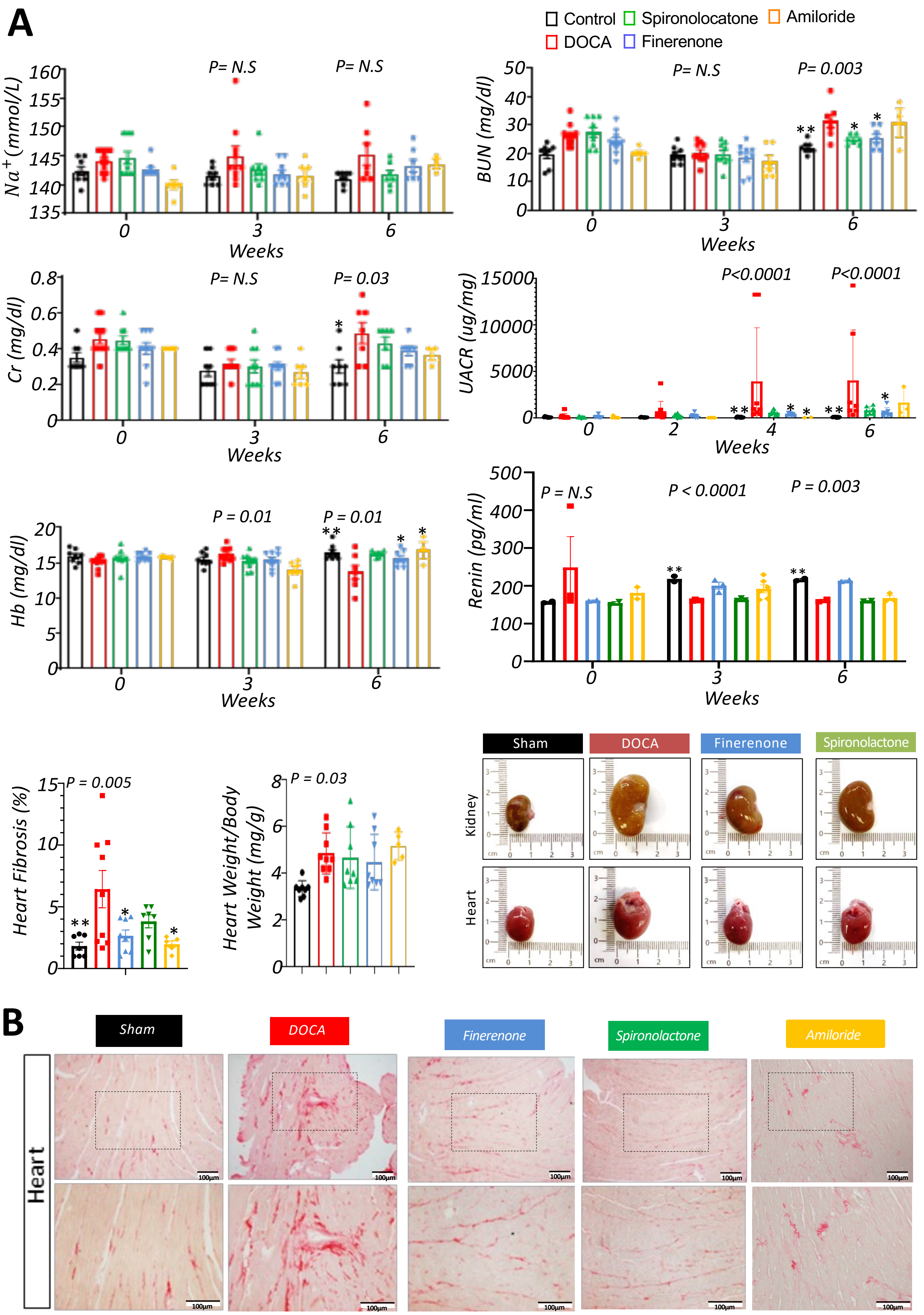

Supplementary Fig. 1. Laboratory and heart histology findings of the experimental groups at different time points. (A) Comparing different laboratory parameters at different time points and representative macroscopic changes in kidneys and hearts in experimental groups. The comparison was done using a One-way ANOVA test. A student's t-test was used to compare each group to the DOCA-treated group. Bars indicate SEM. **(B)** Sirius red staining of the hearts of different experimental groups (magnification is 20X). DOCA; deoxycorticosterone acetate, Hb; hemoglobin, Cr; creatinine, Na+; sodium, UACR; urine albumin to creatinine ratio, BUN; blood urea nitrogen *P<0.05, **P<0.01.

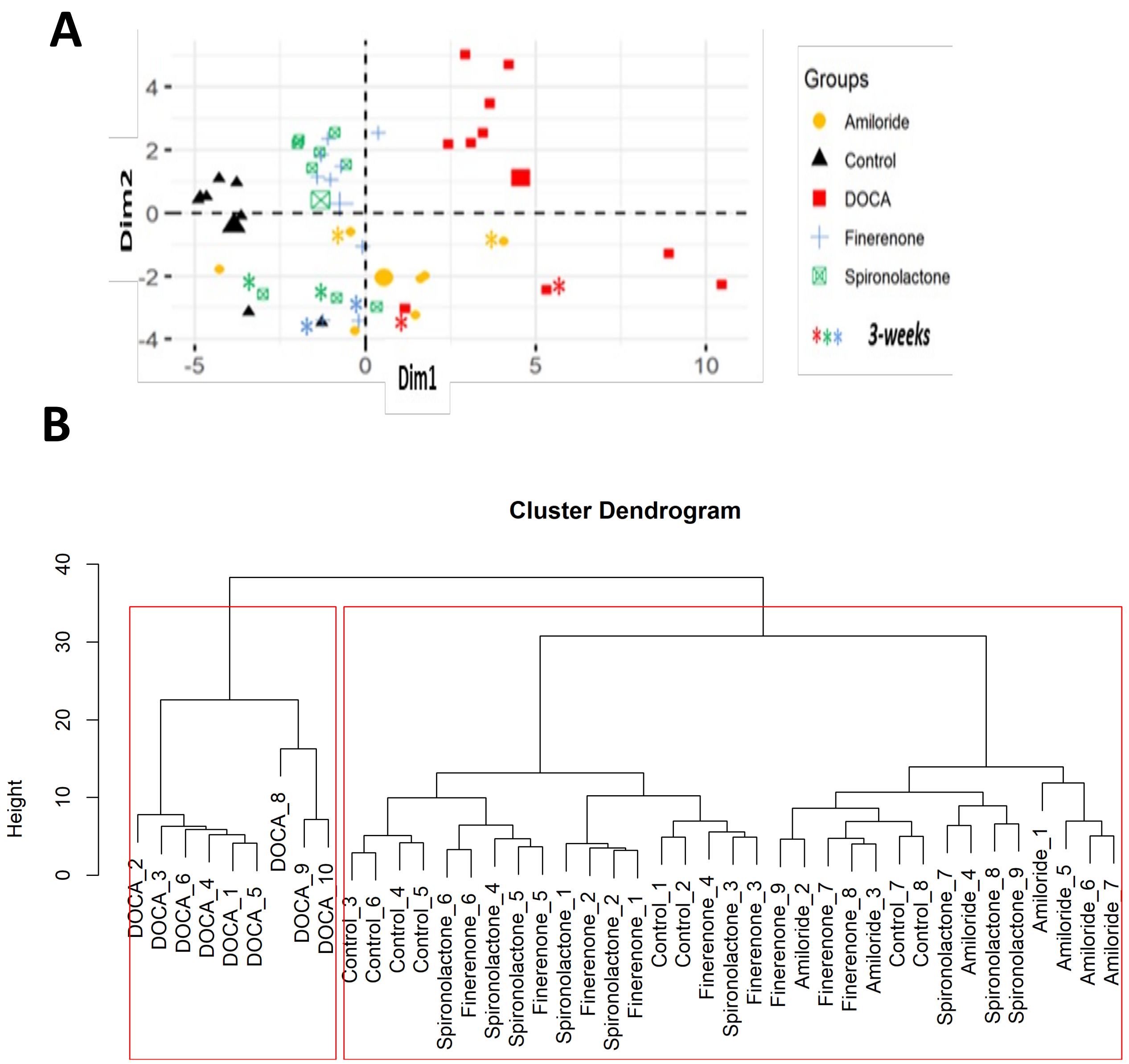

Supplementary Fig. 2. Clustering of the samples based on blood pressure, lab data, and histological scores. (A) PCA plots of the samples in different experimental groups. Each group is represented by a color (Control: black; DOCA: red; Finerenone: blue; Spironolactone: green; Amiloride: yellow). Asterisk (*) shows the 3-week collection. **(B)** Hierarchical clustering of the samples based on phenotype similarity. The DOCA group was completely separated from the remaining groups. Two DOCA+vehicle treated rats sacrificed after three weeks of DOCA administration were excluded from this analysis due to missing phenotype information.

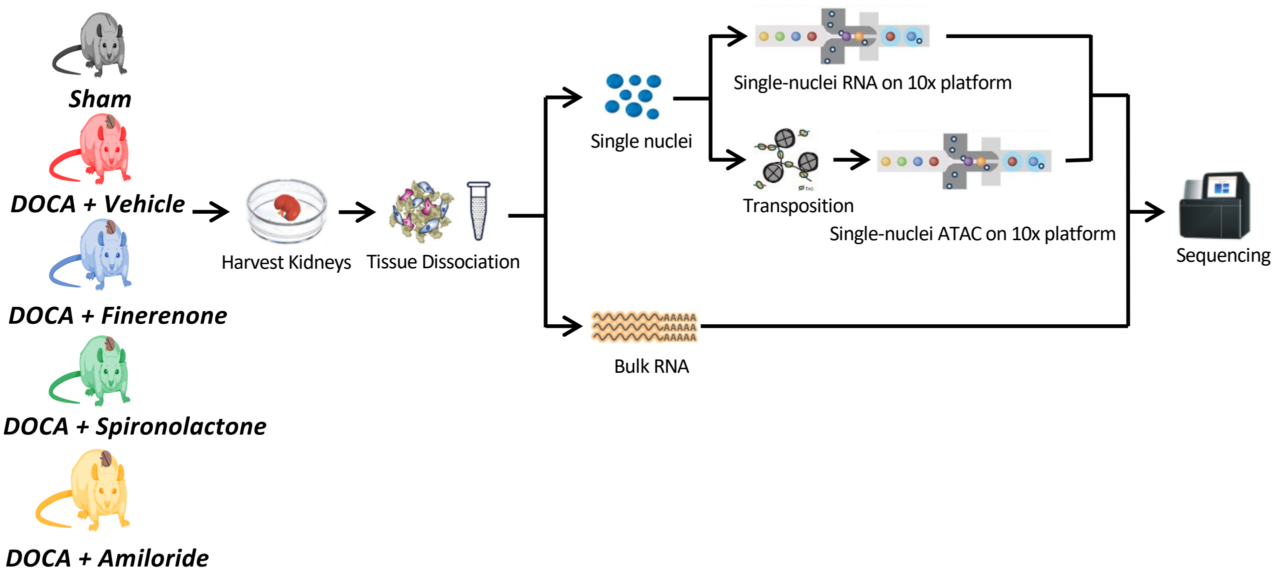

Supplementary Fig. 3. The study overview. DOCA; deoxycorticosterone acetate.

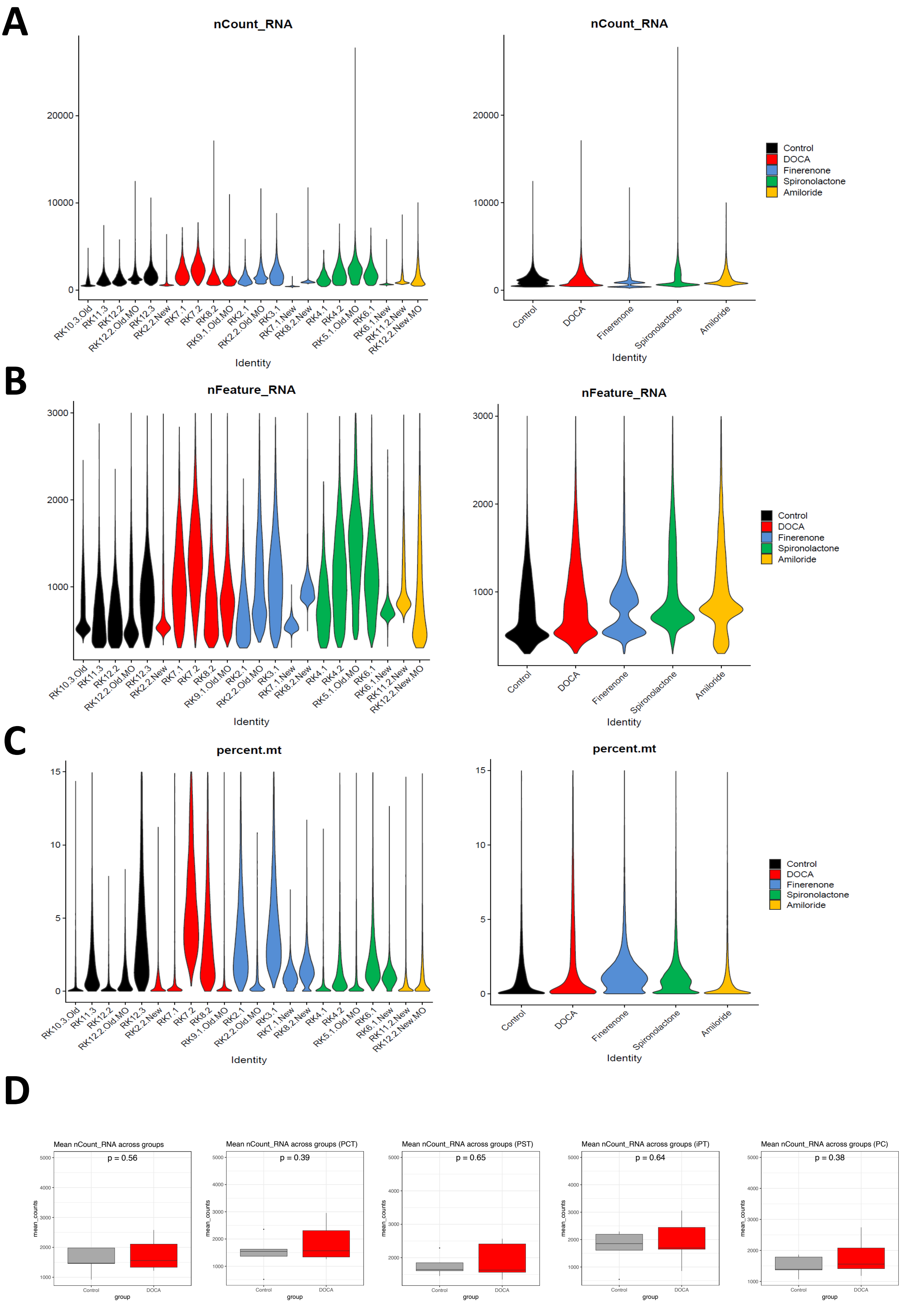

Supplementary Fig. 4. Quality control parameters of the snRNA-seq dataset. (A) n_Count_RNA in different samples and different experimental groups. **(B)** n-feature RNA in different samples and different experimental groups. **(C)** Percent.mt in different samples and different experimental groups. **(D)** Mean n_Counts per sample across groups (Control vs DOCA) after quality control across all cells and four cell types (PCT, PST, iPT, PC). Boxplots representing the distribution of mean RNA counts. The median RNA count for each group is depicted by the bold horizontal line in each box, while the interquartile range is represented by the box itself. P-values based on Welch Two Sample t-test. PCT; proximal convoluted tubule, PST; proximal straight tubule, iPT; injured proximale tubule, PC; principal cells of collecting ducts.

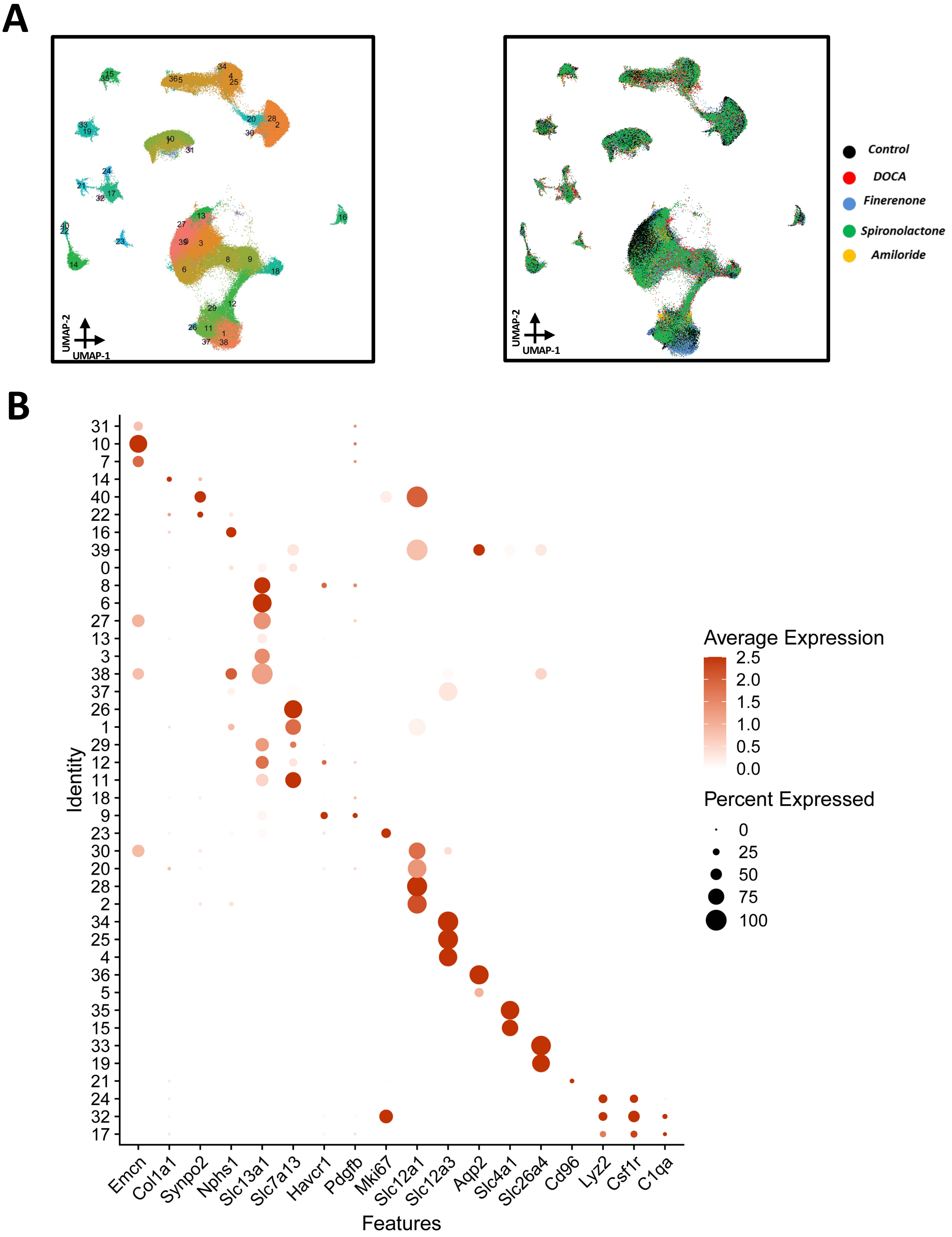

Supplementary Fig. 5. Unbiased clustering of the snRNA-seq dataset. (A) UMAP representation of all samples using Harmony integration (left panel) and integration of all samples (right panel). Unbiased clustering identifies 41 clusters. **(B)** Bubble dot plot of cluster specific marker genes used for cell type annotation in the snRNA-seq data. The size of the dot indicates percent of cells expressing the marker, the darkness of the color indicates average expression.

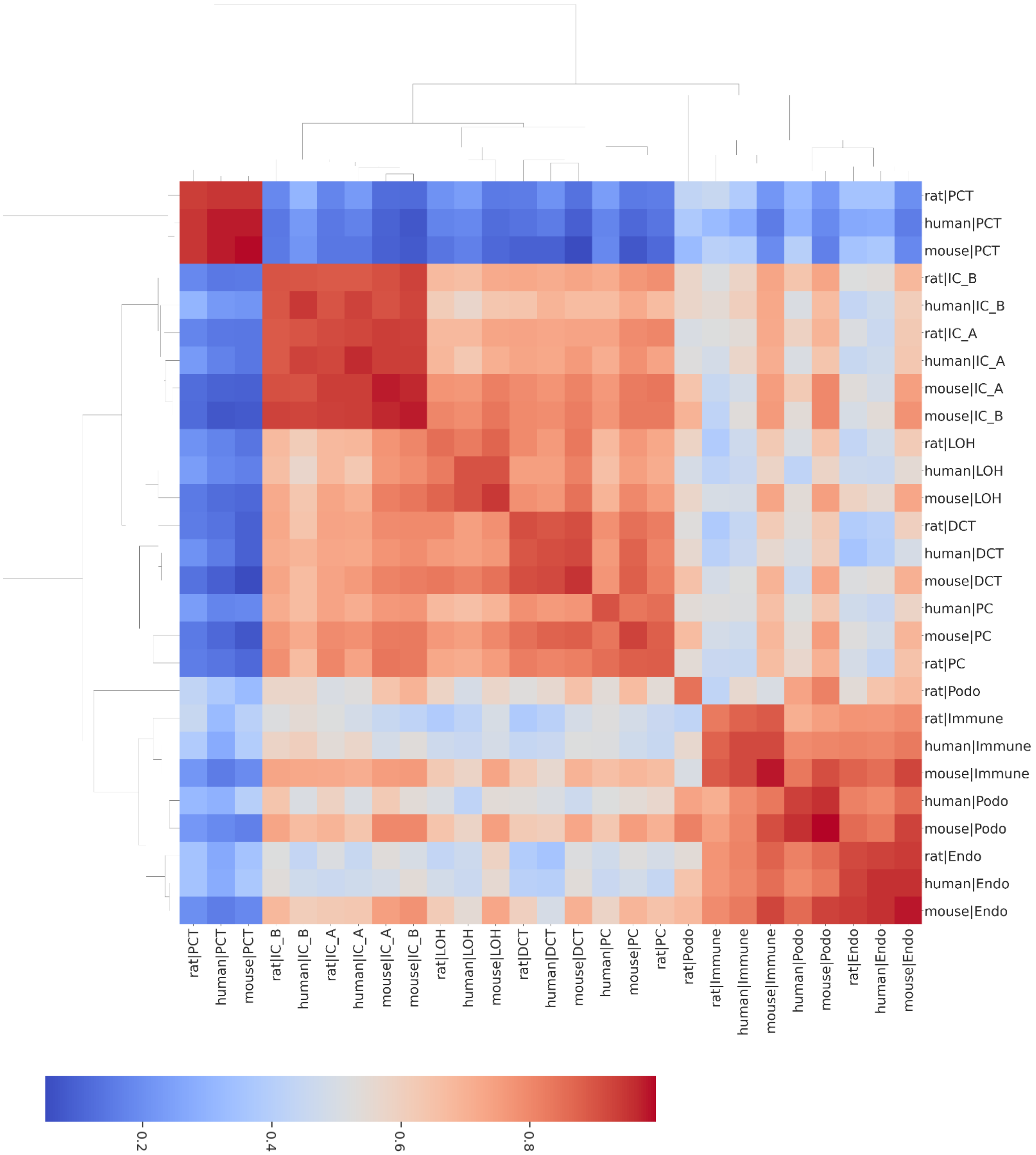

Supplementary Fig. 6. Comparison of kidney clusters and replicability across different species. MetaNeighbor analysis quantifying cell type consistency between mouse, human and rat data considering one-to-one orthologous genes. The heatmap visualizes the mean area under the receiver operator characteristic curve (AUROC) measuring cell type similarity across datasets. For this analysis, the lymphocyte, monocyte, and macrophage clusters were merged and called "Immune." Endo; endothelial cells, Podo; podocyte, PCT; proximal convoluted tubule, LOH; loop of Henle, DCT; distal convoluted tubule, PC; principal cells of collecting ducts, IC_A; Type A intercalated cells, IC_B; Type B intercalated cells.

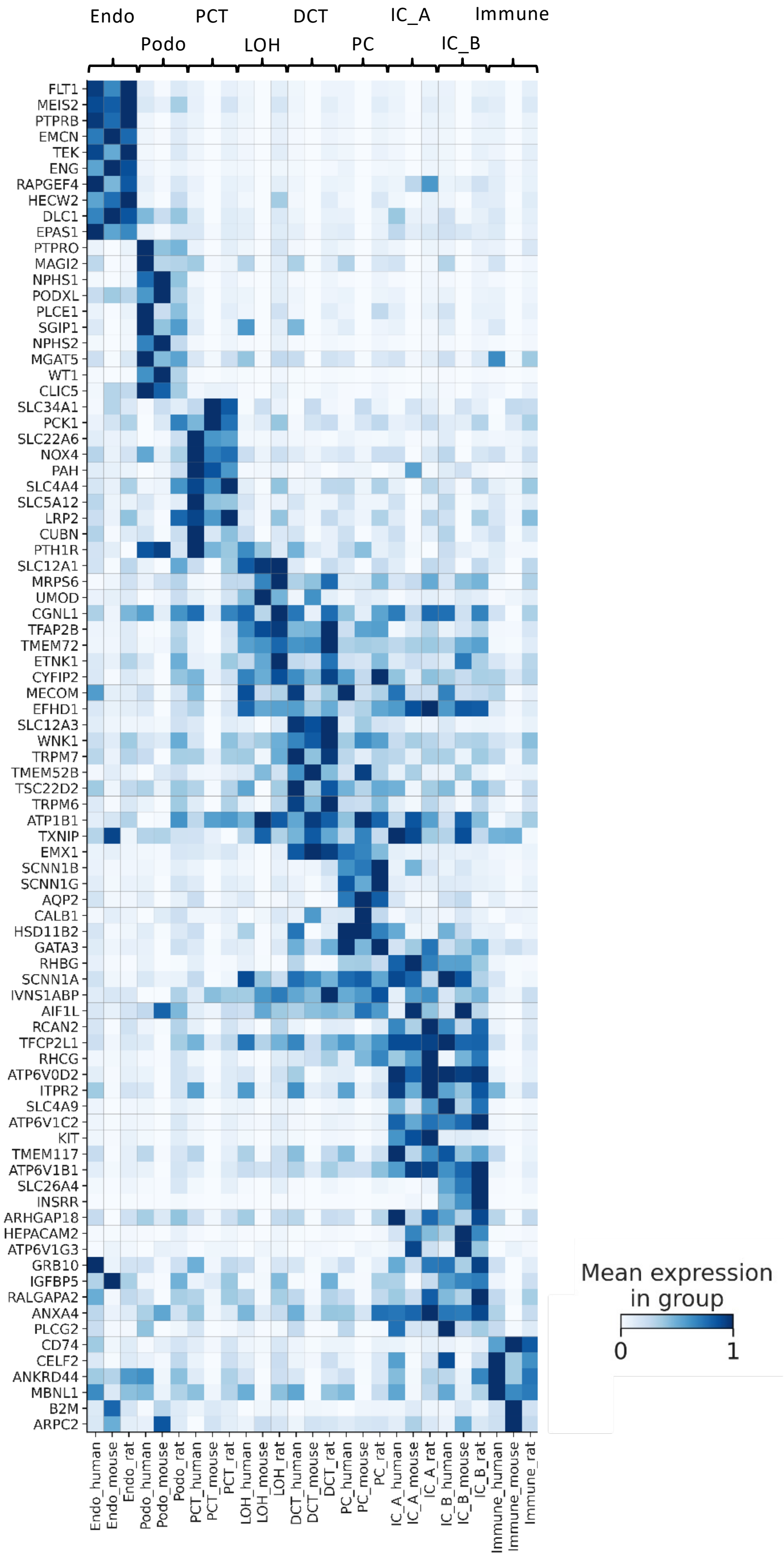

Supplementary Fig. 7. Comparison of marker genes across different species. The top common and one-to-one ortholog marker genes from the rat snRNA-seq dataset, mice and human datasets were compared. Datasets were merged after gene symbol harmonization and normalization. Scaled expression of each gene in each cluster of every species is shown in rows. Columns are cell type clusters from a single species. For this analysis, the lymphocyte, monocyte, and macrophage clusters were merged and called "Immune." Endo; endothelial cells, Podo; podocyte, PCT; proximal convoluted tubule, LOH; loop of Henle, DCT; distal convoluted tubule, PC; principal cells of collecting ducts, IC_A; Type A intercalated cells, IC_B; Type B intercalated cells.

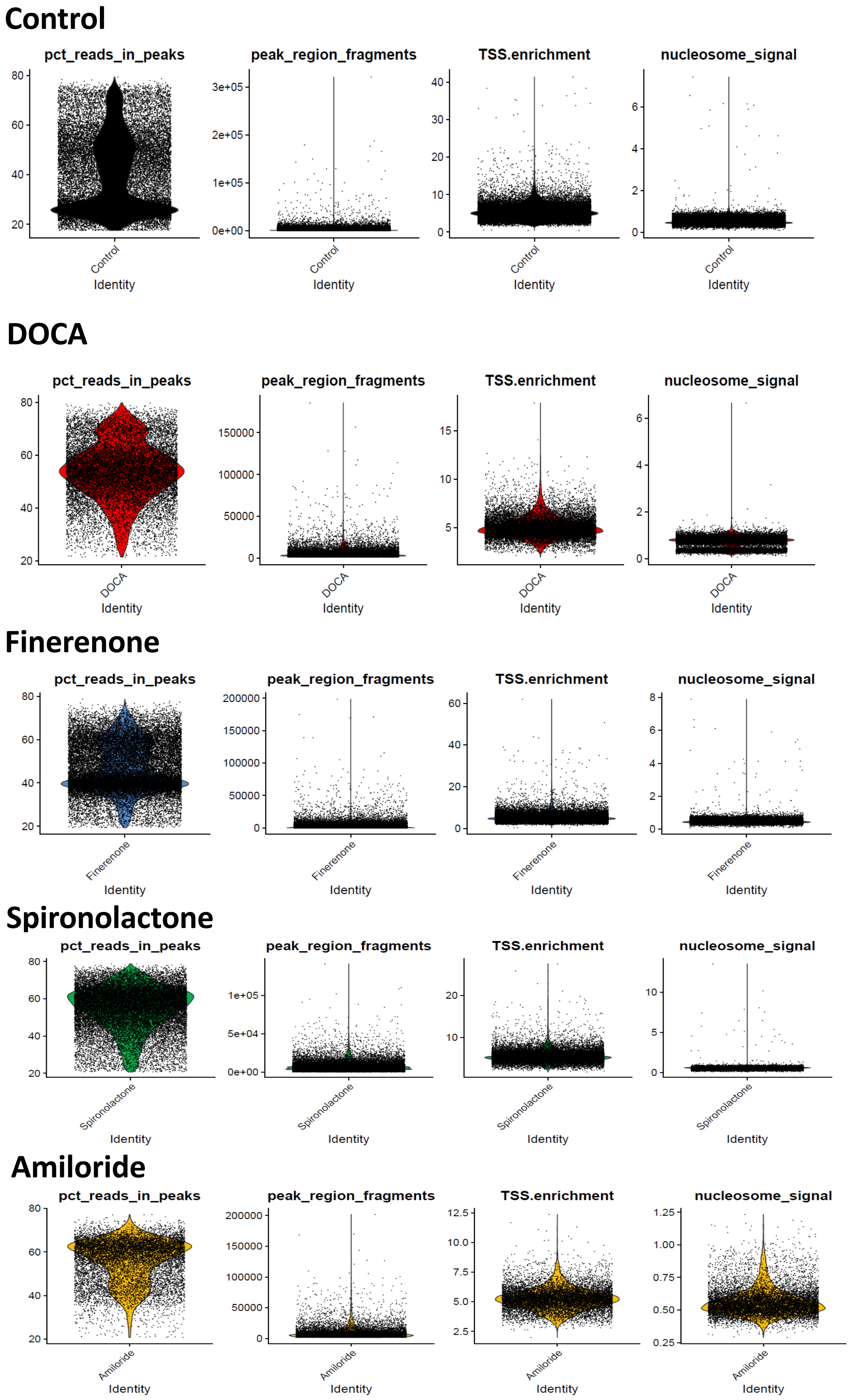

Supplementary Fig. 8. Quality control parameters of the single-nuclei ATAC-seq dataset. The figure represents the pct reads in peaks, peak region fragments, TTS enrichment, and nucleosome signal in 5 different studied groups. Each group contains two samples, while amiloride group contains only one sample.

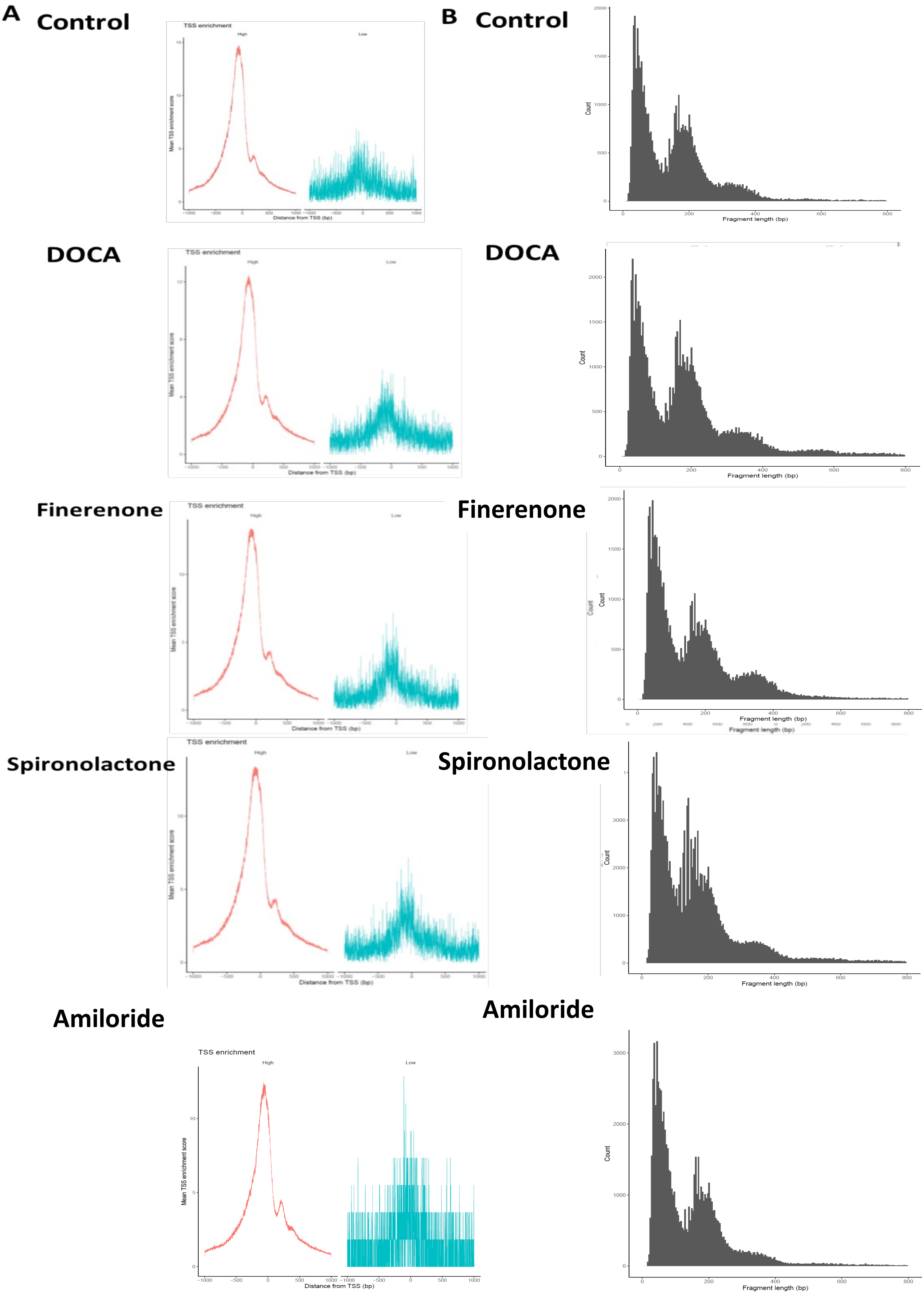

Supplementary Fig. 9. TSS enrichments and fragment length periodicity of different samples in the snATAC-seq dataset. **(A)** The mean TSS enrichment score in each group. **(B)** The fragment length for all cells in each group.

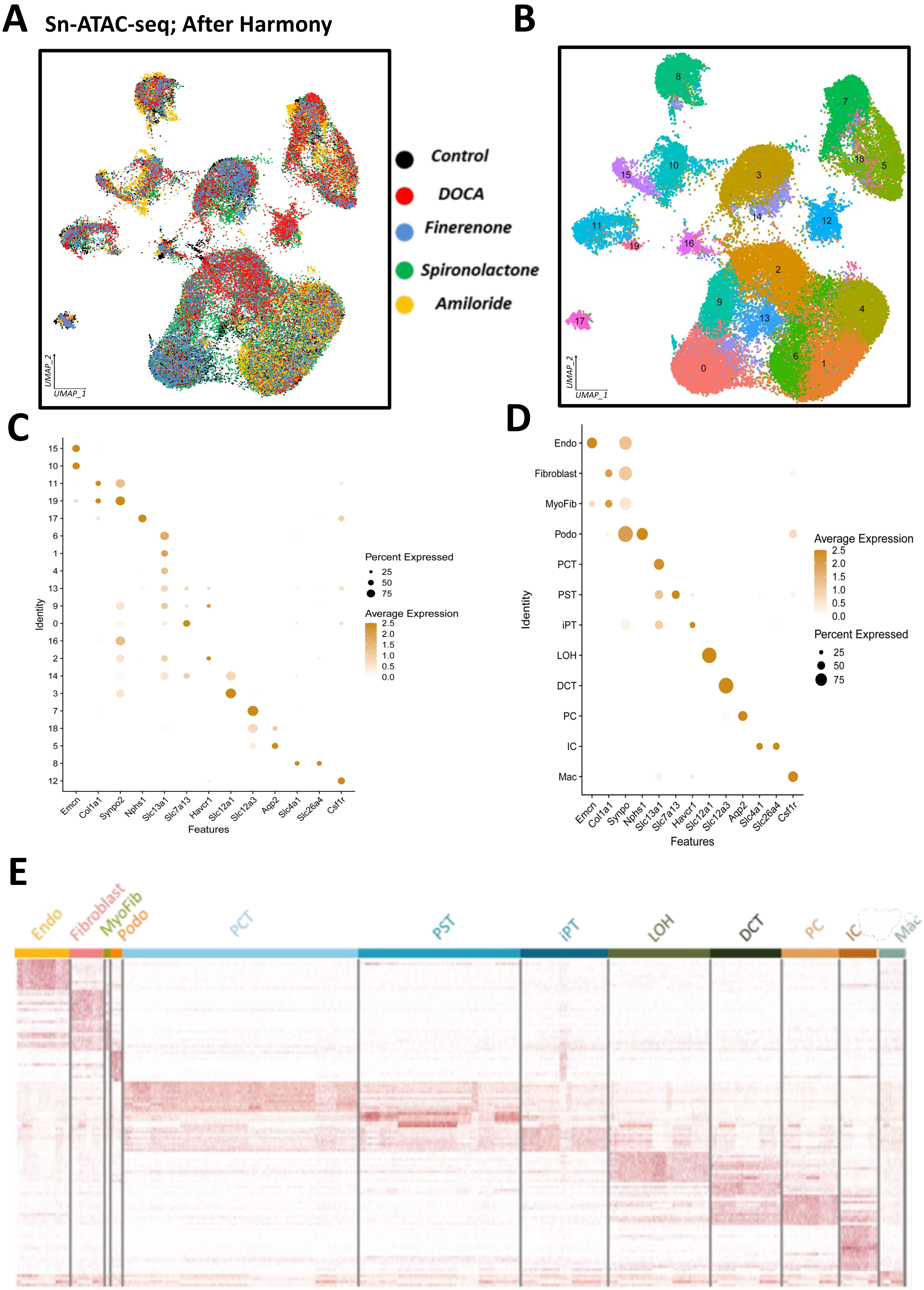

Supplementary Fig. 10. Rat kidney snATAC-seq data. (A) UMAP of snATAC-seq after using Harmony for batch effect correction, showing original sample identity. **(B)** UMAP of cell clusters (20 clusters identified). **(C)** Bubble dot plot of gene activity of marker genes in the original snATAC-seq data. **(D)** Bubble dot plot of gene activity of gene markers used for clustering the snATAC-seq dataset. The size of the dot indicates the expression percentage, and the darkness of the color indicates average expression. **(E)** Heatmap of top 10 accessible regions in each cluster of snATAC-seq. Endo; endothelial cells, Podo; podocyte, PCT; proximal convoluted tubule, PST; proximal straight tubule, Injured_PT; Injured proximal tubule cells, LOH; loop of Henle, DCT; distal convoluted tubule; PC; principal cells of collecting ducts, IC; intercalated cells.

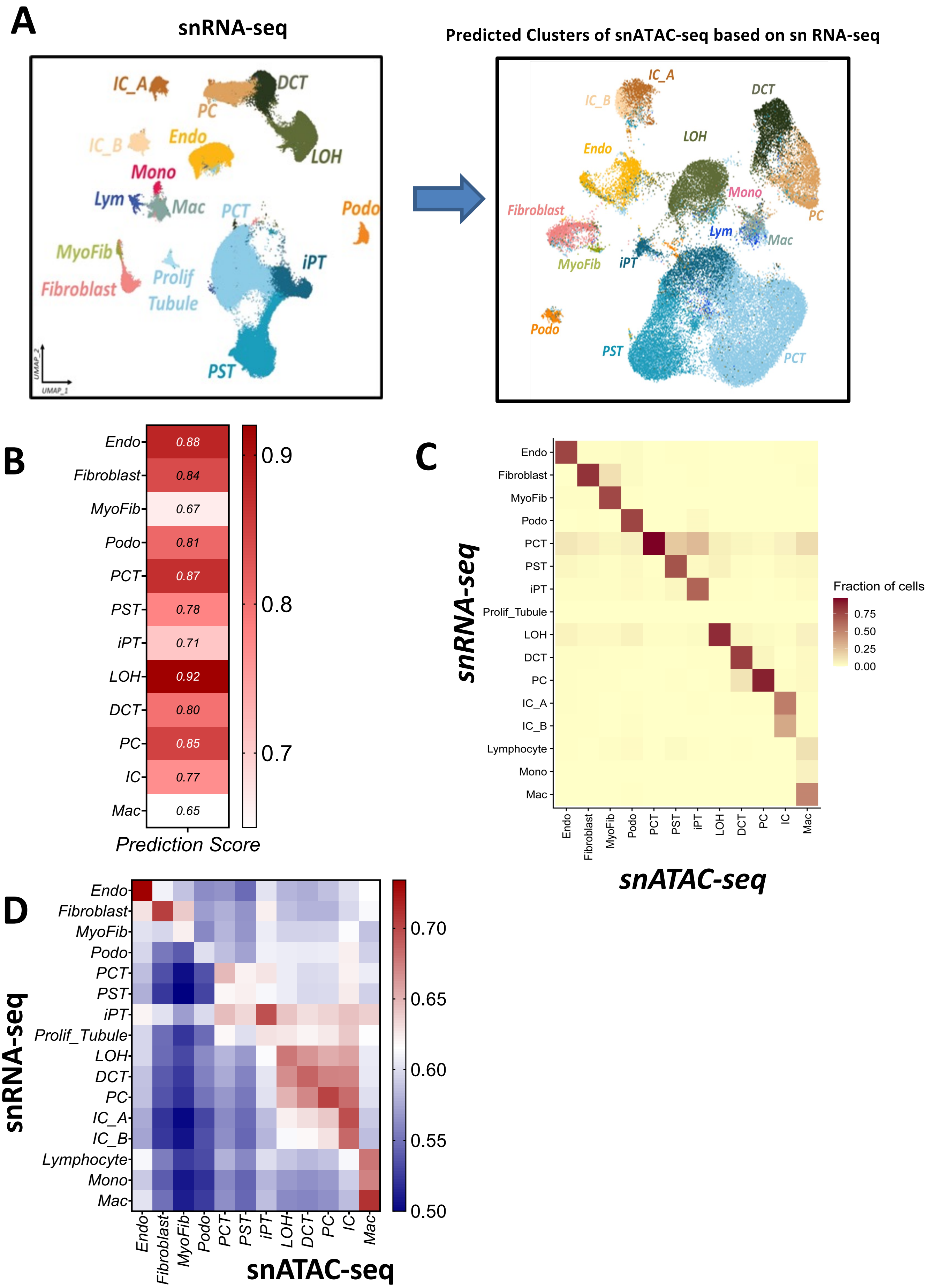

Supplementary Fig. 11. Integration of the snATAC-seq and snRNA-seq datasets. (A) UMAP of snRNA-seq dataset (left panel) and its use for the annotation of snATAC-seq dataset after label transfer. **(B)** Prediction score of clusters in snATAC-seq dataset based on snRNA-seq dataset using Signac. The score is calculated between 0 to 1 for each cell in snATAC-seq data and the mean of the score in each cluster is shown in the heatmap. **(C)** Heatmap of the prediction score in each cluster based on label transfer analysis from snRNA-seq to snATAC-seq. **(D)** Correlation between variable genes in each snRNA-seq and snATAC-seq datasets clusters. Endo; endothelial cells, MyoFib; myofibroblast, Podo; podocyte, PCT; proximal convoluted tubule, PST; proximal straight tubule, iPT; injured proximal tubule cells, Prolif_Tubule; proliferative tubule cells, LOH; loop of Henle, DCT; distal convoluted tubule; PC; principal cells of collecting ducts, IC; intercalated cells, IC_A; Type A intercalated cells, IC_B; Type B intercalated cells, Mono; monocyte, Macro; macrophage.

A Integrated snRNA-seq and snATAC-seq

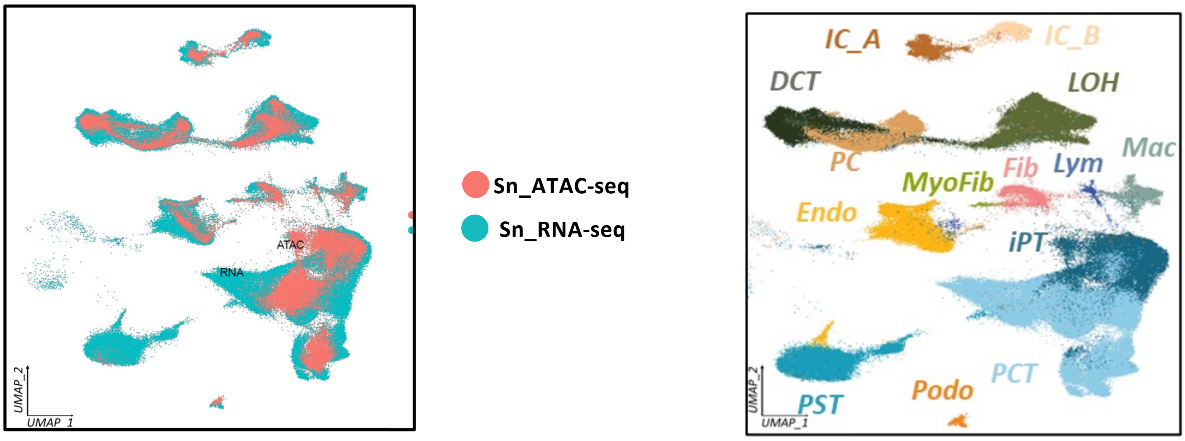

B Integrated Sn-RNA-seq and Sn-ATAC-seq

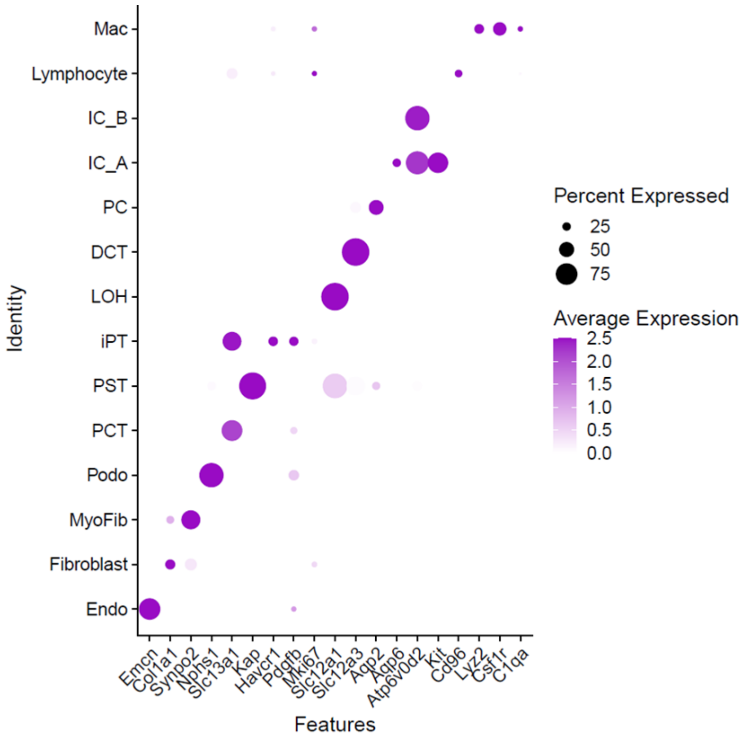

Supplementary Fig. 12. Integration of snRNA-seq and snATAC-seq datasets. (A) UMAP of integrated snRNA-seq and snATAC-seq based on data origin (left panel). The right panel shows the cell type annotation in the integrated dataset. **(B)** Bubble dot plot showing gene markers used for the annotation of the integrated snRNA-seq and snATAC-seq datasets. The size of the dots indicate expression percentages, and the darkness of the color indicates the average expression.

Endo; endothelial cells, Podo; podocyte, PCT; proximal convoluted tubule, PST; proximal straight tubule, Injured_PT; injured proximal tubule cells, Prolif_PT; proliferative proximal tubule cells, LOH; loop of Henle, DCT; distal convoluted tubule; PC; principal cells of collecting ducts, IC; intercalated cells, IC_A; Type A intercalated cells, IC_B; Type B intercalated cells.

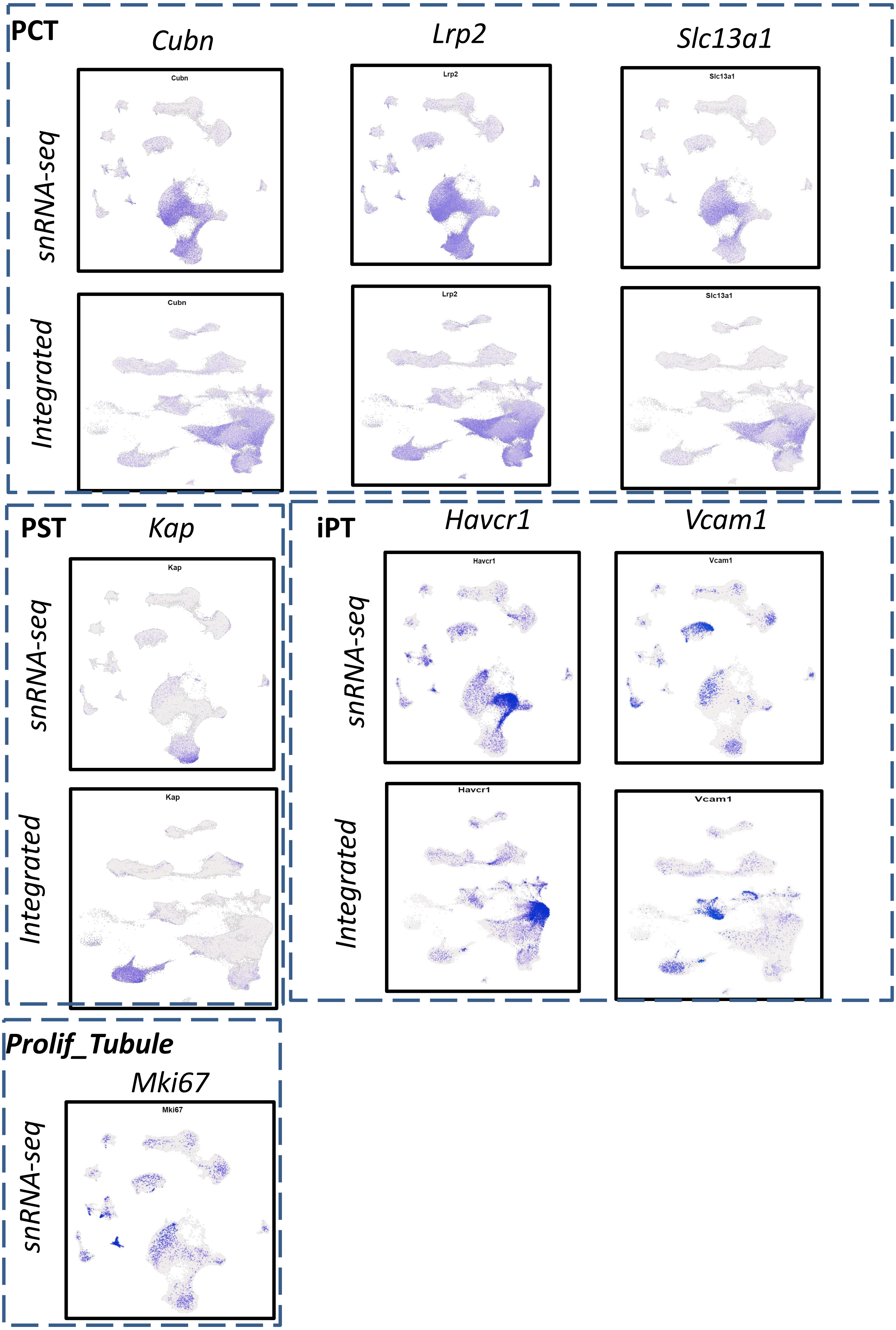

Supplementary Fig. 13. Feature plots of gene markers used to annotate different types of proximal tubules and proliferating tubules in the snRNA-seq and integrated snRNA-seq and snATAC-seq datasets.

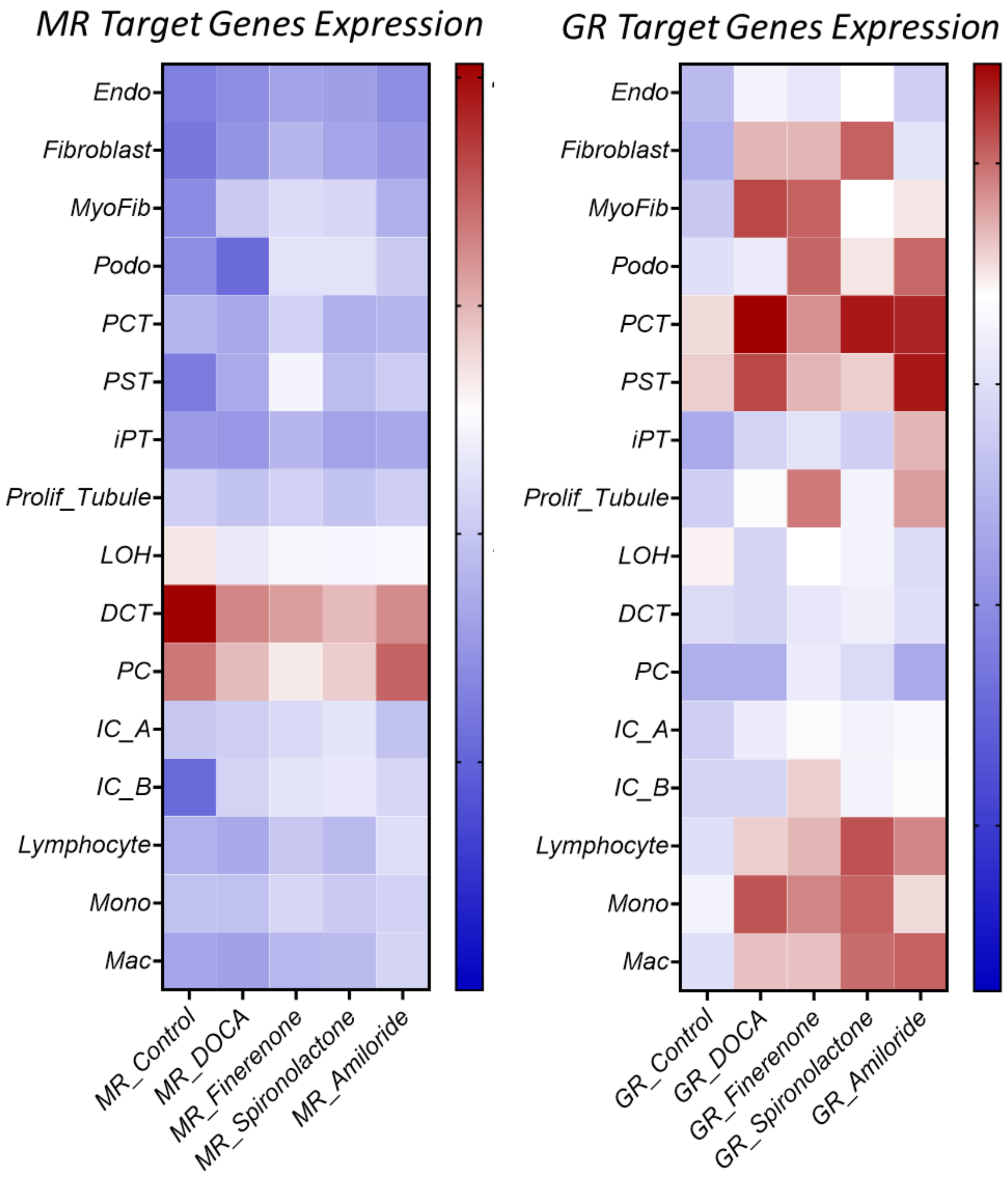

Supplementary Fig. 14. Heatmaps of the average expression of MR and GR target genes in different cell types and groups. The heatmaps show normalized z-score expression of MR and GR target genes. Endo; endothelial cells, MyoFib; myofibroblast, Podo; podocyte, PCT; proximal convoluted tubule, PST; proximal straight tubule, iPT; injured proximal tubule cells, Prolif_Tubule; proliferative tubule cells, LOH; loop of Henle, DCT; distal convoluted tubule; PC; principal cells of collecting ducts, IC; intercalated cells, IC_A; Type A intercalated cells, IC_B; Type B intercalated cells, Mono; monocyte, Macro; macrophage.

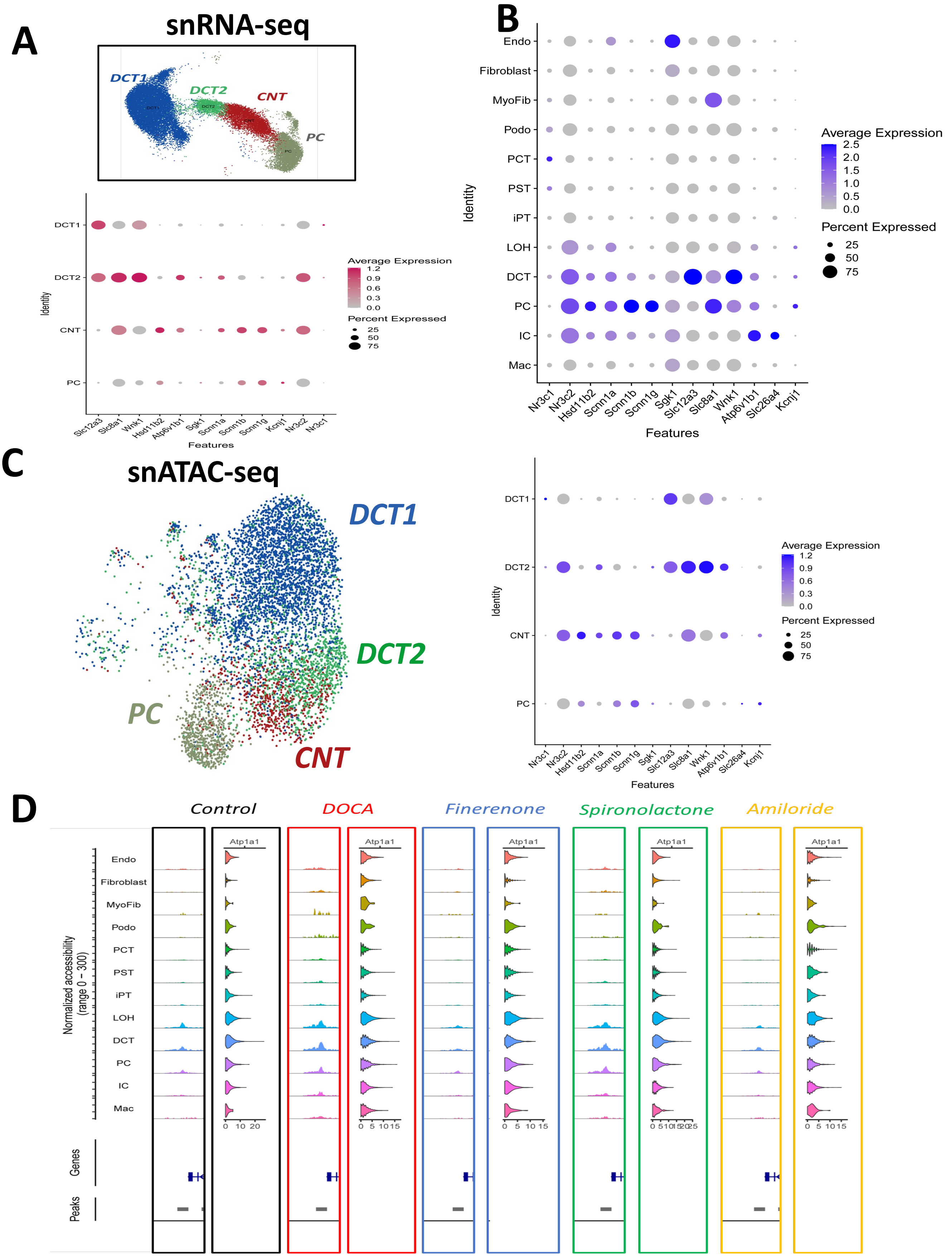

Supplementary Fig. 15. Mineralocorticoid target gene expression in snRNA-seq and snATAC-seq datasets. (A) UMAP of DCT and PC cells after sub-clustering in the snRNA-seq dataset (upper panel). Bubble dot plot of marker genes used in the sub-clustering of DCT and PC cells (lower Panel). The size of the dot indicates the percentage of positive cells, and the darkness of the color indicates average expression. **(B)** Bubble dot plot of mineralocorticoid target genes as well as glucocorticoid receptor (Nr3c1) in the snATAC-seq dataset. The size of the dot indicates expression percentage, and the darkness of the color indicates average expression. **(C)** UMAP of DCT and PC cells after sub-clustering in snATAC-seq dataset (left panel). Bubble dot plot of marker genes used in the sub-clustering of DCT and PC cells (right panel). The size of the dot indicates the percent of positive cells, and the darkness of the color indicates average expression. **(D)** Fragments coverage of the *Atp1a1* gene at the promoter region in different cell types and groups and its calculated gene activity. Endo; endothelial cells, MyoFib; myofibroblast, Podo; podocyte, PCT; proximal convoluted tubule, PST; proximal straight tubule, iPT; injured proximal tubule cells, Prolif_Tubule; proliferative tubule cells, LOH; loop of Henle, DCT; distal convoluted tubule; PC; principal cells of collecting ducts, IC; intercalated cells, Mac; macrophage.

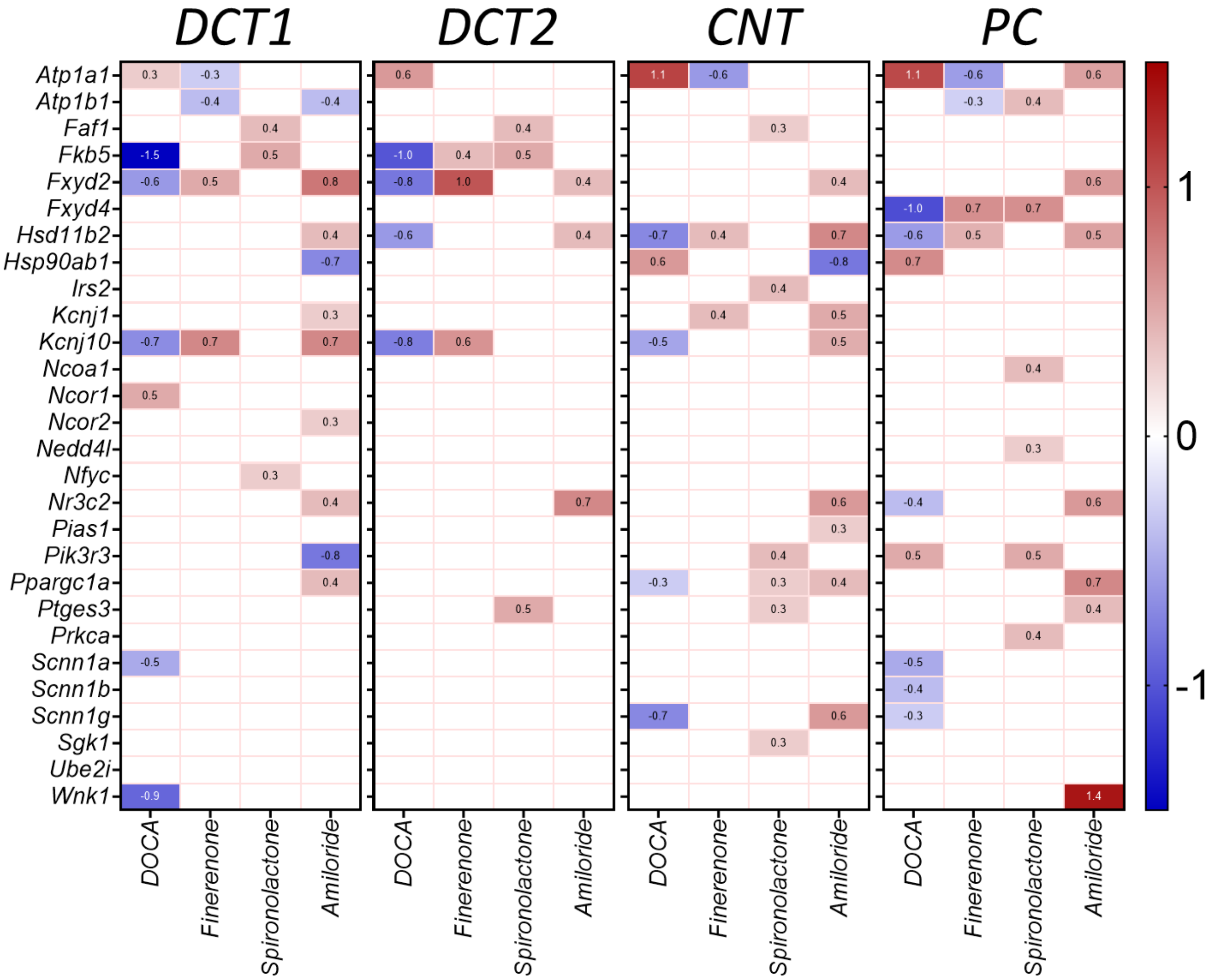

Supplementary Fig. 16. Gene expression changes following DOCA and drug treatments. Data shows log fold change gene expression between control and DOCA and DOCA vs treatment groups. Only genes showing significant changes are displayed. CNT; connecting tubule cells, DCT1; Type 1 distal convoluted tubule, DCT2; Type 2 distal convoluted tubule.

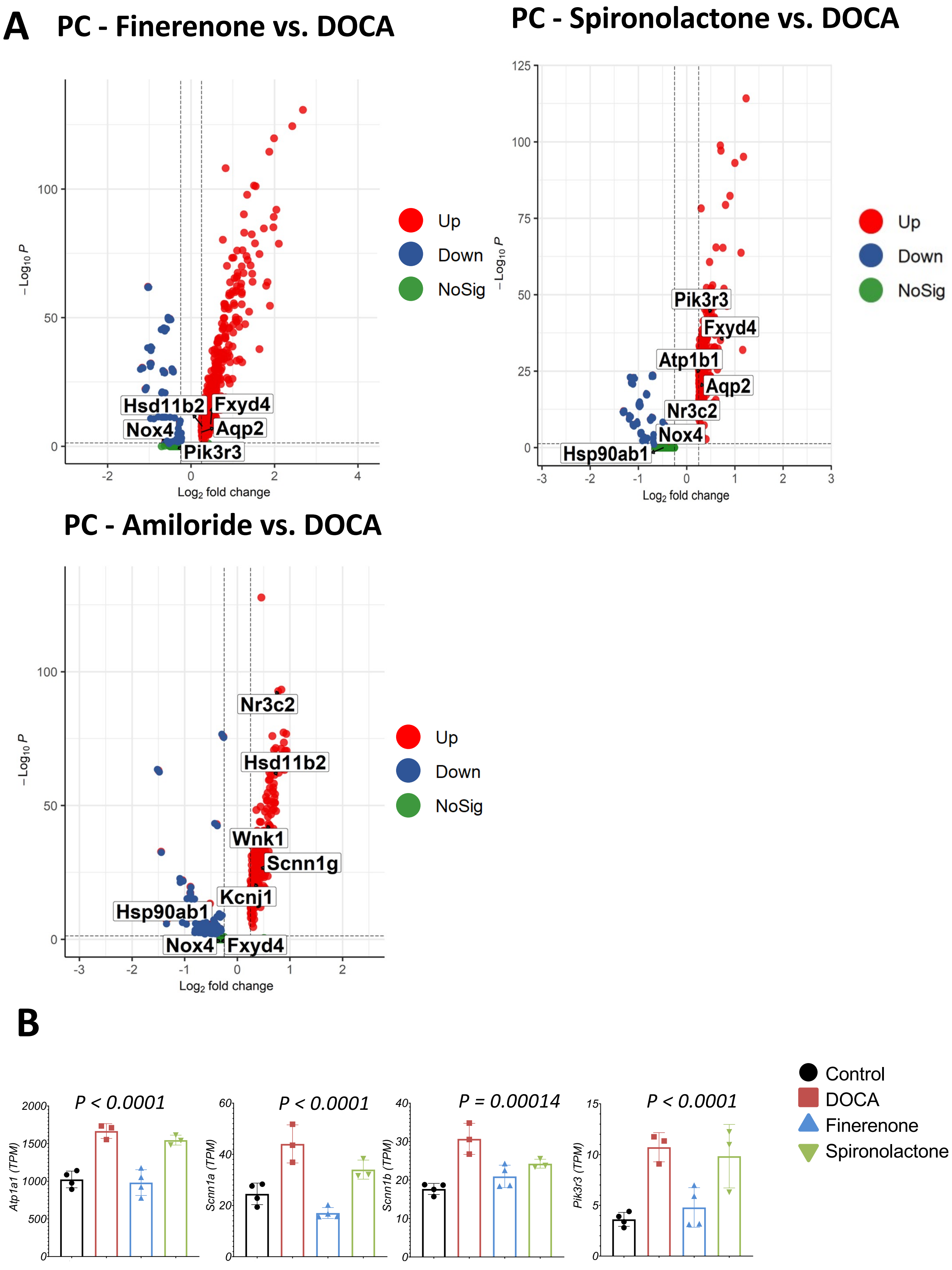

Supplementary Fig. 17. Targets of mineralocorticoid receptor antagonists. (A) Volcano plot of differentially expressed genes in principal cells of collecting ducts in finerenone treated vs. DOCA, spironolactone treated vs. DOCA, and amiloride treated vs. DOCA. **(B)** Expression of Atp1a1, Scnn1a, Scnn1b, and Pik3r3 in bulk RNA-seq datasets in different groups. The bar represents SEM. The comparison between groups were done using One-Way ANOVA test.

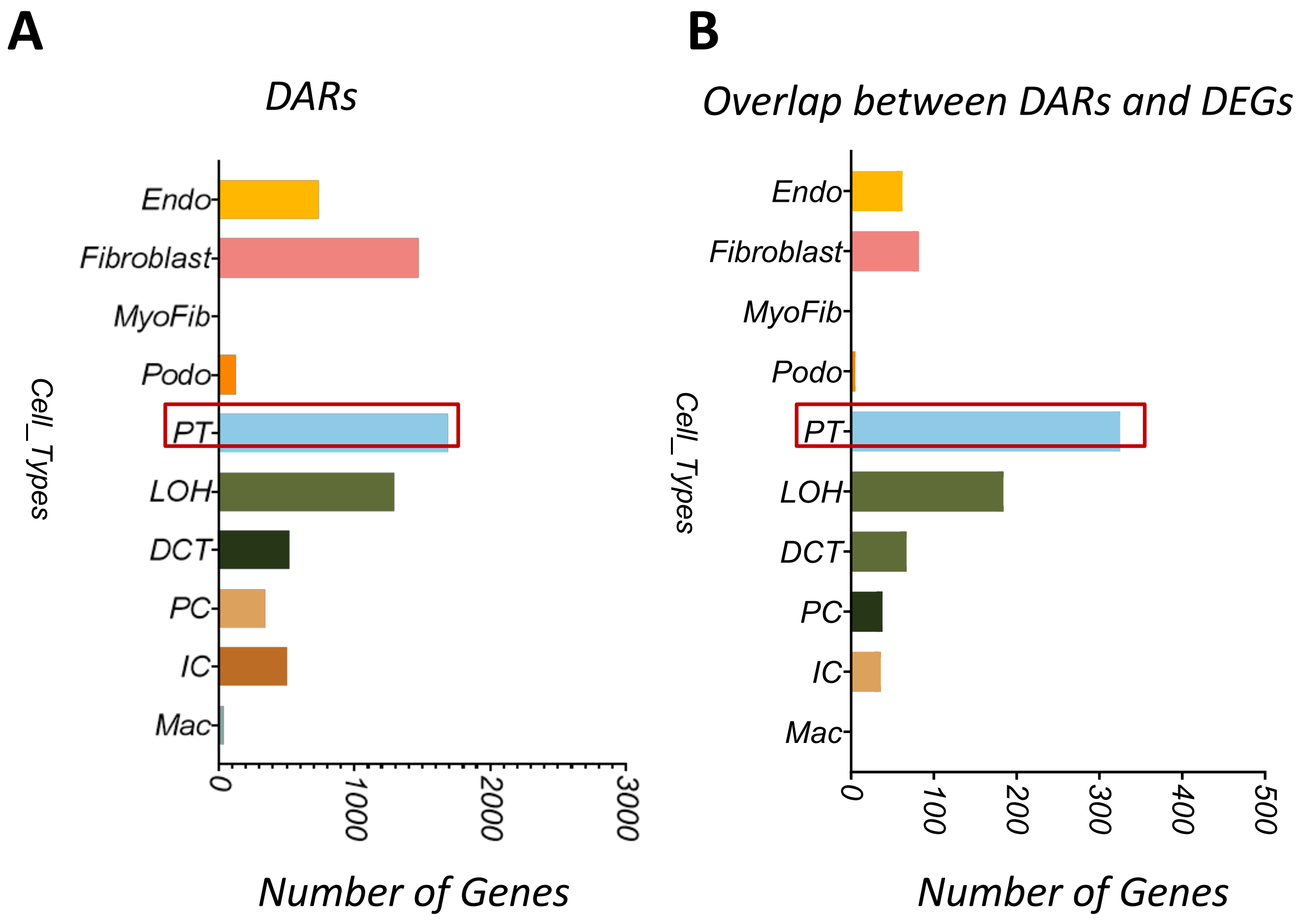

Supplementary Fig. 18. Differentially accessible regions analysis in the DOCA rat model. (A) The number of differentially accessible regions (DARs) between DOCA treated and control groups in all cell types after DOCA administration. (**B**) The number of overlapping genes identified by DARs and differentially expressed genes (DEGs) analysis. Endo; endothelial cells, MyoFib; myofibroblast, Podo; podocyte, PCT; proximal convoluted tubule, PST; proximal straight tubule, iPT; injured proximal tubule cells, Prolif_Tubule; proliferative tubule cells, LOH; loop of Henle, DCT; distal convoluted tubule; PC; principal cells of collecting ducts, IC; intercalated cells, Mac; macrophage.

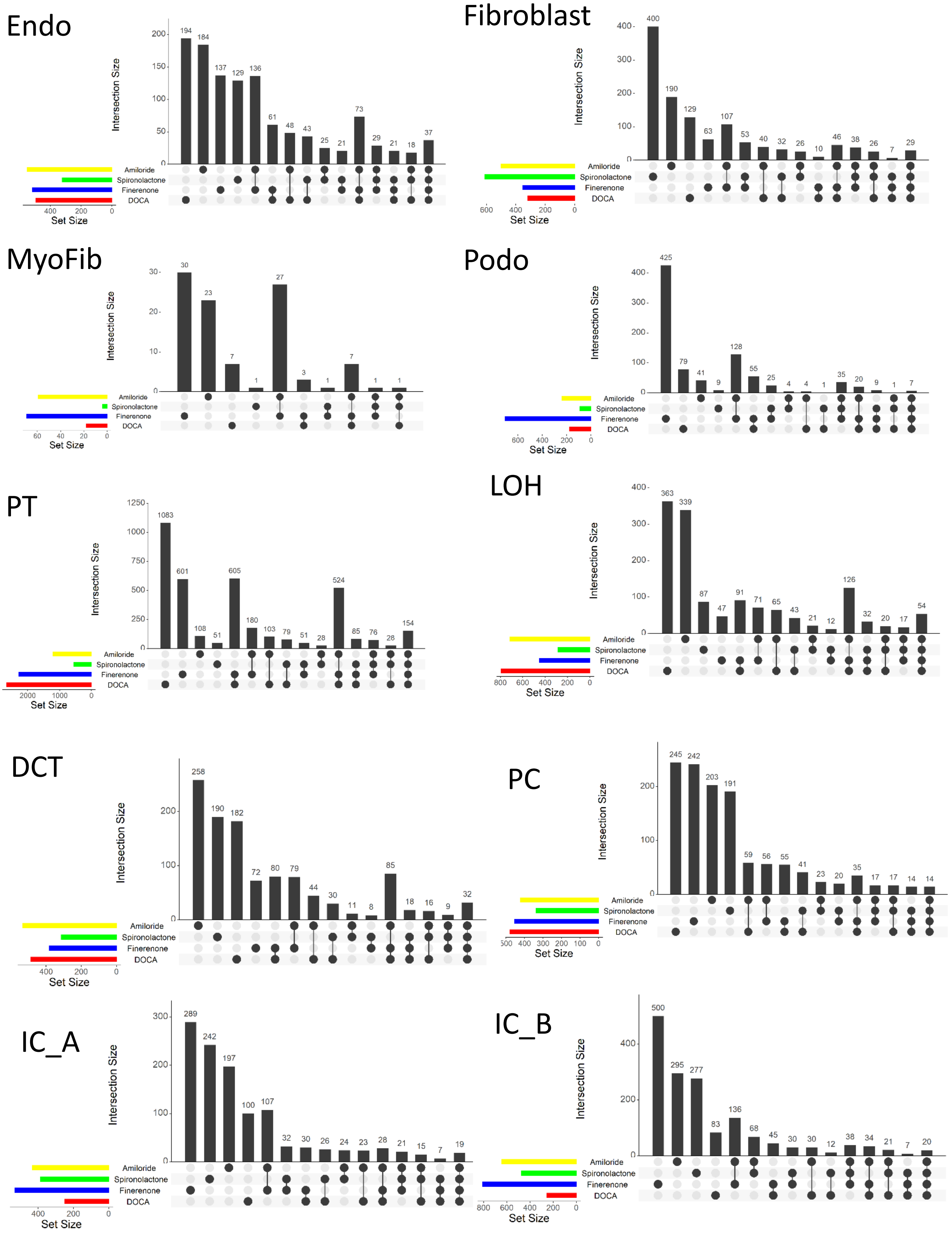

Supplementary Fig. 19. Differentially expressed genes and their overlap in different groups in the snRNA-seq. UpSet plot of the DEGs between groups in each cell types in the snRNA-seq. For each plot, first the DEGs between DOCA and control were calculated, then the DEGs between treatment groups and DOCA were compared.

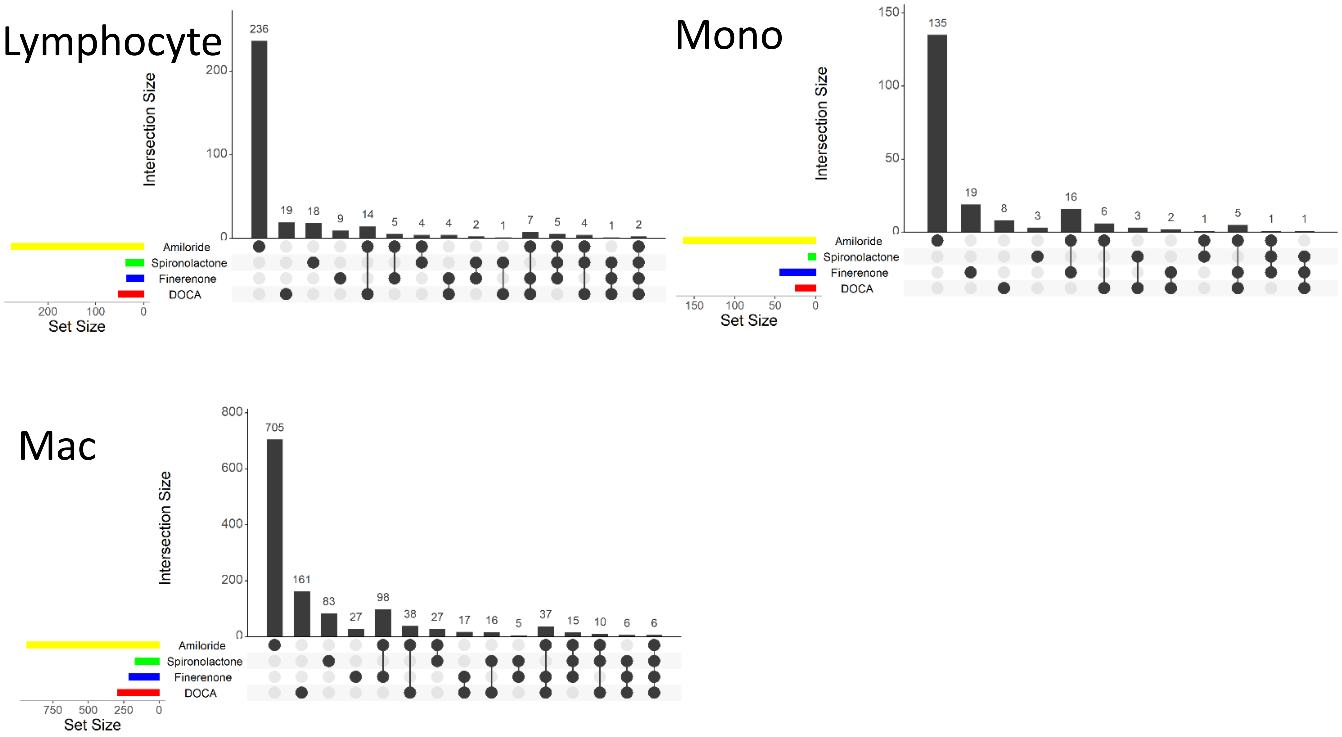

Supplementary Fig. 20. Differentially expressed genes and their overlaps in immune cells UpSet plots of DEGs between groups in immune cells in snRNA-seq. For each plot, first the DEGs between DOCA and control were calculated, then, the DEGs between treatment groups and DOCA were compared.

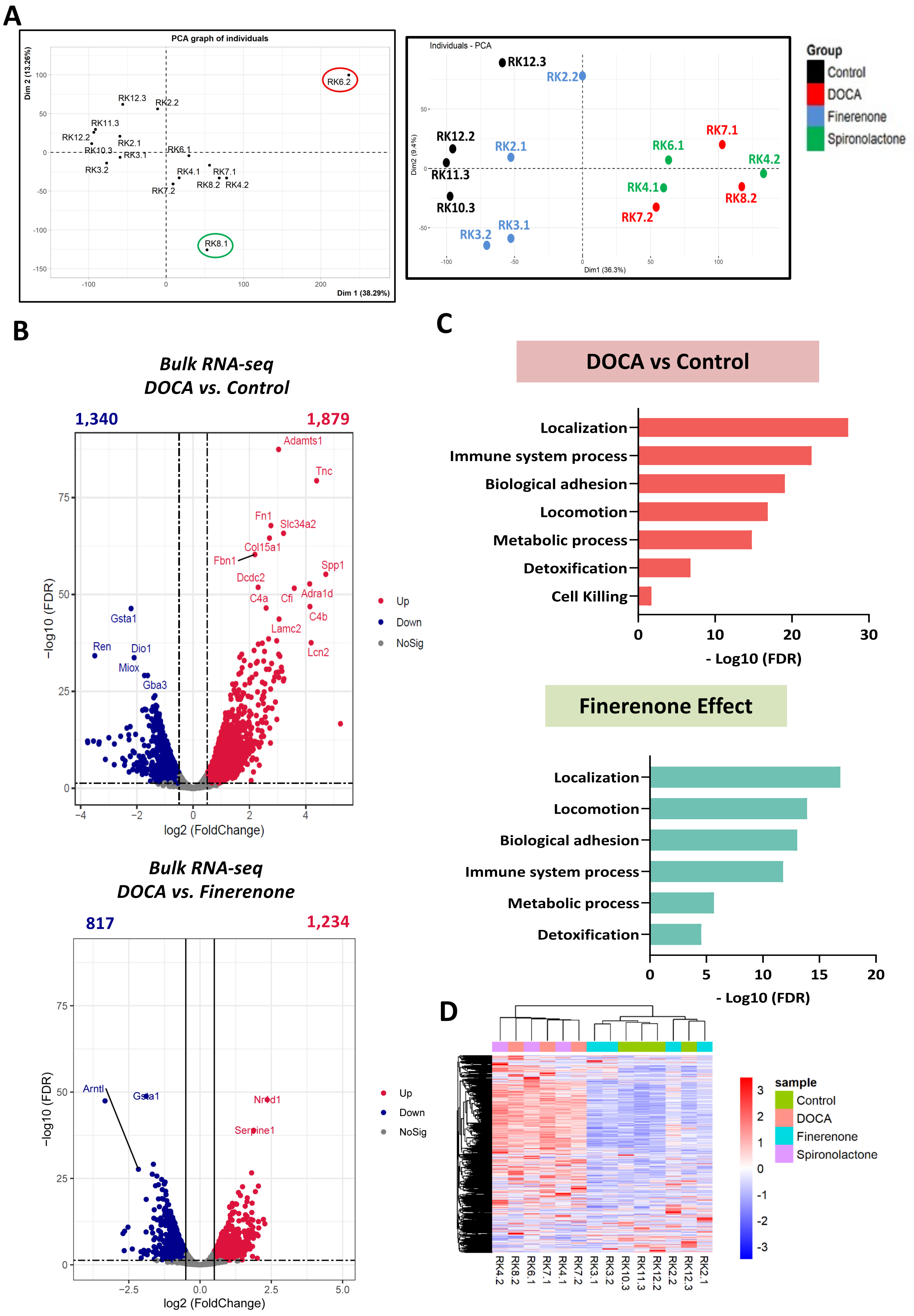

Supplementary Fig. 21. Bulk RNA-seq analysis of rat models. (A) PCA plot of different samples. Two outliers (one DOCA treated and one spironolactone) were identified. The right panel represents the PCA after removing the outliers. **(B)** Volcano plot of differentially expressed genes in bulk RNA-seq between DOCA treated vs. control and finerenone-treated groups. The red and blue numbers indicate the number of up-regulated and down-regulated genes. **(C)** Biological pathway analysis of the top 1000 DEGs between DOCA treated vs. control groups (upper panel) and the same analysis of genes affected by finerenone (lower panel) using DAVID. **(D)** Hierarchical clustering of the studied samples based on all genes in bulk RNA-seq. The plot shows that the finerenone-treated and control groups clustered together, while DOCA clustered with the spironolactone group.

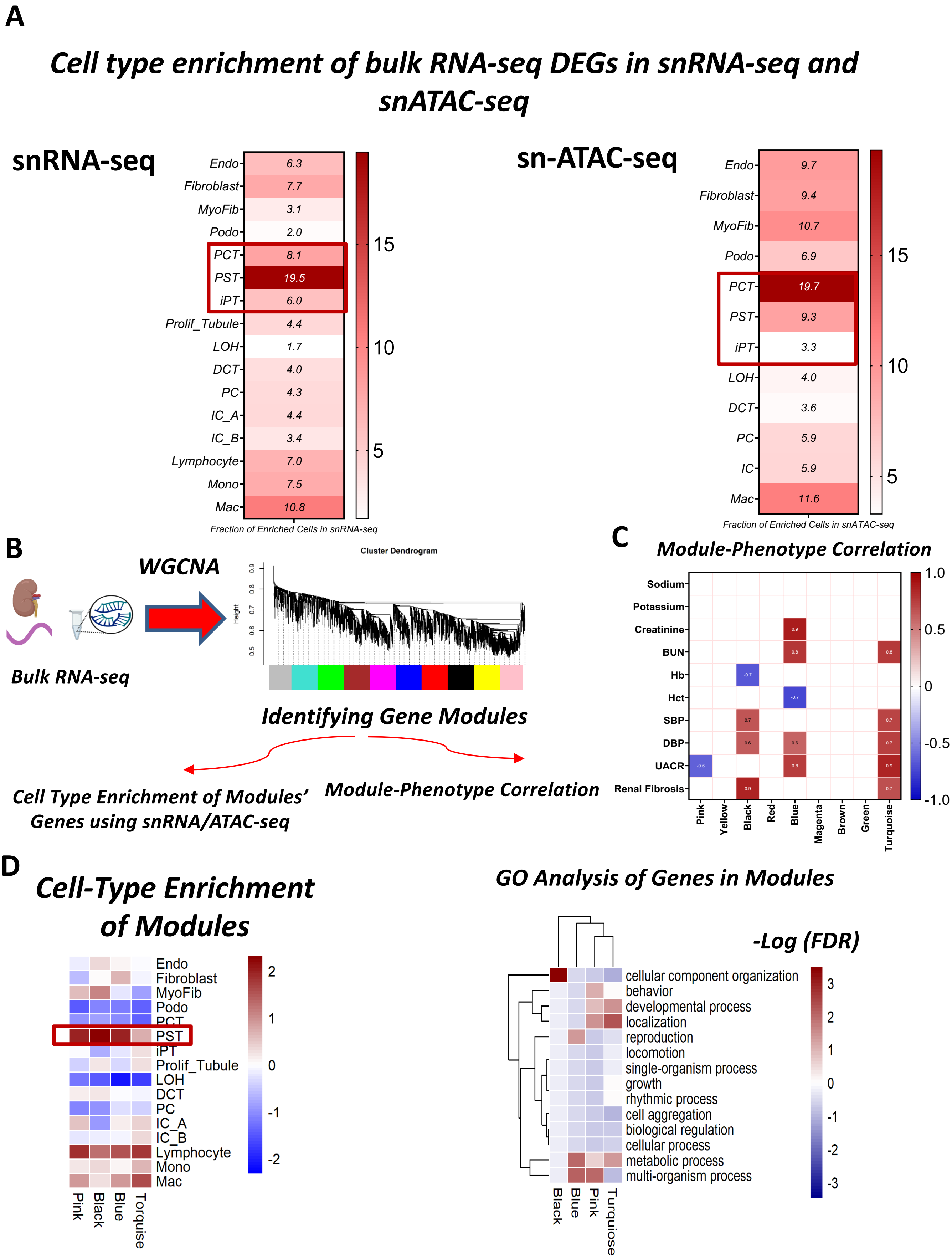

Supplementary Fig. 22. Cell type enrichment of bulk RNA-seq DEGs in the snRNA-seq data. (A) Cell type enrichment of bulk RNA-seq DEGs between DOCA and control in the snRNA-seq and snATAC-seq. For this analysis, the DEGs list of DOCA and control was compared to gene expression in snRNA-seq and snATAC-seq and the gene with highest value in one cell type was considered as the specific for that cell type. The results are shown as the fractions of specific genes in each cell type. **(B)** WGCNA of the bulk RNA-seq to identify phenotype specific modules and their specific cell types. **(C)** Heatmap of correlations between different modules and phenotypes. **(D)** Heatmap of the enrichment of significant modules obtained from bulk RNA-seq in identified cell types. For this analysis, the genes in each module were compared with the average expression of snRNA-seq data, and the gene with the highest expression was considered specific for that cell type. PST was enriched for all modules (left panel). The right panel shows the biological pathways enriched in each module using DAVID. Endo; endothelial cells, MyoFib; myofibroblast, Podo; podocyte, PCT; proximal convoluted tubule, PST; proximal straight tubule, iPT; injured proximal tubule cells, Prolif_Tubule; proliferative tubule cells, LOH; loop of Henle, DCT; distal convoluted tubule; PC; principal cells of collecting ducts, IC; intercalated cells, IC_A; Type A intercalated cells, IC_B; Type B intercalated cells, Mono; monocyte, Macro; macrophage.

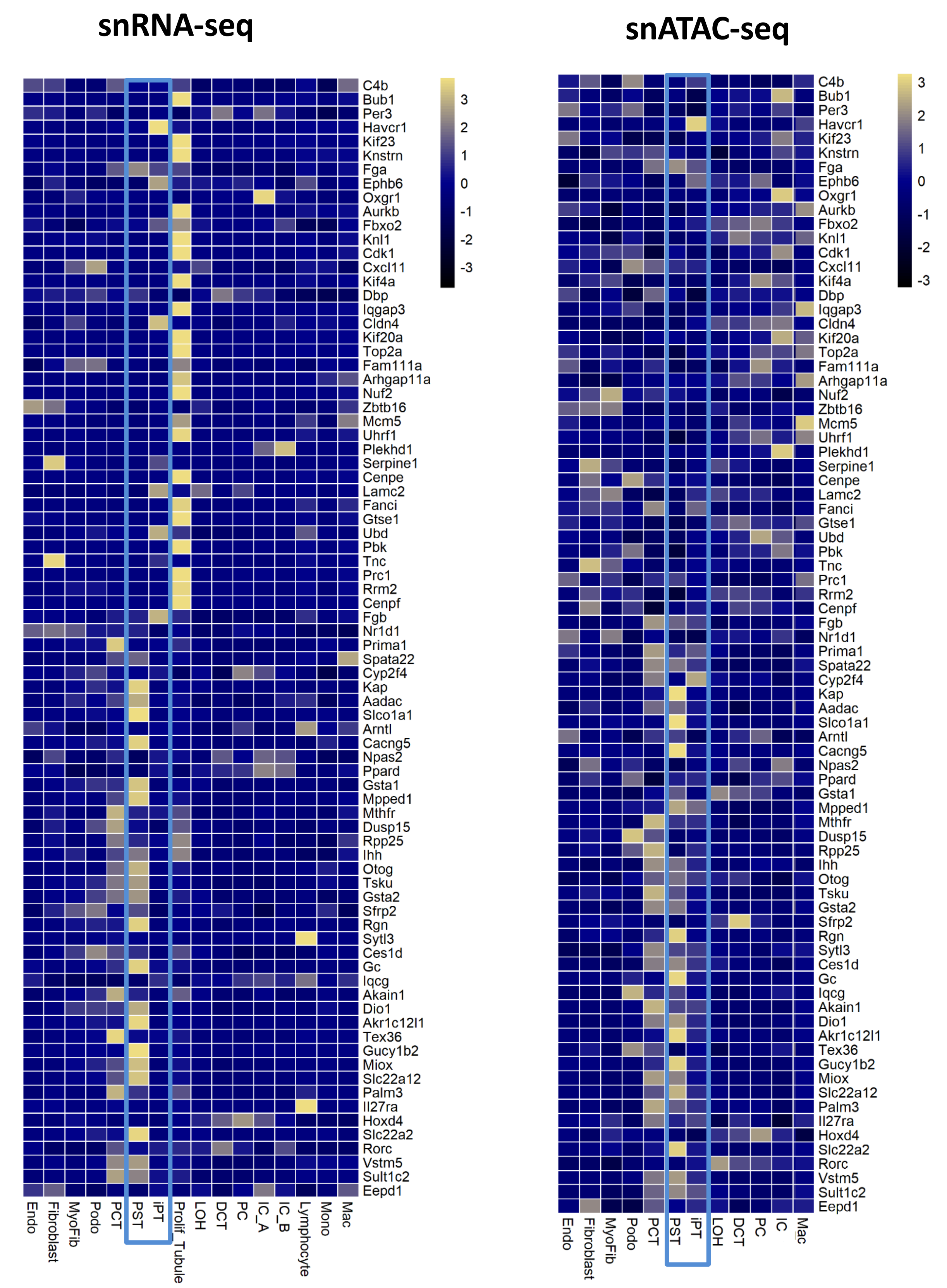

Supplementary Fig. 23. Enrichment of finerenone affected genes in snRNA-seq and snATAC-seq data. The analysis shows the up- and down-regulated genes by finerenone in bulk RNA-seq datasets enriched in PST, iPT, using the snRNA-seq and snATAC-seq datasets. Endo; endothelial cells, MyoFib; myofibroblast, Podo; podocyte, PCT; proximal convoluted tubule, PST; proximal straight tubule, iPT; injured proximal tubule cells, Prolif_Tubule; proliferative tubule, LOH; loop of Henle, DCT; distal convoluted tubule; PC; principal cells of collecting ducts, IC; intercalated cells, IC_A; Type A intercalated cells, IC_B; Type B intercalated cells, Mono; monocyte, Mac; macrophage.

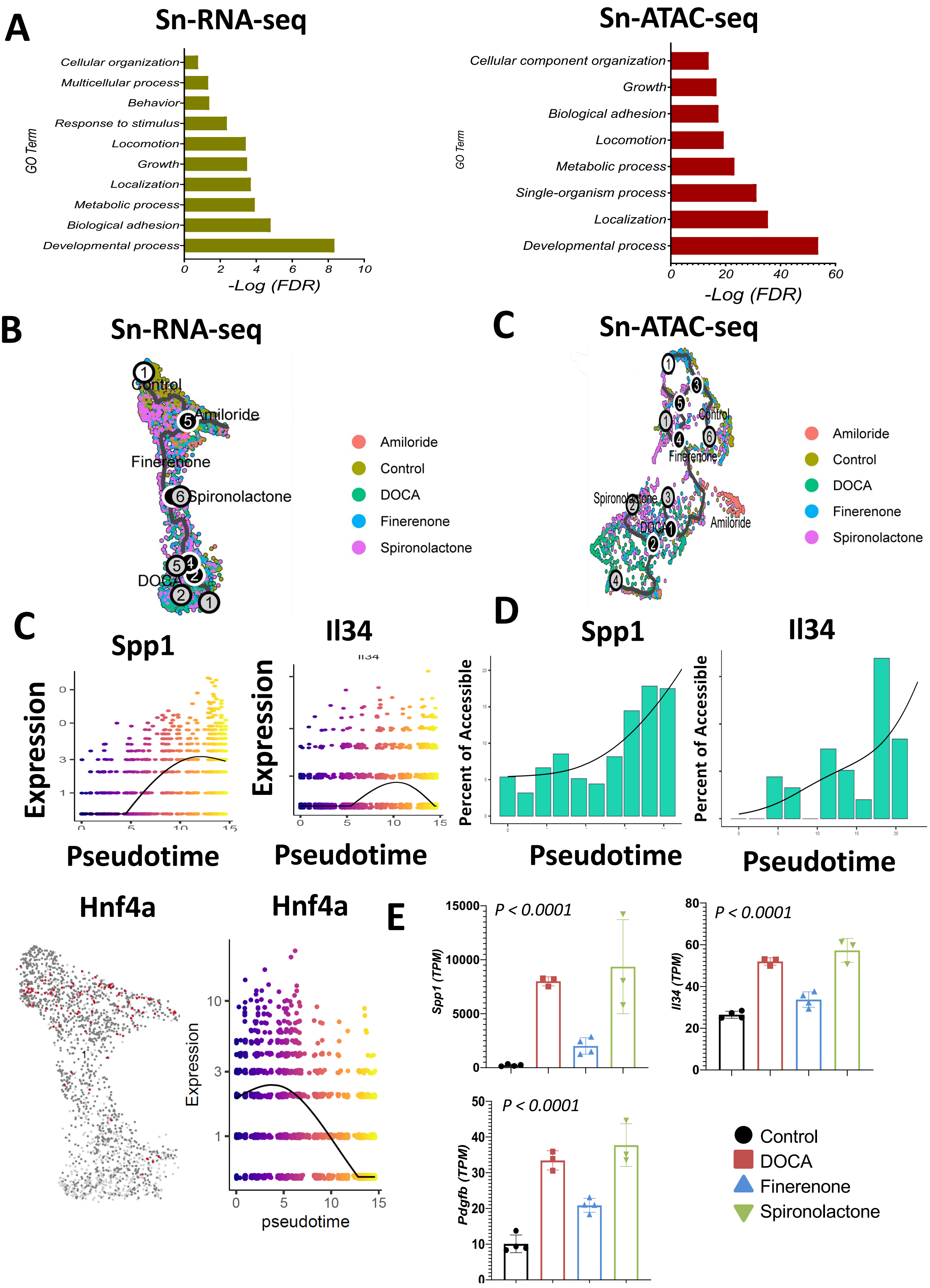

Supplementary Fig. 24. Trajectory analysis of PST and injured_PT sub-clusters in snRNA-seq and snATAC-seq datasets. (A) Biological pathway enrichment of the top driver genes of iPT differentiation using DAVID in snRNA-seq (left panel) and snATAC-seq (right panel). **(B)** UMAP of the trajectory of PST and iPT colored by their origins in snRNA-seq (left panel) and snATAC-seq (right panel) by different experimental groups along the trajectory. **(C)** Pseudotime plots of Spp1, Il34, and Hnf4a along the trajectory and feature plots of Hnf4a expression along the trajectory. **(D)** Percentage of chromatin accessibility regions of Spp1, Il34 along the trajectory pathway (lower panel). **(E)** Expression of Spp1, Il34, and Pdgfb in bulk RNA-seq in different groups. PST; proximal straight tubule, injured_PT; injured proximal tubule cells, DOCA; deoxycorticosterone acetate.

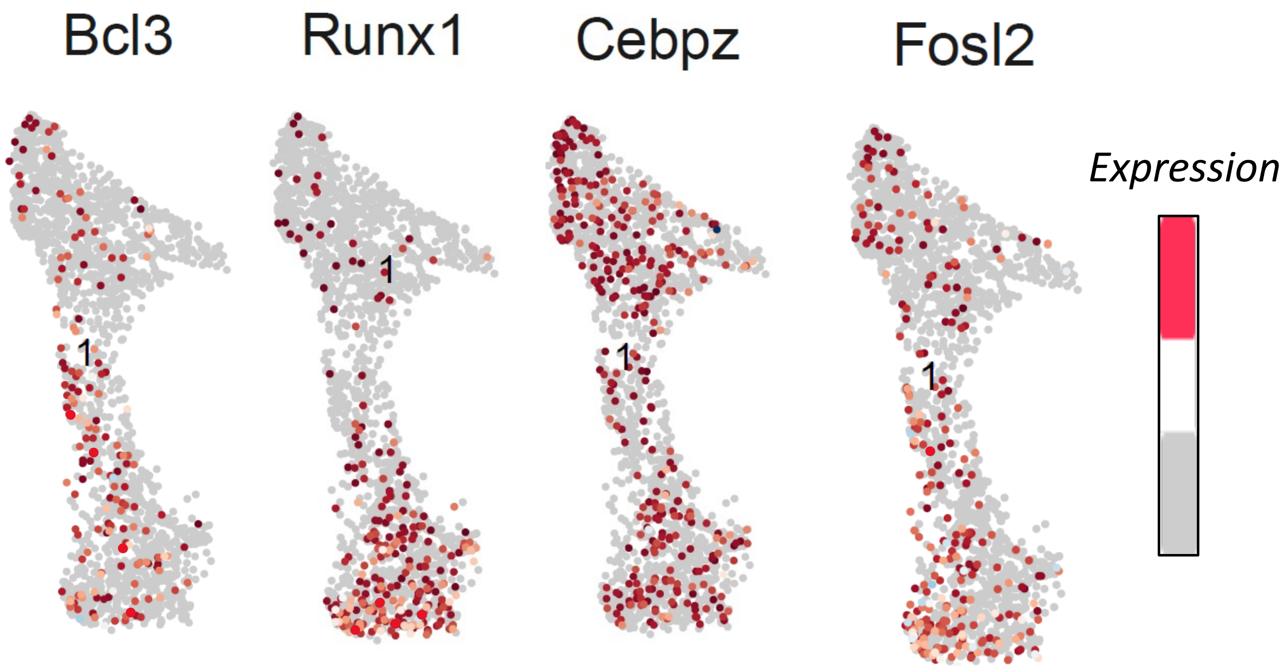

Supplementary Fig. 25. Feature plot of driver transcription factors for *Spp1*, *Il34, and Pdgfb* along the trajectory identified by SCENIC analysis. Red color shows the expression, gray represents no expression.

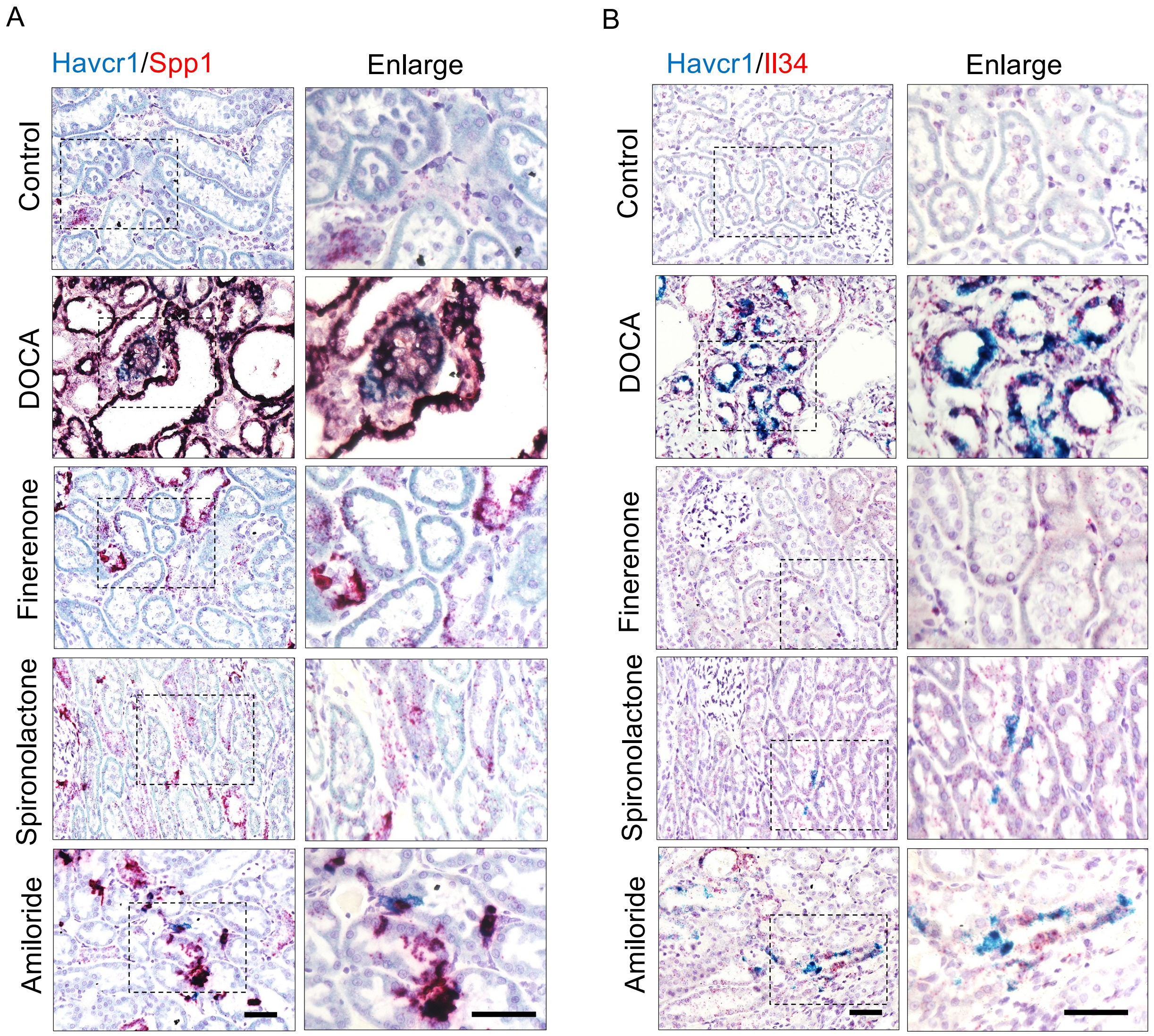

Supplementary Fig. 26. Expression patterns of *Spp1* and *Il34* in control and DOCA groups. Representative in situ hybridization images of *Spp1* (red), *Il34* (red), and *Havcr1* (blue) in the kidney of control and DOCA groups. Scale bar = 50 µm.

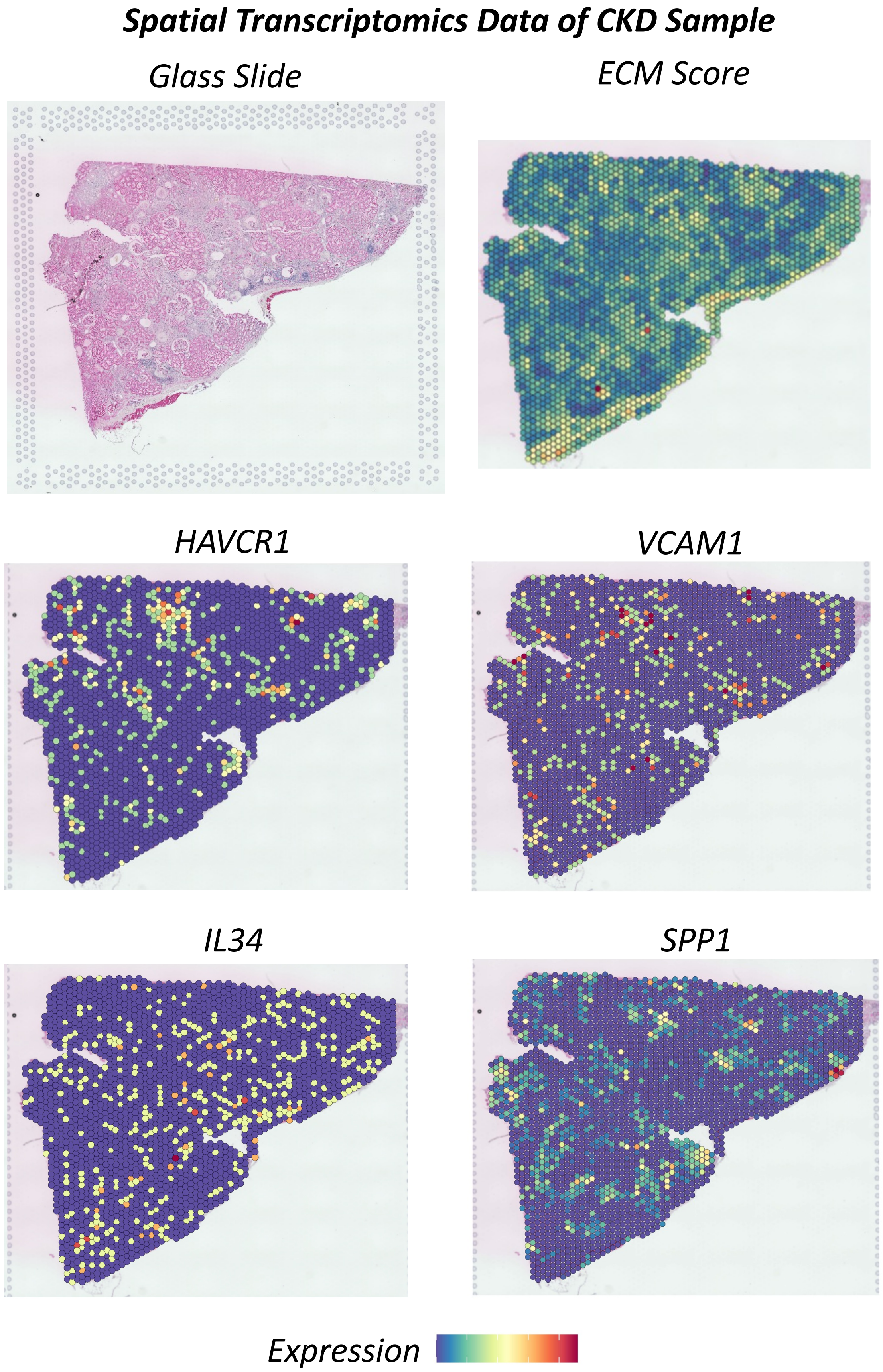

Supplementary Fig. 27. Co-expression of *HAVCR1*, *VCAM1*, *SPP1*, and *IL34* as the markers of injured PT cells in regions with a high extracellular matrix production score in the spatial transcriptomics of a human CKD sample. The ECM score is calculated based on the expression of extracellular matrix production genes. The image is 10X magnification.

Outgoing communication Patterns of Secreting Cells in Different Groups

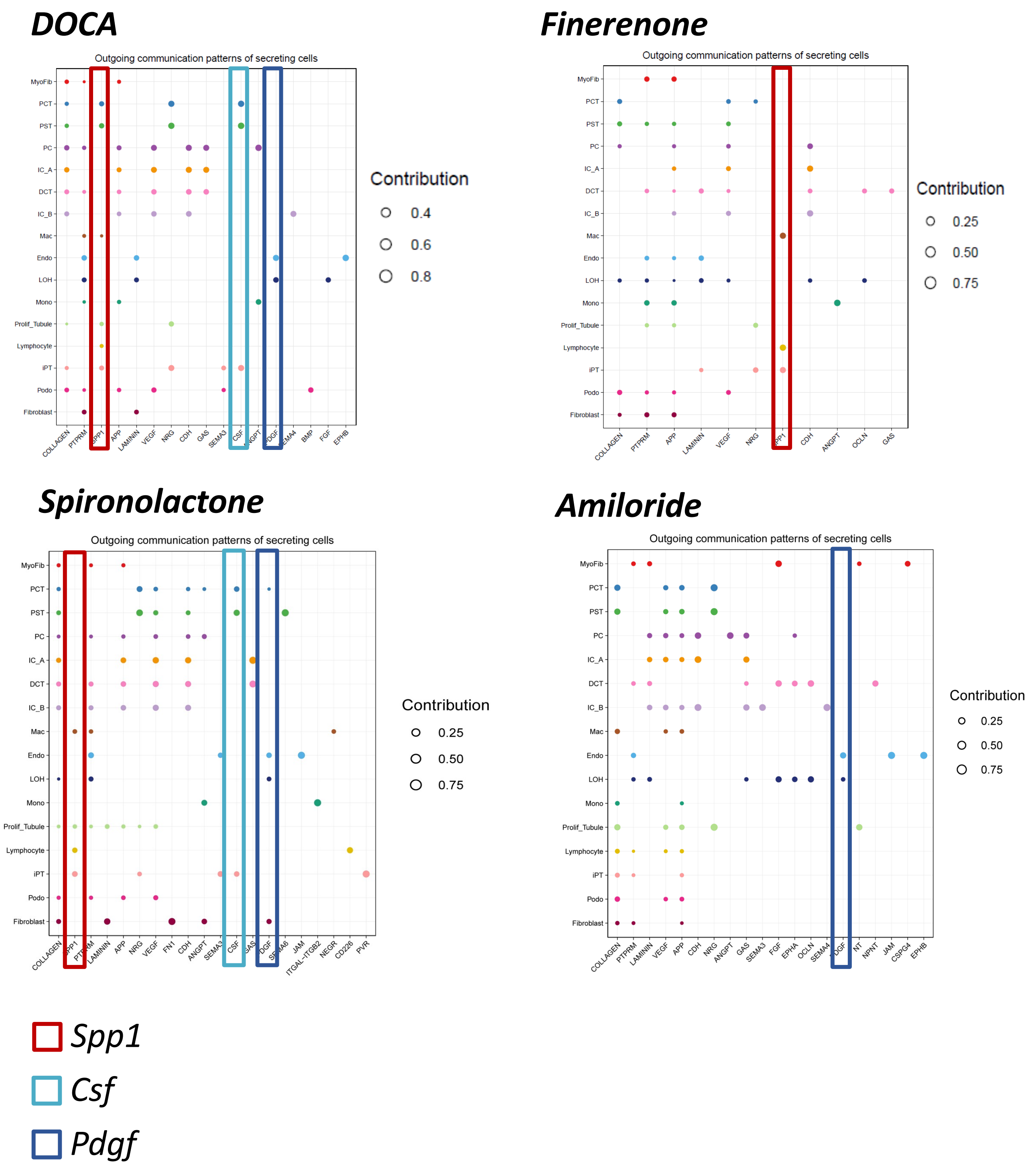

Supplementary Fig. 28. Outgoing communication Patterns of Secreting Cells in Different Groups. Bubble plot of the number of interactions in different experimental groups for different ligand-receptor interactions. Each dot shows the number of interactions at different ligand-receptors in different cell types.

Endo; endothelial cells, MyoFib; myofibroblast, Podo; podocyte, PCT; proximal convoluted tubule, PST; proximal straight tubule, iPT; injured proximal tubule cells, Prolif_Tubule; proliferative tubule cells, LOH; loop of Henle, DCT; distal convoluted tubule; PC; principal cells of collecting ducts, IC_A; Type A intercalated cells, IC_B; Type B intercalated cells, Mono; monocyte, Mac; macrophage.

Cell Types Contribution of Outgoing and Incoming Signals in Different Groups

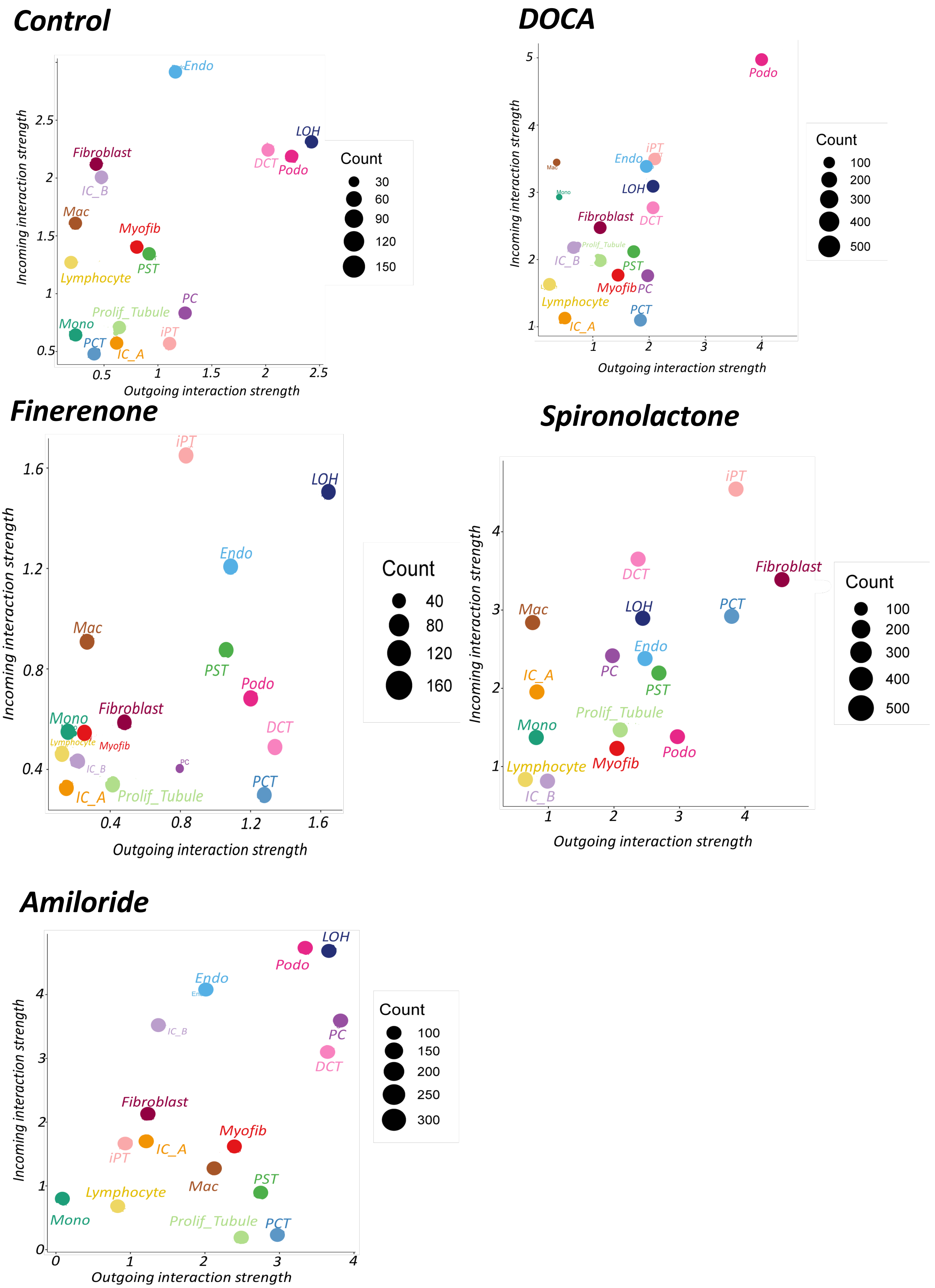

Supplementary Fig. 29. The plot of the outgoing and incoming interaction strength of the ligand-receptor interactions in different experimental groups. X-axis shows the strength of cell in producing the ligands and y-axis indicates the cells with enriched receptors of the ligands. Endo; endothelial cells, MyoFib; myofibroblast, Podo; podocyte, PCT; proximal convoluted tubule, PST; proximal straight tubule, iPT; injured proximal tubule cells, Prolif_Tubule; proliferative tubule cells, LOH; loop of Henle, DCT; distal convoluted tubule; PC; principal cells of collecting ducts, IC; intercalated cells, IC_A; Type A intercalated cells, IC_B; Type B intercalated cells, Mono; monocyte, Mac; macrophage.

A

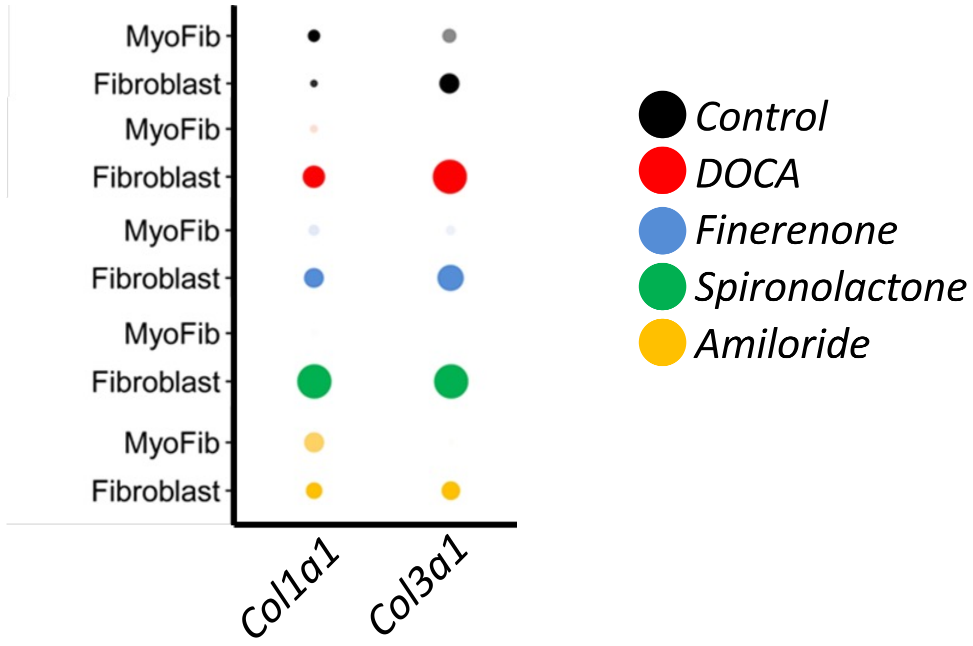

B

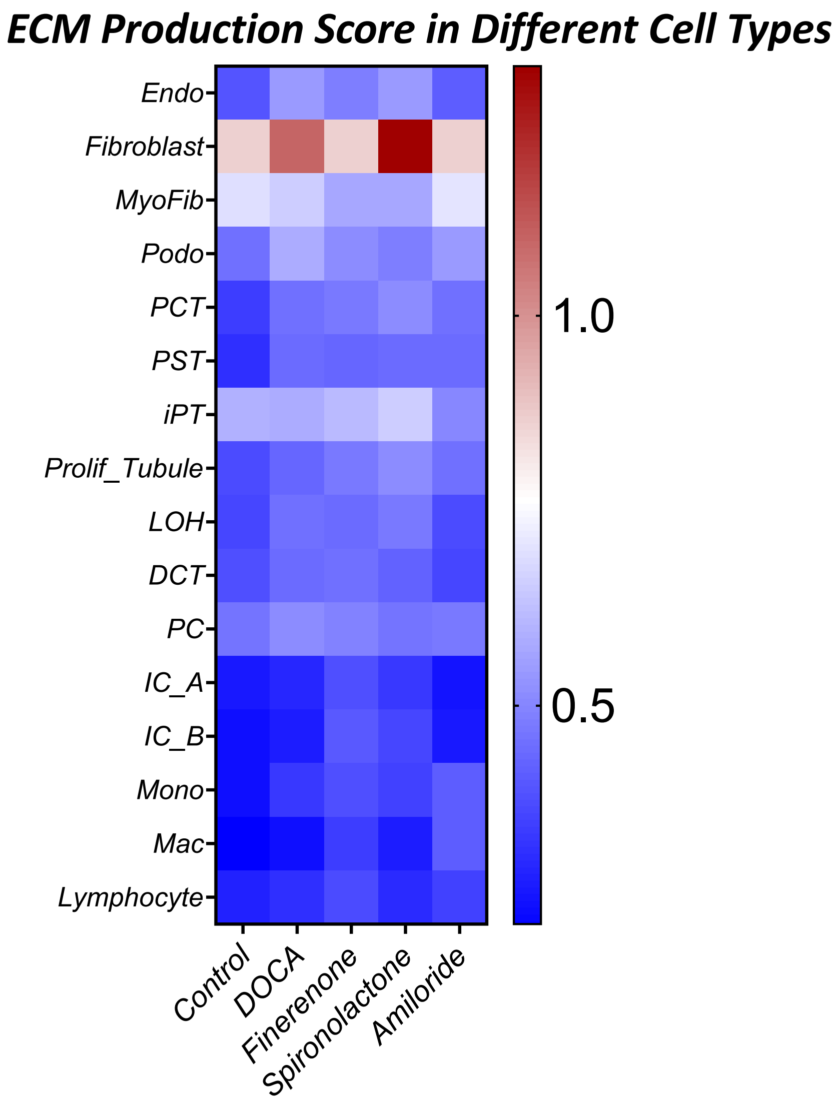

Supplementary Fig. 30. Expressions of extracellular matrix genes in the DOCA rat nephropathy model. (A) Bubble dot plot of expressions of *Col1a1 and Col3a1* in different experimental groups in fibroblasts and myofibroblasts **(B)** Heatmap of extracellular matrix production scores in different cell types and groups. The data is shown as average of the expression of 158 genes responsible for ECM production. The heatmap shows the z score of average expression. Endo; endothelial cells, MyoFib; myofibroblast, Podo; podocyte, PCT; proximal convoluted tubule, PST; proximal straight tubule, iPT; injured proximal tubule cells, Prolif_Tubule; proliferative tubule cells, LOH; loop of Henle, DCT; distal convoluted tubule; PC; principal cells of collecting ducts, IC_A; Type A intercalated cells, IC_B; Type B intercalated cells, Mono; monocyte, Mac; macrophage.

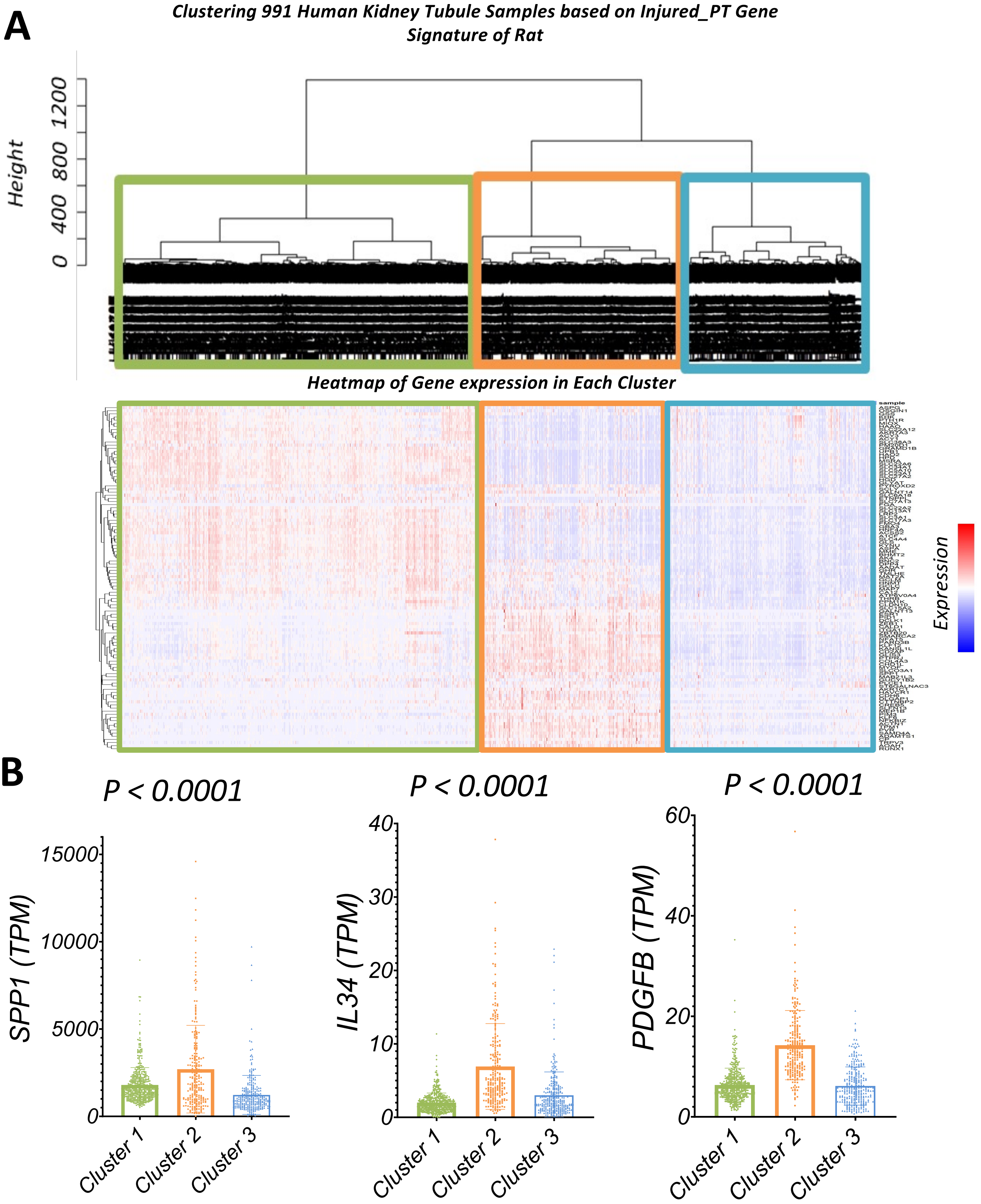

Supplementary Fig. 31. Clustering 991 human kidney microdissected tubules based on the PT gene signature in rats. (A) Heatmap of the gene expression patterns in three distinct clusters of 991 human kidney tubule samples. The heatmap indicates the z score of expression of each gene in each sample. (**B**) Gene expression pattern of *SPP1*, *IL34*, and *PDGFB* in three obtained clusters in human kidney tubule samples. P values were calculated using One-way ANOVA test. Bars indicate SD.
